# Spatial separation of catches in highly mixed fisheries

**DOI:** 10.1038/s41598-018-31881-w

**Published:** 2018-09-17

**Authors:** Paul J. Dolder, James T. Thorson, Cóilín Minto

**Affiliations:** 1 Marine and Freshwater Research Centre, Galway-Mayo Institute of Technology (GMIT), Dublin Road, Galway, H91 T8NW Ireland; 2 Centre for Environment, Fisheries and Aquaculture Science (Cefas), Pakefield Road, Lowestoft, Suffolk NR33 0HT United Kingdom; 3 Fisheries Resource Analysis and Monitoring Division, Northwest Fisheries Science Center, National Marine Fisheries Service, NOAA, 2725 Montlake Blvd E, Seattle, Washington 98112 USA

**Keywords:** Conservation biology, Population dynamics, Ecological modelling, Marine biology

## Abstract

Mixed fisheries are the dominant type of fishery worldwide. Overexploitation in mixed fisheries occurs when catches continue for available quota species while low quota species are discarded. As EU fisheries management moves to count all fish caught against quota (the “landing obligation”), the challenge is to catch available quota within new constraints, else lose productivity. A mechanism for decoupling exploitation of species caught together is spatial targeting, which remains challenging due to complex fishery and population dynamics. How far spatial targeting can go to practically separate species is often unknown and anecdotal. We develop a dimension-reduction framework based on joint dynamic species distribution modelling to understand how spatial community and fishery dynamics interact to determine species and size composition. In application to the highly mixed fisheries of the Celtic Sea, clear common spatial patterns emerge for three distinct assemblages. While distribution varies interannually, the same species are consistently found in higher densities together, with more subtle differences within assemblages, where spatial separation may not be practically possible. We highlight the importance of dimension reduction techniques to focus management discussion on axes of maximal separation and identify spatiotemporal modelling as a scientific necessity to address the challenges of managing mixed fisheries.

## Introduction

## Mixed fisheries and the EU landing obligation

Recent efforts to reduce exploitation rates in commercial fisheries have begun the process of rebuilding depleted fish populations^1^. Improved management of fisheries has the potential to increase population sizes and allow increased sustainable catches, yet fisheries catch globally remains stagnant^2^. In light of a projected increase in demand for fish protein^3^ there is an important role for well managed fisheries in supporting future food security^4^ necessitating that fisheries are managed efficiently to maximise productivity.

A particular challenge in realising increased catches from rebuilt populations is maximising yields from mixed fisheries^5–7^. In mixed fisheries, the predominant type of fishery worldwide, several fish species are caught together in the same net or fishing operation (known as a “technical interaction”). If managed by individual quotas, and catches do not match available stock quotas, either a vessel must stop fishing when the first quota is reached (the “choke” species) or overexploitation of the weaker species occurs while fishers continue to catch more healthy species and throw back (“discard”) the fish for which they have no quota^8^. There is, therefore, a pressing need for scientific tools to simplify the complexities of mixed fisheries and help avoid discarding.

Sustainability of European fisheries has been hampered by the “mixed fishery problem” for decades with large-scale discarding resulting^9,10^. Mixed fisheries require specific management approaches to avoid overfishing and a paradigm shift is being introduced under the EU Common Fisheries Policy (CFP) reform of 2012 through two significant management changes. First, by 2019 all fish that are caught are due to be counted against the respective stock quota even if they are discarded; second, by 2020 all fish stocks must be fished at an exploitation rate corresponding to their Maximum Sustainable Yield (MSY)^11^. These changes are expected to contribute to attainment of the goal of Good Environmental Status (GES) under the European Marine Strategy Framework Directive (MSFD^12^) and move Europe towards an ecosystem based approach to fisheries management^13^.

Conflicts between overall management goals and drivers for individual actors must be overcome to achieve sustainability. Societal objectives for fisheries to achieve MSY across ecosystem components are paralleled by individual fishers goals to maximise utility; whether that be profit, income or the continuance of traditional practices^14^. Under the new policy, unless fishers can avoid catch of unwanted species they will have to stop fishing when reaching their first restrictive quota. This introduces a potential significant cost to fishers of under-utilised quota^7,15^ and provides a strong incentive to mitigate such losses^16,17^.

To align catch with available quota depends on the ability to exploit target species while avoiding unwanted catch. Methods by which fishers can alter their fishing patterns include switching fishing method (e.g. trawling to netting), changing technical gear characteristics (e.g. introducing escapement panels in nets), or altering the timing and location of fishing activity^18,19^. For example, otter trawl gears are known to have higher catch rates of roundfish due to the higher headline and wider sweeps, which herd demersal fish into the net; conversely, beam trawls that employ chain mesh to “dig” benthic flatfish species, have higher catch rates for these species^20^. Fishing location choice also has a significant effect on catch^21^, something that fishers routinely consider in their decision making based on their own knowledge of good fishing locations.

In the past, spatiotemporal management measures (such as time-limited fishery closures) have been applied to reduce unwanted catch with varying degrees of success (e.g.^22–25^) while move-on rules have also been proposed or implemented to influence catch rates of particular vulnerable species to reduce or eliminate discards (e.g.^26–28^). However, such measures have generally been targeted at individual species without considering associations and interactions among several species. Highly mixed fisheries are complex with spatial, technological and community interactions combining. The design of spatiotemporal management measures that aim to allow exploitation of high quota stocks while protecting low quota stocks requires understanding these interactions at a scale meaningful to managers and fishers. While fisheries surveys and commercial fishing routinely generate a large amount of geo-referenced information on numbers and weight of fish caught, integrating spatiotemporal information from across multiple sources of fisheries-dependent and independent survey data requires an effective framework to reduce and understand the complexities of the system.

Here, our goal is to develop a framework for understanding these complexities. We do so by (1) implementing a spatiotemporal dimension reduction method that estimates the correlation in catches for multiple species at each fishing location, (2) using the results to draw inference on the fishery-community dynamics, (3) creating a framework to identify common trends among species, and (4) describing the potential for and limitation of spatial measures to mitigate unwanted catches in highly mixed fisheries.

## Framework for analysing spatiotemporal mixed fisheries interactions

We present a framework for analysing how far spatiotemporal avoidance can contribute towards mitigating imbalances in quota in mixed fisheries. Fisheries-independent survey data are used to characterise the spatiotemporal dynamics of key components of a fish community by employing a geostatistical Vector Autoregressive Spatiotemporal model (VAST). Therein, a factor analysis decomposition was used to describe trends in spatiotemporal dynamics of the different species as a function of latent variables^29^ representing spatial variation (9 factors; termed “average” spatial variation) and spatiotemporal variation (9 factors) for encounter probability and positive catch rates (termed “positive density”) separately^30^. Resultant factor analyses identify community dynamics and drivers common among 9 species, each analysed separately for juvenile and adult stages. We refer to each combination of species and size class as a “species”, and present results for the 18 species through transformation of the loading matrices using PCA rotation. This PCA rotation is used to visualise a reduced number of orthogonal factors representing average spatial variation or spatiotemporal variation while explaining the majority of covariation among catch rates, as well as the association of each species with these maps. We refer to the association of each species with a given factor as its “association with this factor”, and the value of each factor at a given location as its “ ‘coefficient’ at that location”. By describing the species dynamics through underlying spatiotemporal factors we can take account of how the factors contribute to affect catches of the species in mixed fisheries. Gaussian Markov Random Fields (GMRFs) capture spatial and temporal dependence within and among species for both encounter probability and positive density^31^. VAST is set in a mixed modelling framework which allows estimation of fixed effects to account for systematic differences driving encounter and catches, such as differences in sampling efficiency (catchability), while random effects capture the spatiotemporal dynamics of the fish community.

## Dynamics of Celtic Sea fisheries

The highly mixed demersal fisheries of the Celtic Sea are used as a case study. The Celtic Sea is a temperate sea where fisheries are spatially and temporally complex; mixed fisheries are undertaken by several nations using different gear types^21,32^. Close to 150 species have been identified in the commercial catches of the Celtic Sea, with approximately 30 species dominating the catch^33^.

Our spatiotemporal model is parametrised using catch data from seven fisheries-independent surveys undertaken in the Celtic Sea over the period 1990–2015 (Table S1) and include nine of the main commercial species: Atlantic cod (*Gadus morhua*), Atlantic haddock (*Melanogrammus aeglefinus*), Atlantic whiting (*Merlangius merlangus*), European Hake (*Merluccius merluccius*), white-bellied anglerfish (*Lophius piscatorius*), black-bellied anglerfish (*Lophius budegassa*), megrim (*Lepidorhombus whiffiagonis*), European plaice (*Pleuronectes platessa*) and common sole (*Solea solea*). These species comprise over 60% of landings by towed fishing gears for the area (average 2011–2015^34^). Each species was separated into juvenile and adult size classes based on their legal minimum conservation reference size (Table S2).

The data were analysed to understand how the different associations among species (combination of species and size class) form distinct assemblages with common drivers of spatiotemporal distributions, and how these affect catch compositions for fishers operating in mixed fisheries. We consider how these have changed over time, and the implications for mixed fisheries in managing catches of quota species under the EU landing obligation.

## Results

Using relatively few factors in a spatial dynamic factor analysis the Celtic Sea demersal fish community can be partitioned into three species assemblages (roundfish, flatfish and deeper water species). Within these assemblages there are common trends in spatiotemporal distributions in encounter probability and positive density, which can be partitioned into time invariant (“average effect”) spatial trends and time variant (“spatiotemporal”) trends. We show through presentation of factor coefficients that time invariant trends may be linked to physical characteristics of the system including depth and predominant substrate type, while species loadings on to time varying spatial trends show changes in distribution of species over time to be similar within an assemblage. We demonstrate how this information can be used to help inform spatial targeting and avoidance of the different assemblages. More nuanced differences in spatiotemporal distributions exist within an assemblage presenting a greater challenge to spatially separate catches. Yet we show how this information may be utilised by managers and fishers to better match catch to quota in highly mixed fisheries through changes in gear and locations fished.

### Spatial distributions indicate three species assemblages

**Figure 1:**
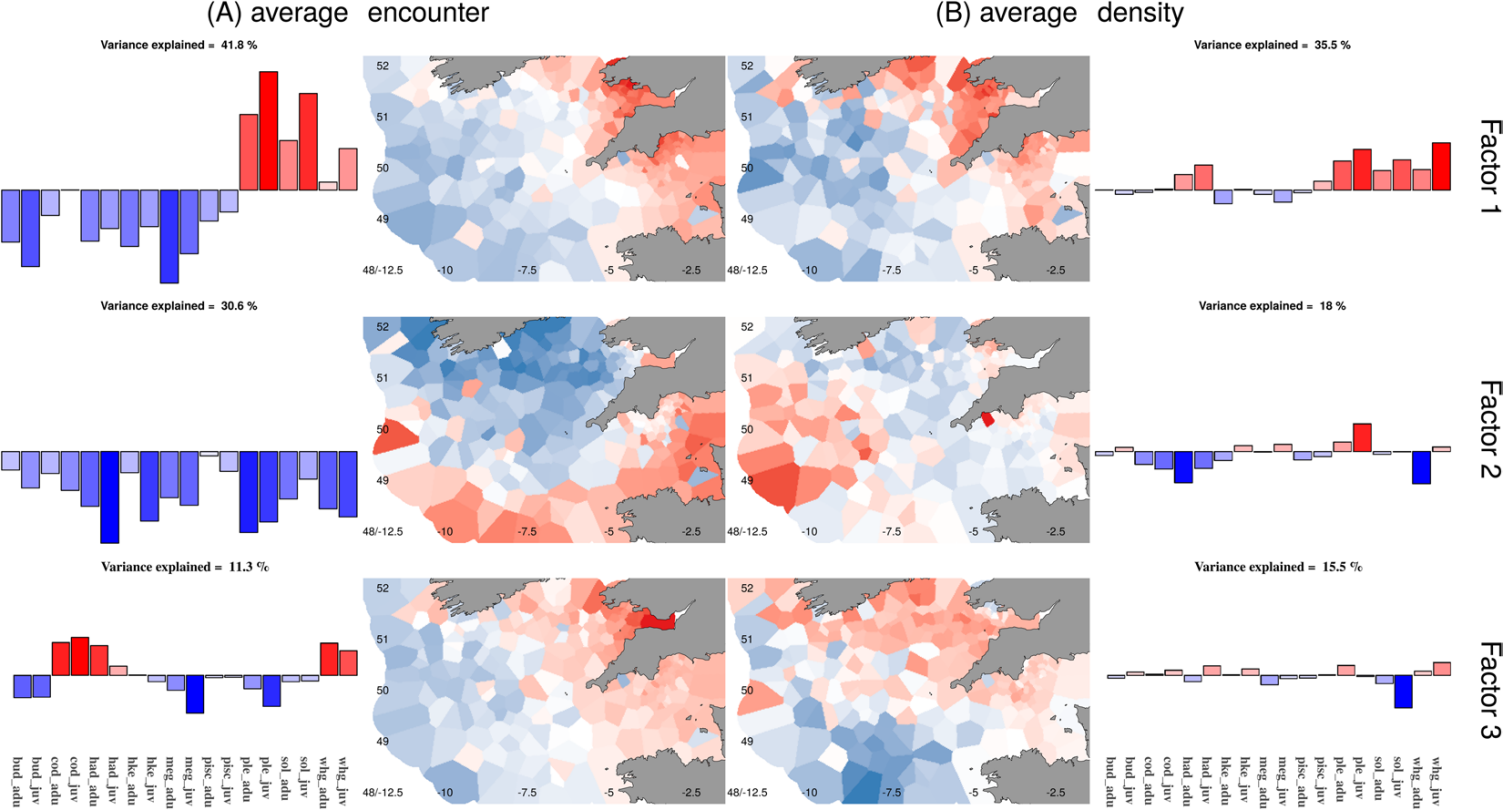
Factor values for the first three factors for (**A**) Average encounter probability and (**B**) Average positive density for the species (outer figures) and spatially (inner figures). Red: positive association to the factor, Blue: negative association.

A spatial dynamic factor analysis was used to decompose the dominant spatial patterns driving differences in average spatial variation. The first three factors (after PCA rotation) account for 83.7% of the between species variance in the probability of encountering a species (the “average encounter probability”) and 69% of the explained variance in catch rates on encounter (“average positive density”). A clear spatial pattern can been seen both for average encounter probability and average positive density, with a positive coefficient value associated with the first factor in the inshore north easterly part of the Celtic Sea into the Bristol Channel and Western English Channel, moving to a negative coefficient value offshore in the south-westerly waters (Fig. 1). The species loadings show plaice, sole and whiting to be positively associated with the first factor for average encounter probability while the other species are negatively associated. For average positive density, positive associations are also found for haddock and juvenile cod (weakly positive), indicative of a more inshore distribution for these species.

On the second spatial factor for average encounter probability a north/south split can be seen at approximately 49°N while positive density is more driven by a positive coefficient in the deeper westerly waters as well as some inshore areas. Species loadings for the second factor indicate there are positive associations for juvenile white-bellied anglerfish, juvenile hake, juvenile megrim, plaice and juvenile whiting with average positive density, which may reflect two different spatial distributions in the more offshore and in the inshore areas (Fig. 1).

On the third factor, there is a positive coefficient for the easterly waters for encounter probability and negative coefficient with the westerly waters. This splits the roundfish species (cod, haddock and whiting, that all have a positive association with the third factor for average encounter probability) from the rest of the species (that have a negative association). Positive density is driven by a north/south split (Fig. 1), with positive coefficient values in the northerly areas. Juvenile anglerfishes (white- and black- bellied), cod, juvenile haddock, hake, adult plaice and whiting are also positively associated with the third factor towards the north while adult anglerfishes, adult haddock, megrim, juvenile plaice and sole have negative loadings reflecting their more southerly distribution (Fig. 1).

While this exploratory factor analysis models unobserved drivers of distribution, we considered what might be driving the differences seen in the spatial factor coefficients and species loadings. The first factor was highly correlated with log(depth) for both average encounter probability coefficients (−0.85, CI = −0.88 to −0.81; Fig. S1) and average positive density coefficients (−0.71, CI = −0.77 to −0.65; Fig. S2). A random forest classification tree assigned 80% of the variance in the first factor for average encounter probability to depth and predominant substrate type, with the majority (86%) of the variance explained by depth. The variance explained by these variables dropped to 25% on the second factor with a more even split between depth and substrate, while explaining 60% of the variance on the third factor. For average positive density, the variables explained less of the variance with 62%, 35%, and 31% for each of the factors, respectively.

It is clear that depth and to a lesser extent substrate are important variables for describing the main driver of similarities and differences in distributions and abundances for the different species. The first factor correlates strongly with these variables, despite them not explicitly being incorporated in the model. While depth and substrate were incorporated as covariates in an alternative model formulation (see Methods), they were not found to improve predictions as the random fields adequately captured the influence of these variables on spatial variation in abundance. The utility of these variables as predictors of species distributions has been identified in other marine species distribution models^35^. The advantage to the approach taken here is that, where such data is unavailable at an appropriate spatial resolution, the spatial factor analysis can adequately characterised the species spatial dynamics.

### Species assemblages show similar spatiotemporal patterns

While there are clear spatial patterns in the factor coefficients describing differences in average encounter probability and positive density (Fig. 1), the interannual differences in factor coefficients show less structure (Figs S5 and S6). These interannual differences are important as they reflect the ability of fishers to predict where they can target or avoid species from one year to the next, without which it may be difficult to balance catches with available quota and avoid unwanted catch.

**Figure 2:**
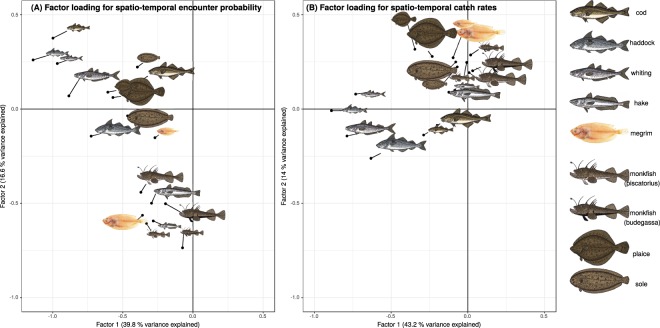
Position of each species on the first two axes from the factor analysis for (**A**) spatiotemporal encounter probability and (**B**) spatiotemporal positive density. Fish images from The Fisherman/Shutterstock.com and Richard Griffin/Shutterstock.com.

Spatiotemporal factor coefficients for encounter probability and positive density did not show the same spatial pattern driving species distributions from year to year, but when the first two factor loadings are plotted clear relationships in species association with spatiotemporal factor coefficients identify the three different assemblages (Fig. 2). The same factors appear to drive spatiotemporal (interannual changes in) distributions of megrim, anglerfish species and hake (the deeper water species, forming an assemblage negatively associated with the second axes of Fig. 2) and the roundfish and flatfish (two assemblages more positively associated with the second axes of Fig. 2A). For spatiotemporal positive density (Fig. 2B) cod, haddock and whiting (the roundfish species) are separated from plaice, sole (the flatfish) and the deeper water assemblage. As such, it can be predicted that higher catches of a species within a assemblage (e.g. cod in roundfish) would be expected when catching another species within that assemblage (e.g. whiting in roundfish). This suggests that one or more common environmental drivers are influencing the distributions of the assemblages, and that driver differentially affects the different assemblages. Temperature is often included as a covariate in species distribution models, but was found not to contribute to the variance in the first factor coefficients (Fig. S6, no correlations found for either spatiotemporal encounter probability or positive density) and so was not included as a covariate in the final model.

### Covariance in spatiotemporal abundance within species assemblages

**Figure 3:**
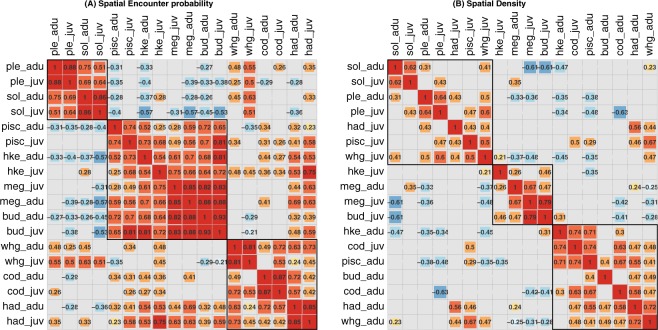
Inter-species correlations for (**A**) spatial encounter probability over all years and (**B**) spatial positive density. Species are clustered into three groups based on a hierarchical clustering method with non-significant correlations (the Confidence Interval [±1.96 * SEs] spanned zero) left blank.

To gain greater insight into the community dynamics we considered how species covary in space and time through correlations among species. Pearson correlation coefficients for the modelled average spatial encounter probability (Fig. 3A) show clear strong associations between adult and juvenile size classes for all species (>0.75 for all species except hake, 0.56). Among species, hierarchical clustering identified the same three common species-groups as our visual inspection of factor loadings above, with roundfish (cod, haddock, whiting) closely grouped, with correlations for adult cod with adult haddock and adult whiting of 0.73 and 0.5 respectively, while adult haddock with adult whiting was 0.63 (Fig. 3A). Flatfish (plaice and sole) are also strongly correlated with adult plaice and sole having a coefficient of 0.75. The final group are principally the species found in the deeper waters (hake, megrim and both anglerfish species) with megrim strongly associated with the black-bellied anglerfish species (0.88). Negative relationships were found between plaice and sole, and white-bellied anglerfish (−0.31 and −0.28 for the adult size class), black-bellied anglerfish (−0.27, −0.26 for the adult size class) and hake (−0.33, −0.37) (Fig. 3A) indicating spatial separation in distributions, with the flatfish found more inshore. This underscores the correlations among species seen in associations of each species with factors, with three distinct assemblages being confirmed.

Correlation coefficients for the average positive density (Fig. 3B) show fewer significant positive or negative relationships among species than for encounter probability, but still evident are the strong correlation among the roundfish with higher catches of cod correlated with higher catches of haddock (0.58) and whiting (0.47), as well as the two anglerfish species (0.71 for white-bellied and 0.44 for black-bellied) and hake (0.73). Similarly, plaice and sole are correlated (0.31) and higher catches of one would expect to see higher catches of the other, but also higher catches of some juvenile size classes of roundfish (whiting and haddock) and anglerfish species. Negative correlation of juvenile megrim, anglerfish (budegassa) and hake with adult sole (−0.61, −0.61 and −0.47 respectively), plaice (−0.36 and −0.35 for megrim and hake only) indicate high catches of one can predict low catches of the other successfully.

To understand how stable relationships between catches of pairs of species were from one year to the next, we regressed the correlation coefficients for the average spatial correlations between pairs for species *x* and species *y* across all years with those of the spatiotemporal population correlations, representing how correlations between species *x* and species *y* change from year to year (Fig. S9). The correlations were 0.60 (0.52–0.66) and 0.47 (0.38–0.55) for encounter probability and positive density respectively (Fig. S9a,b). These indicate generally predictable relationships between species from one year to the next and suggests that a positive or negative correlation between two species is likely to persist from one year to the next, and that species are consistently correlated in hauls. However, the regressions between the spatial correlations and the spatiotemporal correlations shows high variance (R^2^ = 0.36 and 0.22 respectively), indicating that the scale of these relationships do change from one year to the next. This unpredictability would have implications for the fishery if, for example, catches of an unwanted species increased when caught with a target species above a level expected in the fishery potentially leading to challenges for fishers when trying to balance catch with quotas in mixed fisheries. It can be seen in the spatial factor maps that there are subtle differences in patterns in spatial factor coefficients from one year to the next (Figs S4 and S5), indicating changes may be driven by temporally changing environmental factors and species behaviour.

### Potential to separate catches within assemblages under the landing obligation

**Figure 4:**
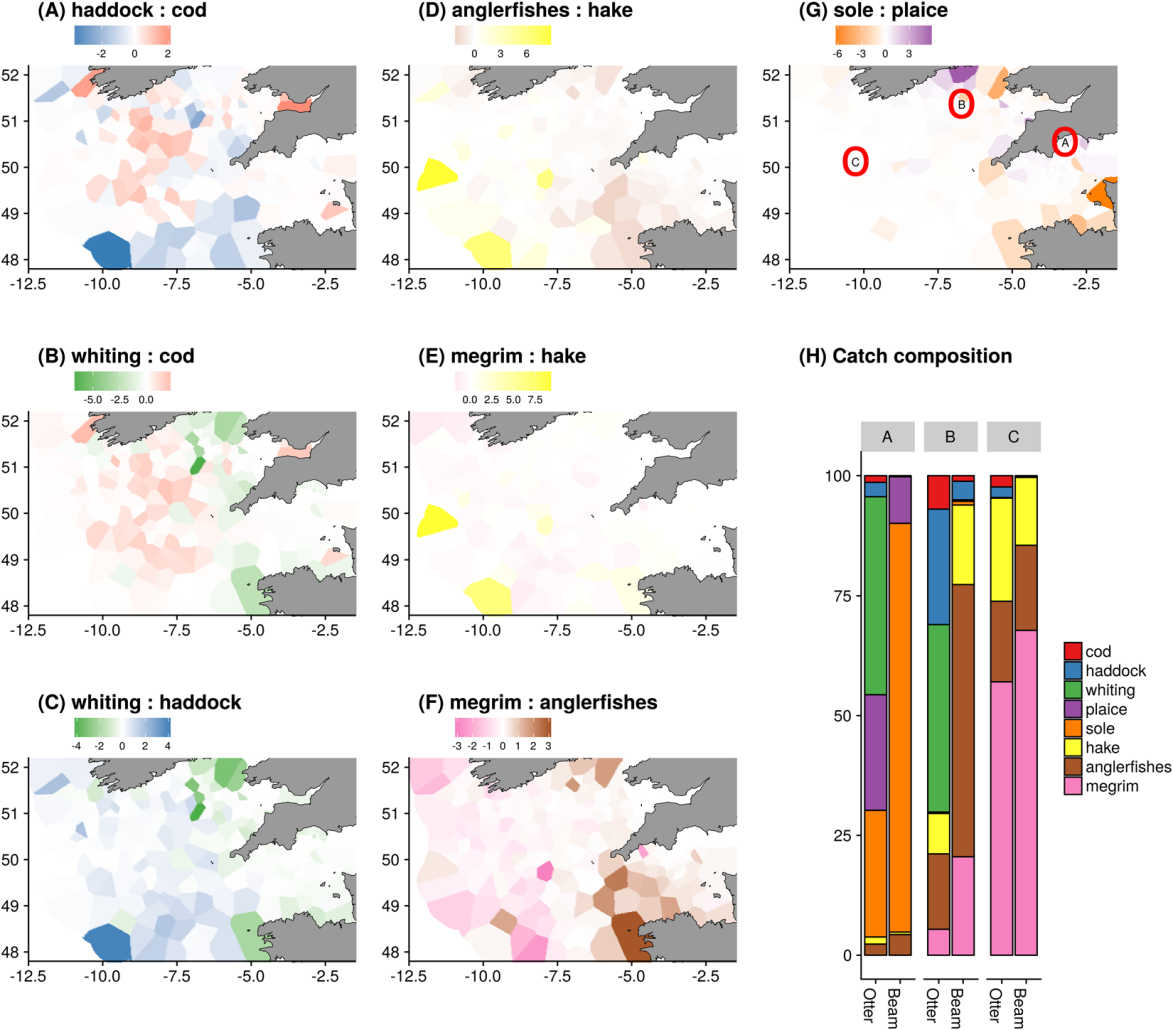
Differences in the standardised spatial density for pairs of species and expected catch rates for two different gears at three different locations in 2015. A, B and C in sub-figure (**H**) correspond to the spatial locations illustrated in sub-figure (**G**).

The analysis shows the interdependence within three assemblages of roundfish, flatfish and deeper water species, where catching one species within the group indicates a high probability of catching the other species. This has important implications for how spatial avoidance can be used to support implementation of the EU’s landing obligation. If production from mixed fisheries is to be maximised, decoupling catches of species between and within the groups will be key. For example, asking where the maximal separation in the densities of two coupled species is likely to occur? To address this requirement, we map the difference in spatial distribution within a species-group for each pair of species for a single year (2015; Fig. 4).

Cod had a more north-westerly distribution than haddock and a more westerly distributed than whiting roughly delineated by the 7°W line (Fig. 4A). Whiting appeared particularly concentrated in an area between 51 and 52°N and 5 and 7°W, which can be seen by comparing the whiting distribution with both cod (Fig. 4B) and haddock (Fig. 4C). For the deeper water species hake are more densely distributed in two locations around 10 W and 48 N and 12 W and 50 N compared to the anglerfish species (anglerfishes have been presented together as they are jointly managed under a single quota) and megrim, which were more widely spatially distributed (Fig. 4D,E). Megrim has a fairly even density across the modelled area as indicated by the large amount of white space in Fig. 4E. For anglerfishes and megrim (Fig. 4F), anglerfishes have a more easterly distribution than megrim. For the flatfish species plaice and sole (Fig. 4G), plaice appear to be more densely distributed along the coastal areas of Ireland and Britain, while sole are more densely distributed in the Southern part of the English Channel along the coast of France.

Predicted catch distribution from a “typical” otter trawl gear and beam trawl fishing at three different locations highlights the differences fishing gear makes on catches (Fig. 4H). Both gear selectivity and location fished have important effects on the catch composition; in the inshore area (location “A”) plaice and sole are the two main species in the catch reflecting their distribution and abundance, though the otter trawl gear catches a greater proportion of plaice to sole than the beam trawl. The area between Britain and Ireland (location “B”) has a greater contribution of whiting, haddock, cod, hake and anglerfishes in the catch with the otter trawl catching a greater proportion of the roundfish, haddock, whiting and cod while the beam trawl catches more anglerfishes and megrim. The offshore area has a higher contribution of megrim, anglerfishes and hake with the otter trawl catching a greater share of hake and the beam trawl a greater proportion of megrim. Megrim dominates the catch for both gears in location “C”, reflecting its relative abundance in the area irrespective of the gear deployed.

## Discussion

Our study is framed by the problem of addressing the scientific challenges of implementing the landing obligation for mixed fisheries. In application to the Celtic Sea, we have identified spatial separation of three distinct assemblages (roundfish, flatfish and deeper water species) while showing that only subtle differences exist in distributions within assemblages. The differences in catch compositions between gears at the same location (Fig. 4H) show that changing fishing methods affects catch, yet that differences in catches between locations are likely to be more important. For example, beam trawls fishing at the inshore locations (e.g. location “A” in Fig. 4) are likely to predominately catch plaice and sole, yet switching to the offshore locations (e.g. location “C”) would likely yield greater catches of megrim and anglerfishes. Such changes in spatial fishing patterns are likely to play an important role in supporting implementation of the landing obligation.

More challenging is within-group spatial separation due to significant overlap in spatial distributions for the species, driven by common environmental factors. Subtle changes may yield some benefit in changing catch composition, yet the outcome is likely to be much more difficult to predict. For example, subtle differences in the distribution of cod, haddock and whiting can be seen in Fig. 4A–C, showing spatial separation of catches is much more challenging and likely to require support from other measures such as changes to the selectivity characteristics of gear^36^. For example we identified a spatial overlap of flatfish with juvenile roundfish in our species correlations (Fig. 3); reducing catches of incidental bycatch on the main target fishing grounds will likely require adaptations to fishing gear to address bycatch without significant economic impacts on the fishery.

A role that science could play in supporting effectiveness of spatiotemporal avoidance would be to provide probabilistic advice on hotspots for species occurrence and high species density, which can inform fishing decisions. Previous modelling studies have shown how spatiotemporal models could improve predictions of high ratios of bycatch to target species^37–39^, and geostatistical models are well suited to this as they incorporate spatial dependency while providing for probabilities to be drawn from posterior distributions of the parameter estimates. We posit that such advice on “hot spots” as a supportive measure to incentivise avoidance of areas of high bycatch risk could be enhanced by integrating data obtained directly from commercial fishing vessels rapidly while modelling densities at small time scales (e.g., weekly). Short-term forecasts of distribution could inform fishing choices while also capturing seasonal differences in distributions, akin to weather forecasting. Advice informed by a model including a seasonal or real-time component could inform optimal policies for time-area closures, move-on rules or even as informal information to be utilised by fishers directly without the need for costly continuous data collection on environmental parameters, but by using the “vessels-as-laboratories” approach.

An important question for the implementation of the EU’s landing obligation is how far spatial avoidance can go to achieving catch balancing in fisheries. Our model captures differences between location fished for two gear types and their broad scale effect on catch composition, information crucial for managers in implementing the landing obligation. It is likely, however, that this analysis reflects a lower bound on the utility of spatial avoidance as fine-scale behavioural decisions such as time-of-day, gear configuration and location choices can also be used to affect catch^40,41^. Results of empirical studies undertaken elsewhere^5,6^ suggest limits to the effectiveness of spatial avoidance *in situ*. For example, differences in ability to change catch composition have been observed for different fleets; in the North Sea targeting ability was found to differ between otter and beam trawlers as well as between vessels of different sizes^42^. The particular socioeconomic circumstances for individual vessels is therefore important to take account when considering the effectiveness of spatial targetting and avoidance.

Under the landing obligation the balance of risk-reward for trip level fishing decisions about where to fish may change. For example, are fishers likely to fish in “safe” areas where its known there are lower catches of the target species but also decreased risk of encountering bycatch? How do decisions about level of risk affect the likelihood of overshooting available quota and potential profit and losses for individual trips? Set in this context, the parameter estimates could be used to simulate from a distribution of catches in the fishery at different locations and therefore inform on the possibility of extreme catch events and potential consequences for overshooting quotas. Alternatively, where fisheries data is available with factors such as weather, quota uptake and previous catches these could be included as covariates in the model to help identify causes for high bycatch events. This information may be of interest in identifying optimum strategies, or used in future work to model closure risks for fisheries operating in different locations and conditions given quota constraints. Such analyses on risk and decision making are likely to hinge on micro-level decisions by fishers and would be a useful compliment to broader scale considerations such as those detailed here.

Our framework allows for a quantitative understanding of the broad scale global production set available to fishers^43^ and thus the extent to which they can alter catch compositions while operating in a mixed fishery. Simulations of spatial effort allocation scenarios based on the production sets derived from the model estimates could be used as inputs to fisher behavioural models to allow for the identification of lower bounds of optimum spatial harvest strategies. Modelling different spatial strategies at the individual or fishery level would provide managers with an information base to examine trade-offs in quota setting, thus providing a scientific basis to assessing the ability of technical measures to meet the goal of maximising catches in mixed fisheries within single stock quota constraints^7^. Additionally, the correlations among species could provide information on fisheries at risk of capturing protected, endangered or threatened species such as elasmobranchs, and allow identification of areas where there are high ratios of protected to target species.

Complex environmental, fishery and community drivers of distribution for groups of species highlights the scale of the challenge in separating catches within the assemblages using spatial management measures. This has important implications for management of mixed fisheries under the EU landing obligation. Our analysis identifies where it may be easier to separate catches of species (among groups) and where it is more challenging (within groups). We propose that the dimension-reduction framework presented in Figs 1–4 provides a viable route to reducing the complexity of highly mixed fisheries. This can allow informed management discussion over more traditional anecdotal knowledge of single-species distribution in space and time.

## Methods

### Model structure

VAST (software in the R statistical programming language can be found here: www.github.com/james-thorson/VAST) implements a delta-generalised linear mixed modelling (GLMM) framework that takes account of spatiotemporal correlations among species through implementation of a spatial dynamic factor analysis (SDFA). Spatial variation is captured through a Gaussian Markov Random Field, while we model random variation among species and years. Covariates affecting catchability (to account for differences between fishing surveys) and density (to account for environmental preferences) can be incorporated for predictions of presence and positive density. The following briefly summarises the key methods implemented in the VAST framework. For full details see Thorson *et al*.^44^.

#### SDFA

A spatial dynamic factor analysis incorporates advances in joint dynamic species distribution models^44^ to take account of associations among species by modelling response variables as a multivariate process. This is achieved through implementing a factor analysis decomposition where common latent trends are estimated so that the number of common trends is less than the number of species modelled. The factor coefficients are then associated through loadings for each factor that return a positive or negative association of one or more species with any location. Log-density of any species is then be described as a linear combination of factors and loadings:

1 $${\theta }_{c}(s,t)=\mathop{\sum }\limits_{j=1}^{{n}_{j}}\,{L}_{c,j}{\psi }_{j}(s,t)+\mathop{\sum }\limits_{k=1}^{{n}_{k}}\,{\gamma }_{k,c}{\chi }_{k}(s,t)$$

where *θ*_*c*_(*s*, *t*) represents log-density for species *c* at site *s* at time *t*, *ψ*_*j*_ is the coefficient for factor *j*, *L*_*c*,*j*_ the loading matrix representing association of species *c* with factor *j* and *γ*_*k*,*c*_*χ*_*k*_(*s*, *t*) the linear effect of covariates at each site and time^45^.

The factor analysis can summarize community dynamics and identify which species and life-stages have similar spatiotemporal patterns. This allows inference regarding species distributions and abundance of poorly sampled species through association with other species, and also provides estimates of spatiotemporal correlations among species^45^.

#### Estimation of abundances

Spatiotemporal encounter probability and positive catch rates are modelled separately with spatiotemporal encounter probability modelled using a logit-link linear predictor;

2 $$\begin{array}{rcl}logit[p({s}_{i},{c}_{i},{t}_{i})] & = & {\beta }_{p}({c}_{i},{t}_{i})+\mathop{\sum }\limits_{f=1}^{{n}_{\omega }}\,{L}_{\omega }({c}_{i},f){\omega }_{p}({s}_{i},f)+\mathop{\sum }\limits_{f=1}^{{n}_{\varepsilon }}\,{L}_{\varepsilon }({c}_{i},f){\varepsilon }_{p}({s}_{i},f,{t}_{i})\\  &  & +\,\mathop{\sum }\limits_{v=1}^{{n}_{v}}\,{\delta }_{p}(v){Q}_{p}({c}_{i},{v}_{i})\end{array}$$

and positive catch rates modelling using a gamma- distribution^30^.

3 $$\begin{array}{rcl}log[r({s}_{i},{c}_{i},{t}_{i})] & = & {\beta }_{r}({c}_{i},{t}_{i})+\mathop{\sum }\limits_{f=1}^{{n}_{\omega }}\,{L}_{\omega }({c}_{i},f){\omega }_{r}({s}_{i},f)+\mathop{\sum }\limits_{f=1}^{{n}_{\varepsilon }}\,{L}_{\varepsilon }({c}_{i},f){\varepsilon }_{r}({s}_{i},f,{t}_{i})\\  &  & +\,\mathop{\sum }\limits_{v=1}^{{n}_{v}}\,{\delta }_{r}(v){Q}_{r}({c}_{i},{v}_{i})\end{array}$$

where *p*(*s*_*i*_, *c*_*i*_, *t*_*i*_) is the predictor for encounter probability for observation *i*, at location *s* for species *c* and time *t* and *r*(*s*_*i*_, *c*_*i*_, *t*_*i*_) is similarly the predictor for the positive density. β_*_(*c*_*i*_, *t*_*i*_) is the intercept, ω_*_(*s*_*i*_, *c*_*i*_) the spatial variation at location *s* for factor *f*, with *L*_*ω*_(*c*_*i*_, *f*) the loading matrix for spatial covariation among species. ε_*_(*s*_*i*_, *c*_*i*_, *t*_*i*_) is the linear predictor for spatiotemporal variation, with *L*_*ε*_(*c*_*i*_, *f*) the loading matrix for spatiotemporal covariance among species and δ_*_(*c*_*i*_, *v*_*i*_) the contribution of catchability covariates for the linear predictor with ${Q}_{{c}_{i},{v}_{i}}$ the catchability covariates for species *c* and vessel *v*; * can be either *p* for probability of encounter or *r* for positive density.

The Delta-Gamma formulation is then:

4 $$\begin{array}{rcl}Pr(C=\mathrm{0)} & = & 1-p\\ Pr(C=c|c > \mathrm{0)} & = & p\cdot \frac{{\lambda }^{k}{c}^{k-1}\cdot exp(\,-\,\lambda c)}{{\Gamma }_{k}}\end{array}$$

for the probability *p* of a non-zero catch *C* given a gamma distribution for for the positive catch with a rate parameter *λ* and shape parameter *k*.

#### Spatiotemporal variation

The spatiotemporal variation is modelled using Gaussian Markov Random Fields (GMRF) where observations are correlated in space through a Matérn covariance function with the parameters estimated within the model. Here, the correlation decays smoothly over space the further from the location and includes geometric anisotropy to reflect that correlations may decline in one direction faster than another (e.g. moving offshore)^31^. The best fit estimated an anisotropic covariance where the correlations were stronger in a north-east - south-west direction, extending approximately 97 km and 140 km before correlations for encounter probability and positive density reduced to <10%, respectively (Fig. S10). Incorporating the spatiotemporal correlations among species provides more efficient use of the data as inference can be made about poorly sampled locations from the covariance structure.

A probability distribution for spatiotemporal variation in both encounter probability and positive catch rate was specified, ε_*_(*s*, *p*, *t*), with a three-dimensional multivariate normal distribution so that:

5 $$vec[{{\bf{E}}}_{\ast }(t)]\sim MVN(0,{{\bf{R}}}_{\ast }\otimes {{\bf{V}}}_{\varepsilon \ast })$$

Here, *vec*[E_*_(*t*)] is the stacked columns of the matrices describing $\varepsilon \ast (s,p,t)$ at every location, species and time, R_*_ is a correlation matrix for encounter probability or positive catch rates among locations and V_*_ a covariance matrix for encounter probability or positive catch rate among species (modelled within the factor analysis). $\otimes$ represents the Kronecker product so that the correlation among any location and species can be computed^44^.

#### Incorporating covariates

Survey catchability (the relative efficiency of a gear catching a species) was estimated as a fixed effect in the model, *δ*_*s*_(*v*), to account for differences in spatial fishing patterns and gear characteristics, which affect encounter and capture probability of the sampling gear^46^. Parameter estimates (Fig. S11) showed clear differential effects of surveys using otter trawl gears (more effective for round fish species) and beam trawl gears (more effective for flatfish species).

No fixed covariates for habitat quality or other predictors of encounter probability or positive density were included. While incorporation may improve the spatial predictive performance^44^, it was not found to be the case here based on model selection with Akaike Information Criterion (AIC) and Bayesian Information Criterion (BIC).

#### Parameter estimation

Parameter estimation was undertaken through Laplace approximation of the marginal likelihood for fixed effects while integrating the joint likelihood (which includes the probability of the random effects) with respect to random effects. This was implemented using Template Model Builder (TMB^47^) with computation supported by use of the Irish Centre for High End Computing (ICHEC; http://www.ichec.ie) facility.

### Data

The model integrates data from seven fisheries-independent surveys taking account of correlations among species spatiotemporal distributions and abundances to predict spatial density estimates consistent with the resolution of the data.

The model was fitted to nine species separated into adult and juvenile size classes (Table S2) to seven survey series (Table S1) in the Celtic Sea bounded by 48°N to 52°N latitude and 12°W to 2°W longitude (Fig. S8) for the years 1990–2015 inclusive.

The following steps were undertaken for data processing: (i) data for survey stations and catches were downloaded from ICES Datras (www.ices.dk/marine-data/data-portals/Pages/DATRAS.aspx) or obtained directly from the Cefas Fishing Survey System (FSS); (ii) data were checked and any tows with missing or erroneously recorded station information (e.g. tow duration or distance infeasible) removed; (iii) swept area for each of the survey tows was estimated based on fitting a GAM to gear variables so that Doorspread = s(Depth) + DoorWt + WarpLength + WarpDiameter + SweepLength and a gear specific correction factor taken from the literature^48^; (iii) fish lengths were converted to biomass (Kg) through estimating a von bertalanffy length weight relationship, $Wt=a\cdot {L}^{b}$, fit to sampled length and weight of fish obtained in the EVHOE survey and aggregated within size classes (adult and juvenile). Details on the downloading and processing of the data are available in Rmarkdown format (code and steps combined) as supplementary material.

The final dataset comprised of estimates of catches (including zeros) for each station and species and estimated swept area for the tow.

### Model setup

The spatial domain was set up to include 250 knots representing the Gaussian Random Fields. The model was configured to estimate nine factors each to describe the spatial and spatiotemporal encounter probability and positive density parameters, with a logit-link for the linear predictor for encounter probability and log-link for the linear predictor for positive density, with an assumed gamma distribution.

Three candidate models were identified, (i) a base model where the vessel interaction was a random effect, (ii) the base but where the vessel x species effect was estimated as a fixed covariate, (iii) with vessel × species effect estimated, but with the addition of estimating fixed density covariates for both predominant habitat type at a knot and depth. AIC and BIC model selection favoured the second model (Table S3). The final model included estimating 1,674 fixed parameters and predicting 129,276 random effect values.

### Model validation

Q-Q plots show good fit between the derived estimates and the data for positive catch rates and between the predicted and observed encounter probability (S12, S13). Further, model outputs are consistent with stock-level trends abundances over time from international assessments (S14), yet also provide detailed insight into species co-occurrence and the strength of associations in space and time.

## Electronic supplementary material

Processing and exploration for Celtic Sea fishery-independent trawl survey data

Supplementary Tables and Figures

## Data Availability

Data used to fit the model is available via the ICES Datras data portal (http://www.ices.dk/marine-data/data-portals/Pages/DATRAS.aspx) for two surveys and on request to the corresponding author for the remaining five surveys.

## References

[1] Worm B (2009). Rebuilding Global. Fisheries. Science.

[2] FAO. The state of world fisheries and aquaculture. *Food and Agriculture Oraganization of the United Nations*,** 218**, 92-5-105177-1, 978-92-5-106675-1 (2014).

[3] Béné C (2016). Contribution of Fisheries and Aquaculture to Food Security and Poverty Reduction: Assessing the Current Evidence. World Development.

[4] Mcclanahan T, Allison EH, Cinner JE (2015). Managing fisheries for human and food security. Fish and Fisheries.

[5] Branch, T. & Hilborn, R. Matching catches to quotas in a multispecies trawl fishery: targeting and avoidance behavior under individual transferable quotas. *Canadian Journal of Fisheries and Aquatic Sciences* **65**, 1435–1446, http://article.pubs.nrc-cnrc.gc.ca/ppv/RPViewDoc?issn=1205-7533{&}volume=65{&}issue=7{&}startPage=1435{&}ab=y, 10.1139/F08-065 (2008).

[6] Kuriyama PT, Branch TA, Bellman MA, Rutherford K (2016). Catch shares have not led to catch-quota balancing in two North American multispecies trawl fisheries. Marine Policy.

[7] Ulrich C (2017). Achieving maximum sustainable yield in mixed fisheries: A management approach for the North Sea demersal fisheries. ICES Journal of Marine Science.

[8] Batsleer J, Hamon KG, Overzee HMJ, Rijnsdorp AD, Poos JJ (2015). High-grading and over-quota discarding in mixed fisheries. Reviews in Fish Biology and Fisheries.

[9] Borges, L. The evolution of a discard policy in Europe. *Fish and Fisheries* 534–540, 10.1111/faf.12062 (2015).

[10] Uhlmann SS (2014). Discarded fish in European waters: General patterns and contrasts. ICES Journal of Marine Science.

[11] European Commission. Regulation (EU) No. 1380/2013 of the European Parliament and of the Council of 11 December 2013 on the Common Fisheries Policy, amending Council Regulations (EC) No. 1954/2003 and (EC) No. 1224/2009 and repealing Council Regulations (EC) No 2371/2002 and (EC) (2013).

[12] European Parliament. Directive 2009/28/EC of the European Parliament and of the Council of 23 April 2009. *Official Journal of the European Union* **140**, 16–62, 10.3000/17252555.L_2009.140.eng, 534 (2009).

[13] Garcia, S. M., Zerbi, A., C, A., Do Chi, T. & Lasserre, G. The ecosystem approach to fisheries. *FAO Fisheries Technical Paper* **443**, 71, http://www.fao.org/docrep/006/Y4773E/y4773e05.html, 10.1079/9781845934149.0000 (2003).

[14] Holland DS (2008). Are Fishermen Rational? A Fishing Expedition. Marine Resource Economics.

[15] Hoff A (2010). Economic effort management in multispecies fisheries: The FcubEcon model. ICES Journal of Marine Science.

[16] Condie, H. M., Grant, A. & Catchpole, T. L. Incentivising selective fishing under a policy to ban discards; lessons from European and global fisheries. *Marine Policy* **45**, 287–292, https://linkinghub.elsevier.com/retrieve/pii/S0308597X1300198X, 10.1016/j.marpol.2013.09.001 (2014).

[17] Condie, H. M., Grant, A. & Catchpole, T. L. Does banning discards in an otter trawler fishery create incentives for more selective fishing? *Fisheries Research* **148**, 137–146, https://linkinghub.elsevier.com/retrieve/pii/S016578361300221X, 10.1016/j.fishres.2013.09.011 (2013).

[18] Fulton EA, Smith AD, Smith DC, Van Putten IE (2011). Human behaviour: The key source of uncertainty in fisheries management. Fish and Fisheries.

[19] Van Putten IE (2012). Theories and behavioural drivers underlying fleet dynamics models. Fish and Fisheries.

[20] Fraser HM, Greenstreet SPR, Fryer RJ, Piet GJ (2008). Mapping spatial variation in demersal fish species diversity and composition in the North Sea: Accounting for species- and size-related catchability in survey trawls. ICES Journal of Marine Science.

[21] Gerritsen HD, Lordan C, Minto C, Kraak SBM (2012). Spatial patterns in the retained catch composition of Irish demersal otter trawlers: High-resolution fisheries data as a management tool. Fisheries Research.

[22] Needle CL, Catarino R (2011). Evaluating the effect of real-time closures on cod targeting. ICES Journal of Marine Science.

[23] Holmes SJ (2011). Using fishery-dependent data to inform the development and operation of a co-management initiative to reduce cod mortality and cut discards. ICES Journal of Marine Science.

[24] Beare, D. J. *et al*. Study for the Revision of the plaice box - a Final Report. *Tech. Rep* (2010).

[25] Dinmore TA, Duplisea DE, Rackham BD, Maxwell DL, Jennings S (2003). Impact of a large-scale area closure on patterns of fishing disturbance and the consequences for benthic communities. ICES Journal of Marine Science.

[26] Gardner B, Sullivan PJ, Morreale SJ, Epperly SP (2008). Spatial and temporal statistical analysis of bycatch data: patterns of sea turtle bycatch in the North Atlantic. Canadian Journal of Fisheries and Aquatic Sciences.

[27] Dunn DC, Boustany AM, Halpin PN (2011). Spatio-temporal management of fisheries to reduce by-catch and increase fishing selectivity. Fish and Fisheries.

[28] Dunn DC (2014). Empirical move-on rules to inform fishing strategies: A New England case study. Fish and Fisheries.

[29] Thorson JT (2015). Spatial factor analysis: A new tool for estimating joint species distributions and correlations in species range. Methods in Ecology and Evolution.

[30] Thorson JT, Shelton AO, Ward EJ, Skaug HJ (2015). Geostatistical delta-generalized linear mixed models improve precision for estimated abundance indices for West Coast groundfishes. ICES Journal of Marine Science.

[31] Thorson JT, Ward EJ (2013). Accounting for space-time interactions in index standardization models. Fisheries Research.

[32] Ellis, J. R., Rogers, S. I. & Freeman, S. M. Demersal Assemblages in the Irish Sea, St George’s Channel and Bristol Channel. *Estuarine*, *Coastal and Shelf Science* **51**, 299–315, https://www.sciencedirect.com/science/article/pii/S0272771400906772, 10.1006/ecss.2000.0677 (2000).

[33] Mateo, M., Pawlowski, L. & Robert, M. Highly mixed fisheries: fine-scale spatial patterns in retained catches of French fisheries in the Celtic Sea. *ICES Journal of Marine Science: Journal du Conseil* fsw129, 10.1093/icesjms/fsw129 (2016).

[34] STECF. EU’s Scientific, Technical and Economic Committee on Fisheries (STECF): Fisheries Dependent Information Database, https://stecf.jrc.ec.europa.eu/dd/effort/graphs-annex (2017).

[35] Robinson LM (2011). Pushing the limits in marine species distribution modelling: Lessons from the land present challenges and opportunities. Global Ecology and Biogeography.

[36] Santos J (2016). Reducing flatfish bycatch in roundfish fisheries. Fisheries Research.

[37] Ward EJ (2015). Using spatiotemporal species distribution models to identify temporally evolving hotspots of species co-occurrence. Ecological Applications.

[38] Cosandey-Godin A, Krainski ET, Worm B, Flemming JM (2015). Applying Bayesian spatiotemporal models to fisheries bycatch in the Canadian Arctic. Canadian Journal of Fisheries and Aquatic Sciences.

[39] Breivik ON, Storvik G, Nedreaas K (2016). Latent Gaussian models to decide on spatial closures for bycatch management in the Barents Sea shrimp fishery. Canadian Journal of Fisheries and Aquatic Sciences.

[40] Abbott JK, Haynie AC, Reimer MN (2015). Hidden Flexibility: Institutions, Incentives, and the Margins of Selectivity in Fishing. Land Economics.

[41] Thorson JT, Kristensen K (2016). Implementing a generic method for bias correction in statistical models using random effects, with spatial and population dynamics examples. Fisheries Research.

[42] Pascoe, S., Koundouri, P. & Bjørndal, T. Estimating targeting ability in multi-species fisheries: a primal multi-output distance function approach. *Land Economics*, 10.3368/le.83.3.382 (2007).

[43] Reimer MN, Abbott JK, Wilen JE (2017). Fisheries Production: Management Institutions, Spatial Choice, and the Quest for Policy Invariance. Marine Resource Economics.

[44] Thorson, J. T. & Barnett, L. A. K. Comparing estimates of abundance trends and distribution shifts using single- and multispecies models of fishes and biogenic habitat. *ICES Journal of Marine Science: Journal du Conseil* fsw193, 10.1093/icesjms/fsw193 (2017).

[45] Thorson JT (2016). Joint dynamic species distribution models: a tool for community ordination and spatio-temporal monitoring. Global Ecology and Biogeography.

[46] Thorson JT (2015). The importance of spatial models for estimating the strength of density dependence. Ecology.

[47] Kristensen, K., Nielsen, A., Berg, C. W., Skaug, H. & Bell, B. TMB: Automatic Differentiation and Laplace Approximation. *Journal of Statistical Software* **70**, 1–21, https://arxiv.org/abs/1509.00660, 10.18637/jss.v070.i05, 1509.00660 (2016).

[48] Piet GJ, Van Hal R, Greenstreet SPR (2009). Modelling the direct impact of bottom trawling on the North Sea fish community to derive estimates of fishing mortality for non-target fish species. ICES Journal of Marine Science.

