# Author Correction: Spatial separation of catches in highly mixed fisheries

**DOI:** 10.1038/s41598-020-60583-5

**Published:** 2020-03-11

**Authors:** Paul J. Dolder, James T. Thorson, Cóilín Minto

**Affiliations:** 1 Marine and Freshwater Research Centre, Galway-Mayo Institute of Technology (GMIT), Dublin Road, Galway, H91 T8NW Ireland; 2 Centre for Environment, Fisheries and Aquaculture Science (Cefas), Pakefield Road, Lowestoft, Suffolk, NR33 0HT United Kingdom; 3 Fisheries Resource Analysis and Monitoring Division, Northwest Fisheries Science Center, National Marine Fisheries Service, NOAA, 2725 Montlake Blvd E, Seattle, Washington, 98112 USA

Correction to: *Scientific Reports* 10.1038/s41598-018-31881-w, published online 17 September 2018

A supplementary file containing Figures S1 to S13 and Tables S1- S3 was omitted from the original version of this Article. This has been corrected in the HTML version of the Article; the PDF version was correct at time of publication.

