# Supplementary material for: Spatial separation of catches in highly mixed fisheries
Source: Sci Rep. 2018 Sep 17;8:13886. doi: 10.1038/s41598-018-31881-w (PMC6141535; doi:10.1038/s41598-018-31881-w)
Supplement: Supplementary file 1 — Processing and exploration for Celtic Sea fishery-independent trawl survey data [file 41598_2018_31881_MOESM1_ESM.pdf]

Processing and exploration for Celtic Sea fishery-independent trawl survey data

Paul J Dolder

May 4, 2017

This document is to detail the processing steps and workng up of data for fitting a geostatistical model (VAST; see https://github.com/james-thorson/VAST for detail) to trawl survey data covering the Celtic Sea.

The following data sources were used:

- ICES Datras (http://www.ices.dk/marine-data/data-portals/Pages/DATRAS.aspx exhchange data of Ifremer (France) EVHOE and Marine Institute (Ireland) IGFS fisheries-independent survey locations and catch records.
- Cefas (UK) collection of trawl survey locations and catch records.
- ICES Datras data product on estimated weights of fish at various lengths from the EVHOE survey series.

1 Length-weight conversion factors

As the survey records consist of count of fish at each length class and we are interested in working with biomass (weight) of fish, we first estimate a length-weight relationship for the different species from the Datras data product of weight at length estimates. The data is baseds on the EVHOE survey series only, due to availability within Datras.

A standard von bertalanffy length weight relationship was used, with two parameters to estimate:

$$Wt = a \cdot L^b \tag{1}$$

The raw data looks as follows for cod, megrim, anglerfishes, haddock, whiting, hake, plaice and sole:

```r
# Read data and remove records without corresponding weight
DF <- read.csv(file.path("DATRAS", "SMALK_EVHOE.csv"))  # read data
DF <- DF[!is.na(DF$IndWgt), ]

# Subset to species of interest sort(unique(DF$Species))
spp <- c("Gadus morhua", "Lepidorhombus whiffiagonis", "Lophius piscatorius",
    "Lophius budegassa", "Merlangius merlangus", "Melanogrammus aeglefinus",
    "Merluccius merluccius", "Pleuronectes platessa", "Solea solea")

## N.B. The length-weight relationship for anglerfishes
## doesn't hold, so we might need an alternative
## solution....'Pollachius pollachius' - no juveniles??

DF <- DF[DF$Species %in% spp, ]

# Plot
ggplot(DF, aes(x = LngtClass, y = IndWgt)) + geom_point(aes(colour = factor(Year))) +
    facet_wrap(~Species, scale = "free") + theme_bw()
```

To simplify the fitting procedure, the von bertalanffy relationship in equation 1 was rearranged to be linear on a log scale:

$$log(Wt) = log(a) + b \cdot log(L) + \varepsilon \tag{2}$$

```r
DF$lWt <- log(DF$IndWgt)
DF$lL <- log(DF$LngtClass)

ggplot(DF, aes(x = lL, y = lWt)) + geom_point(aes(colour = factor(Year))) +
    facet_wrap(~Species, scale = "free") + theme_bw()
```

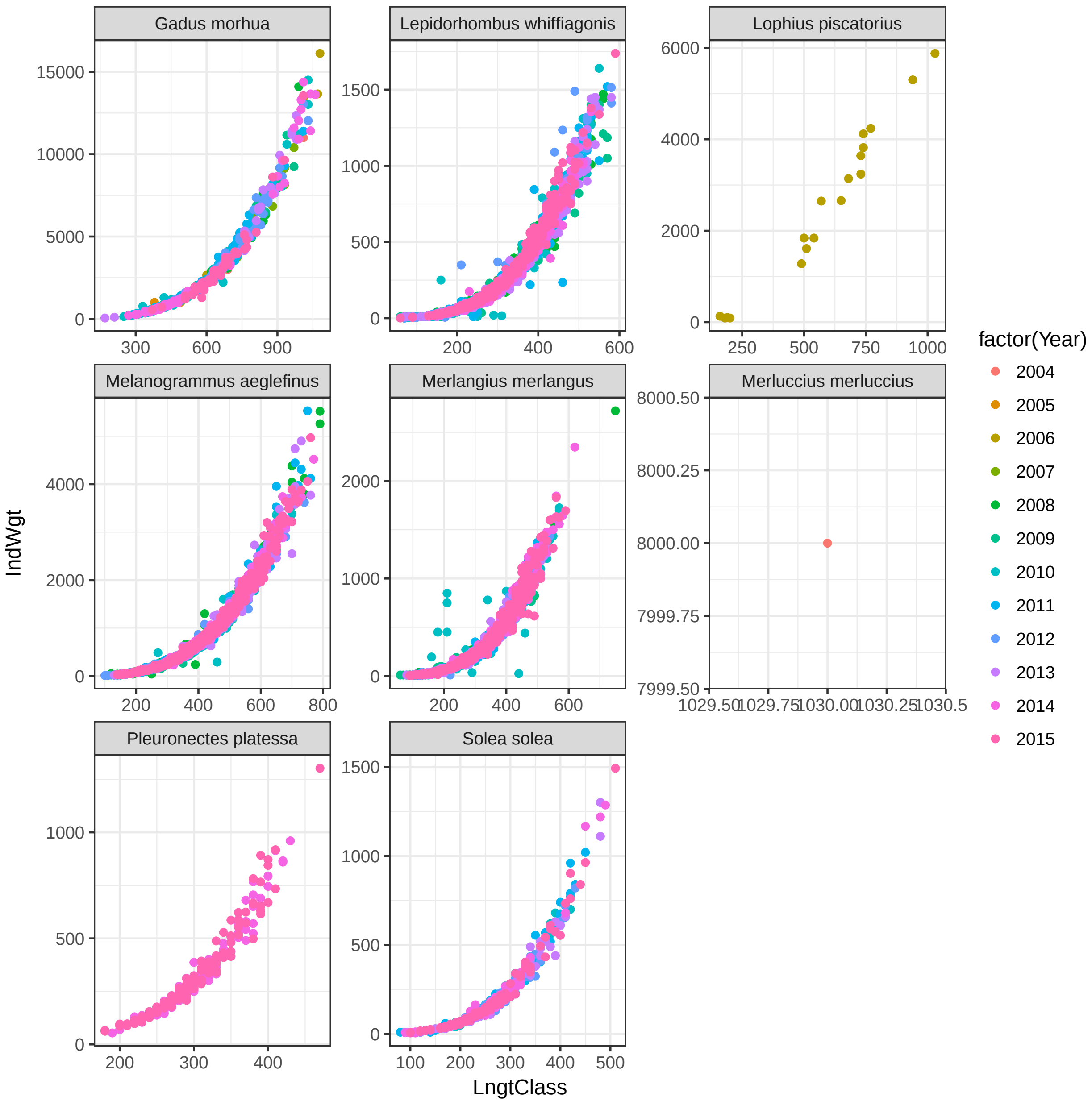

Figure 1: Estimates of individual weights at length for the gadoid species. Colours indicate individual years measurements

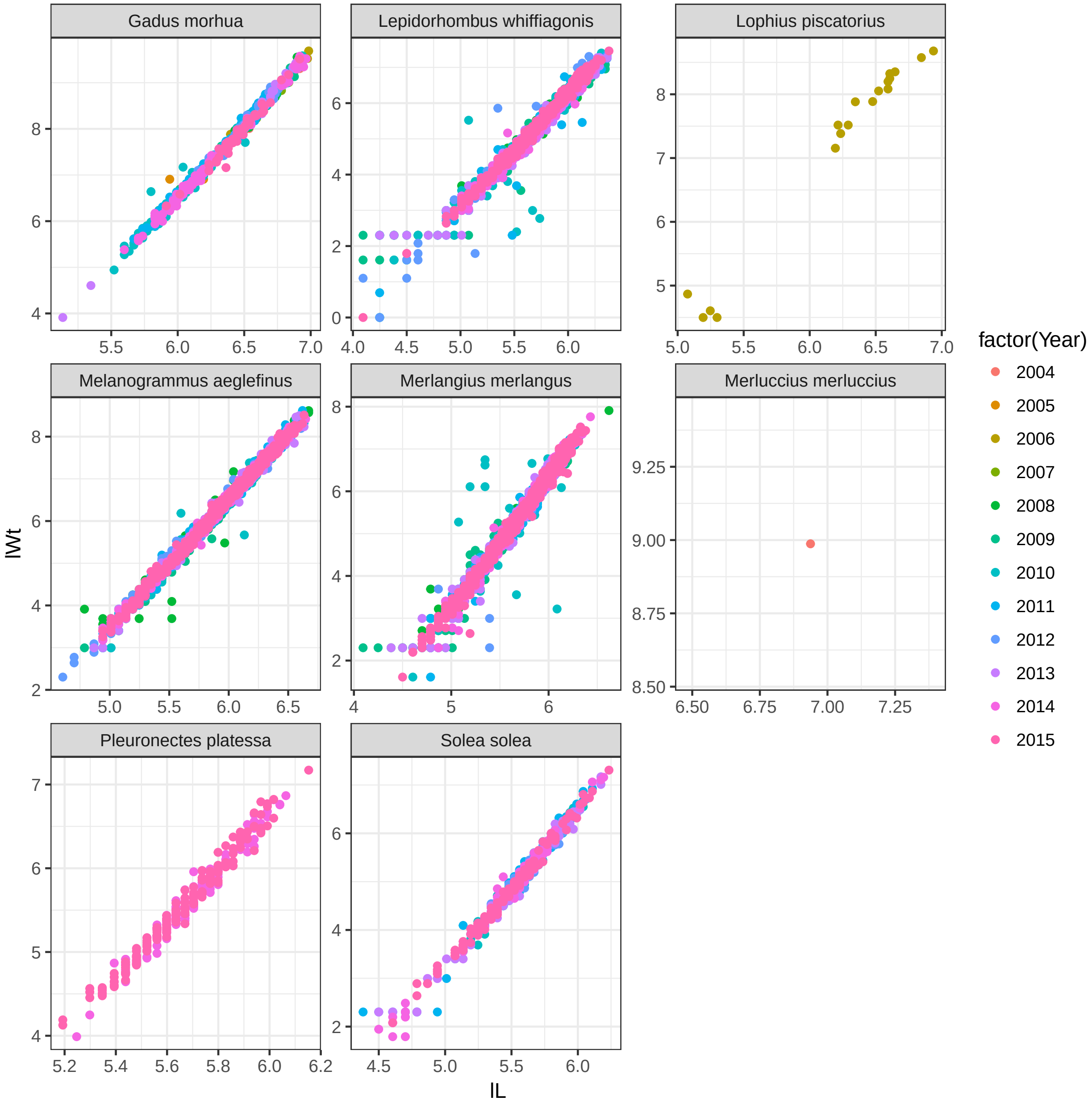

A linear model with species and year as factors was fit using the *glm* function in the base R package. We separate the roundfish and flatfish due to the different morphological forms affecting the length weight relationship:

```
gads <- c("Gadus morhua", "Melanogrammus aeglefinus", "Merluccius merlucciu
    "Merlangius merlangus")
flats <- c("Lepidorhombus whiffiagonis", "Solea solea", "Pleuronectes plate
lops <- c("Lophius piscatorius", "Lophius budegassa")

lm1.gad <- glm(lWt ~ lL + Species + Year, data = filter(DF, Species %in%
```

```
    gads))
lm2.gad <- glm(lWt ~ lL + Species, data = filter(DF, Species %in%
    gads))

stargazer(lm1.gad, lm2.gad, font.size = "small", align = T, title = "glm out
    table.placement = "H")
```

Table 1: glm output from the two model fits to gadoids

	Dependent variable:	
	lWt	
	(1)	(2)
lL	3.085***	3.085***
	(0.005)	(0.005)
SpeciesMelanogrammus aeglefinus	−0.015**	−0.015**
	(0.007)	(0.007)
SpeciesMerlangius merlangus	−0.199***	−0.199***
	(0.008)	(0.008)
SpeciesMerluccius merluccius	−0.435***	−0.436***
	(0.149)	(0.149)
Year	0.0001	
	(0.001)	
Constant	−12.263***	−11.977***
	(1.607)	(0.034)
Observations	6,624	6,624
Log Likelihood	3,219.649	3,219.633
Akaike Inf. Crit.	-6,427.297	-6,429.266
Note:	*p<0.1; **p<0.05; ***p<0.01	

```
lm1.flat <- glm(lWt ~ lL + Species + Year, data = filter(DF,
    Species %in% flats))
lm2.flat <- glm(lWt ~ lL + Species, data = filter(DF, Species %in%
    flats))

stargazer(lm1.flat, lm2.flat, font.size = "small", align = T,
```

```
    title = "glm output from the two model fits to flatfish",
    table.placement = "H")
```

Table 2: glm output from the two model fits to flatfish

	Dependent variable:	
	lWt	
	(1)	(2)
lL	3.106***	3.106***
	(0.010)	(0.010)
SpeciesPleuronectes platessa	0.361***	0.358***
	(0.013)	(0.012)
SpeciesSolea solea	0.190***	0.188***
	(0.009)	(0.009)
Year	−0.001	
	(0.002)	
Constant	−9.739***	−12.410***
	(3.147)	(0.056)
Observations	3,180	3,180
Log Likelihood	753.089	752.728
Akaike Inf. Crit.	-1,496.177	-1,497.456
Note:	*p<0.1; **p<0.05; ***p<0.01	

Year was initially also included as a factor, but found not to be significant and the second, across year, fit was chosen as the best models (Table 1, Table 2). These models were then used to predict over all lengths for each species. A bias correction was applied to adjust for the fact that the mean weights from the model fit on a log scale are geometric means on the normal scale (cf = $e^{\frac{\sigma^2}{2}}$).

For anglerfish, as there is insufficient data for a fit a model (few data points for piscatorius, no data points for budegassa), we use estimates from fishbase: $a = 0.03330$, $b = 2.766$.

```
lop <- c(a = 0.0333, b = 2.766)

predDF <- expand.grid(lL = seq(log(min(DF$LngtClass)), log(max(DF$LngtClass)),
    l = 80), Species = spp)

predDF$lWt[predDF$Species %in% gads] <- predict(lm2.gad, newdata = predDF[predDF$Species %i
```

```
    gads, ])
predDF$lWt[predDF$Species %in% flats] <- predict(lm2.flat, newdata = predDF[predDF$Species
    flats, ])
predDF$Wt[predDF$Species %in% lops] <- lop[["a"]] * (exp(predDF$lL[predDF$Species %in%
    lops])^lop[["b"]])/1000
```

```
# Exponentiate the predictions
predDF$L <- exp(predDF$lL)
predDF$Wt[predDF$Species %in% c(gads, flats)] <- exp(predDF$lWt[predDF$Species %in%
    c(gads, flats)])

## Now we need to bias correct due to the fact that the mean
## on the logscale is the geometric mean...
corr.fact.gad <- exp(sigma(lm2.gad)^2/2)
corr.fact.flat <- exp(sigma(lm2.flat)^2/2)
print(paste("Correction factor for gadoids=", round(corr.fact.gad,
    3), "and flats = ", round(corr.fact.flat, 3)))


## [1] "Correction factor for gadoids= 1.011 and flats =  1.018"


predDF$WtCorr[predDF$Species %in% gads] <- predDF$Wt[predDF$Species %in%
    gads] * corr.fact.gad
predDF$WtCorr[predDF$Species %in% flats] <- predDF$Wt[predDF$Species %in%
    flats] * corr.fact.flat
predDF$WtCorr[predDF$Species %in% lops] <- predDF$Wt[predDF$Species %in%
    lops]

# Plot the von bertalanffy fits
ggplot(DF, aes(x = LngtClass, y = IndWgt)) + geom_point(colour = "grey") +
    facet_wrap(~Species, scale = "free") + geom_line(data = predDF,
    aes(x = L, y = Wt), col = "red") + geom_line(data = predDF,
    aes(x = L, y = WtCorr), col = "blue") + theme_bw()
```

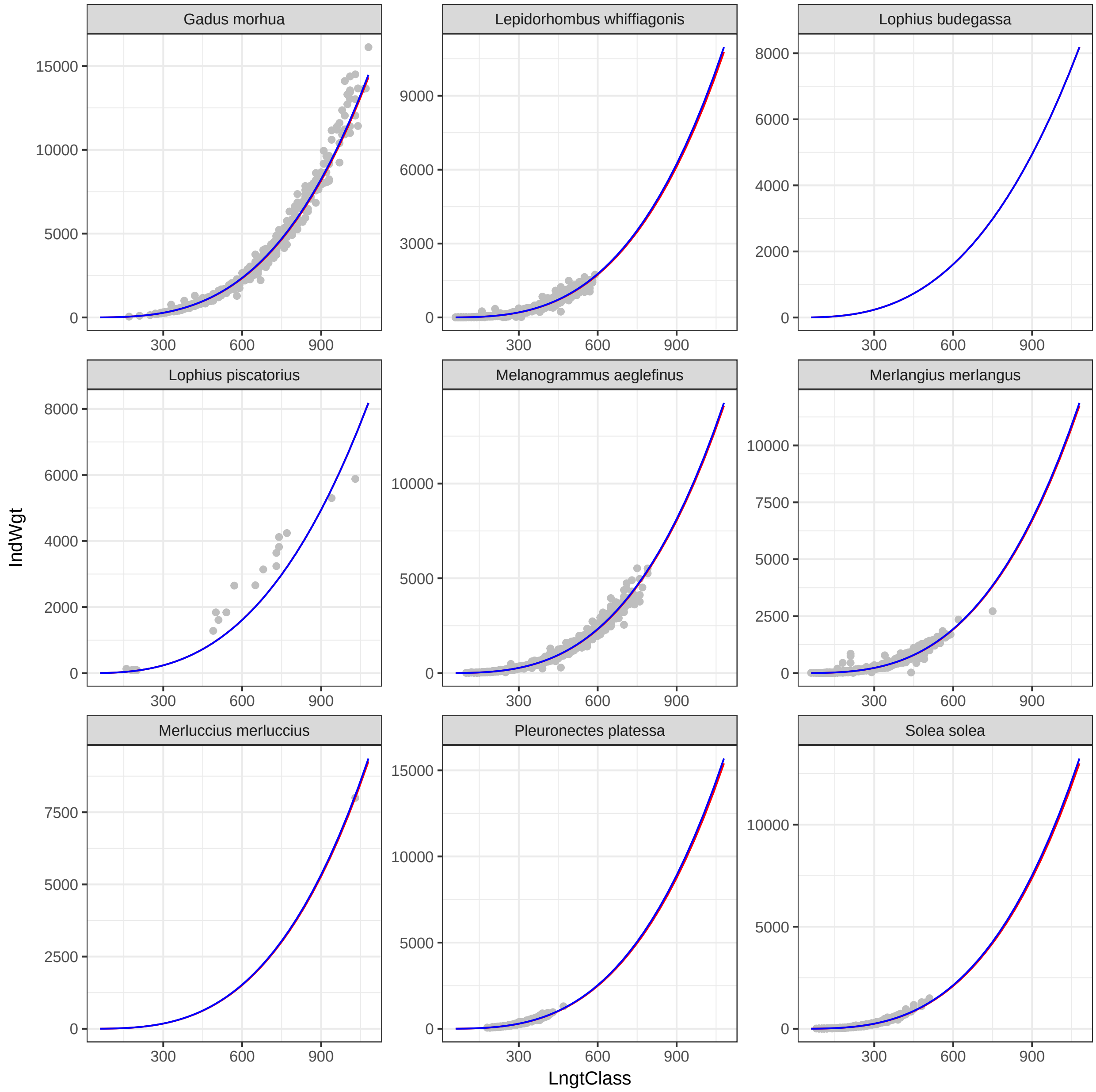


```
lm2 <- list(lm2.gad, lm2.flat)
corr.fact <- list(corr.fact.gad, corr.fact.flat)

## Save the fit and the correction factor
save(lm2, corr.fact, lop, file = file.path("DATRAS", "LengthWeightPredictCelticSea.RData"))
```

2 DATRAS data processing

Next the ICES Datras database was queried for all survey data from the Celtic Sea, extracting the haul data with the function *getHHdata* and the catch data using the function *getHLdata* from the package *icesDatras*.

The objective was to check, clean and format the data into suitable input data for the VAST model. For the Datras data this involved:

- Only retain valid hauls (excluding those at night, where there were problems with the gear etc..)
- As we want point data, calculate the midpoint of each tow based on the geodesic distance (also estimating any missing data on distance towed).
- In order to calculate swept area, obtain model estimates for any missing data points on door spread through modelling the relationship between depth and door spread.
- Calculate swept area for each tow in the surveys.
- Estimate weight at length using the length weight relationship predictions obtained from equation 1 above.
- Raise the data to weight, partioned between adult and juvenile fish.
- Merge station and catch data ensuring there is one record per species for each of the stations fished (including where there were zero catches).

2.1 Midpoint of tows

To calculate the tow midpoints, we assume tows are in a straight line and use the haversine formula (based on location in radians) to calculate the total distance.

$$Loc(R) = Loc(D) \cdot \frac{\pi}{180} \tag{3}$$

Where R and D are radians and decimal degrees respectively.

To calculate the distance:

$$fD(km) =$$
$$R \cdot \left[2 \cdot \arcsin \left(\min \left(1, \sqrt{\sin(\frac{Lat_{y1} - Lat_{y2}}{2})^2 + \cos(Lat_{x1}) \cdot \cos(Lat_{x2}) \cdot \sin(\frac{Lon_{x1} - Lon_{x2}}{2})^2} \right) \right) \right] \quad (4)$$

Where R is the mean Radius of the Earth, 6 341 km.

Total records were:

```
load(file.path("DATRAS", "CelticSurveyData.RData"))  # pre-downloaded data, HH is station,

kable(group_by(HH, Survey, HaulVal) %>% summarise(n = n()))
```

Survey	HaulVal	n
EVHOE	I	2
EVHOE	V	2643
IE-IGFS	V	2118

```
## Some initial cleaning
HH <- filter(HH, HaulVal == "V")  # only valid hauls

# Convert degrees to radians
deg2rad <- function(deg) return(deg * pi/180)

# Calculates the geodesic distance between two points
# specified by radian latitude/longitude using the Haversine
# formula (hf)
gcd.hf <- function(long1, lat1, long2, lat2) {
    R <- 6371  # Earth mean radius [km]
    delta.long <- (long2 - long1)
    delta.lat <- (lat2 - lat1)
    a <- sin(delta.lat/2)^2 + cos(lat1) * cos(lat2) * sin(delta.long/2)^2
    c <- 2 * asin(min(1, sqrt(a)))
    d = R * c
    return(d)  # Distance in km
}
############################
an <- as.numeric
```

```
HH$Dist <- mapply(gcd.hf, long1 = deg2rad(an(HH$ShootLong)),
    lat1 = deg2rad(an(HH$ShootLat)), long2 = deg2rad(an(HH$HaulLong)),
    lat2 = deg2rad(an(HH$HaulLat)))
```

```r
plot(an(HH$Distance[(HH$Distance != -9)])/1000 ~ HH$Dist[(HH$Distance !=
    -9)], main = "Recorded vs calculated distance", ylab = "Recorded distance",
    xlab = "Calculated distance", cex = 0.7)
```

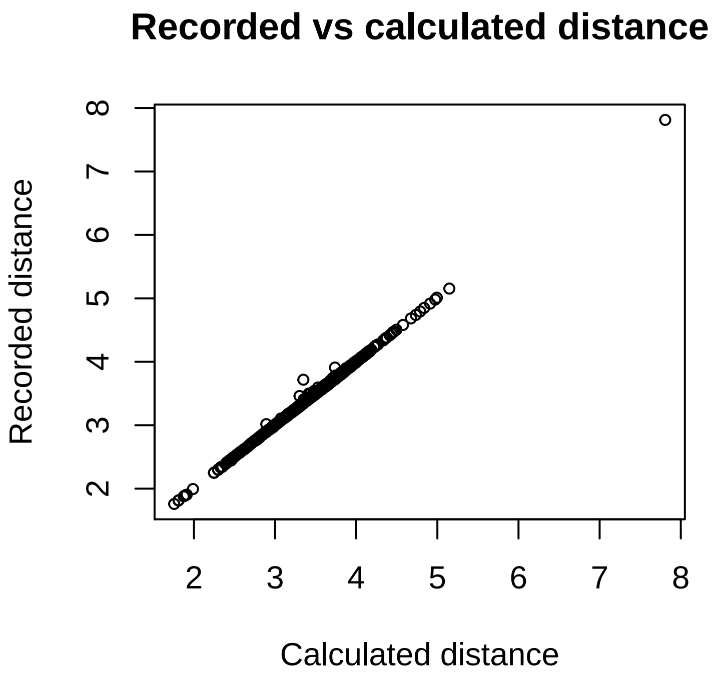


```r
## Looks good - use the calculated estimates ##
```

2.2 Swept area

To calculate the swept area, we first have to estimate the door spread for any records where it's missing. There were only 5 records with missing door spread, but use the predicted door spread for all records.

```r
# Covert numeric variables so we can explore the covariates
HH$SweepLngt <- as.numeric(HH$SweepLngt)
HH$HaulDur <- as.numeric(HH$HaulDur)
HH$DoorSpread <- as.numeric(HH$DoorSpread)
HH$Depth <- as.numeric(HH$Depth)
HH$Netopening <- as.numeric(HH$Netopening)
HH$Warplngt <- as.numeric(HH$Warplngt)
HH$Warpdia <- as.numeric(HH$Warpdia)
```

```r
HH$DoorSurface <- as.numeric(HH$DoorSurface)
HH$DoorWgt <- as.numeric(HH$DoorWgt)
HH$WingSpread <- as.numeric(HH$WingSpread)
HH$KiteDim <- as.numeric(HH$KiteDim)
HH$TowDir <- as.numeric(HH$TowDir)
HH$GroundSpeed <- as.numeric(HH$GroundSpeed)
HH$SpeedWater <- as.numeric(HH$SpeedWater)
HH$SurCurDir <- as.numeric(HH$SurCurDir)
HH$SurCurSpeed <- as.numeric(HH$SurCurSpeed)
HH$BotCurDir <- as.numeric(HH$BotCurDir)
HH$BotCurSpeed <- as.numeric(HH$BotCurSpeed)
HH$WindDir <- as.numeric(HH$WindDir)
HH$WindSpeed <- as.numeric(HH$WindSpeed)
HH$SwellDir <- as.numeric(HH$SwellDir)
HH$SwellHeight <- as.numeric(HH$SwellHeight)
HH$SurTemp <- as.numeric(HH$SurTemp)
HH$BotTemp <- as.numeric(HH$BotTemp)
HH$SurSal <- as.numeric(HH$SurSal)
HH$BotSal <- as.numeric(HH$BotSal)

HH[HH == -9] <- NA

ggplot(HH, aes(x = Depth, y = DoorSpread)) + geom_point() + theme_bw() +
    ggtitle("Relationship between depth of gear and
     door spread")
```

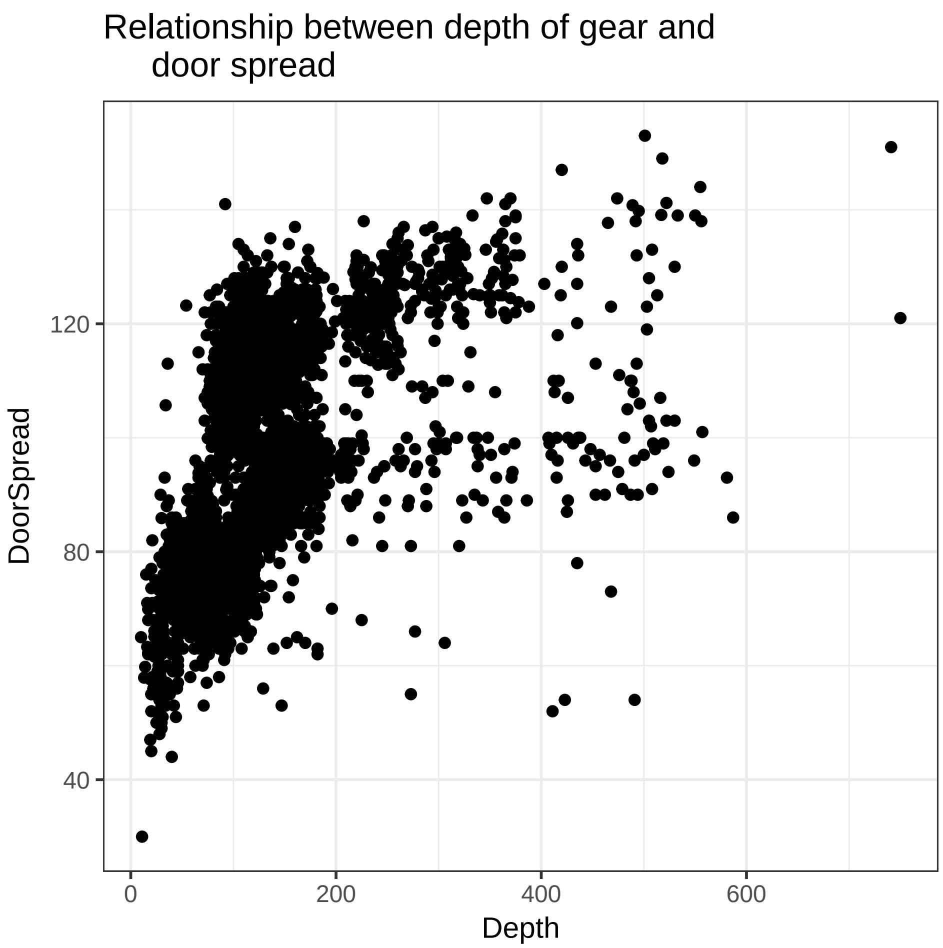

There may be another covariate affecting doorspread, indicated by the clustering of some of the data...lets look at some of them.

```
p1 <- ggplot(HH, aes(x = Depth, y = DoorSpread)) + geom_point(aes(colour = factor(DoorWgt)
    theme_bw() + ggtitle("..with door weight") + theme(legend.position = "top")

p2 <- ggplot(HH, aes(x = Depth, y = DoorSpread)) + geom_point(aes(colour = Warplngt)) +
    theme_bw() + ggtitle("..with warp length") + theme(legend.position = "top")

p3 <- ggplot(HH, aes(x = Depth, y = DoorSpread)) + geom_point(aes(colour = factor(Warpdia)
    theme_bw() + ggtitle("..with warp diameter") + theme(legend.position = "top")

p4 <- ggplot(HH, aes(x = Depth, y = DoorSpread)) + geom_point(aes(colour = factor(SweepLngt
    theme_bw() + ggtitle("..with warp sweep length") + theme(legend.position = "top")

grid.arrange(p1, p2, p3, p4, ncol = 2)
```

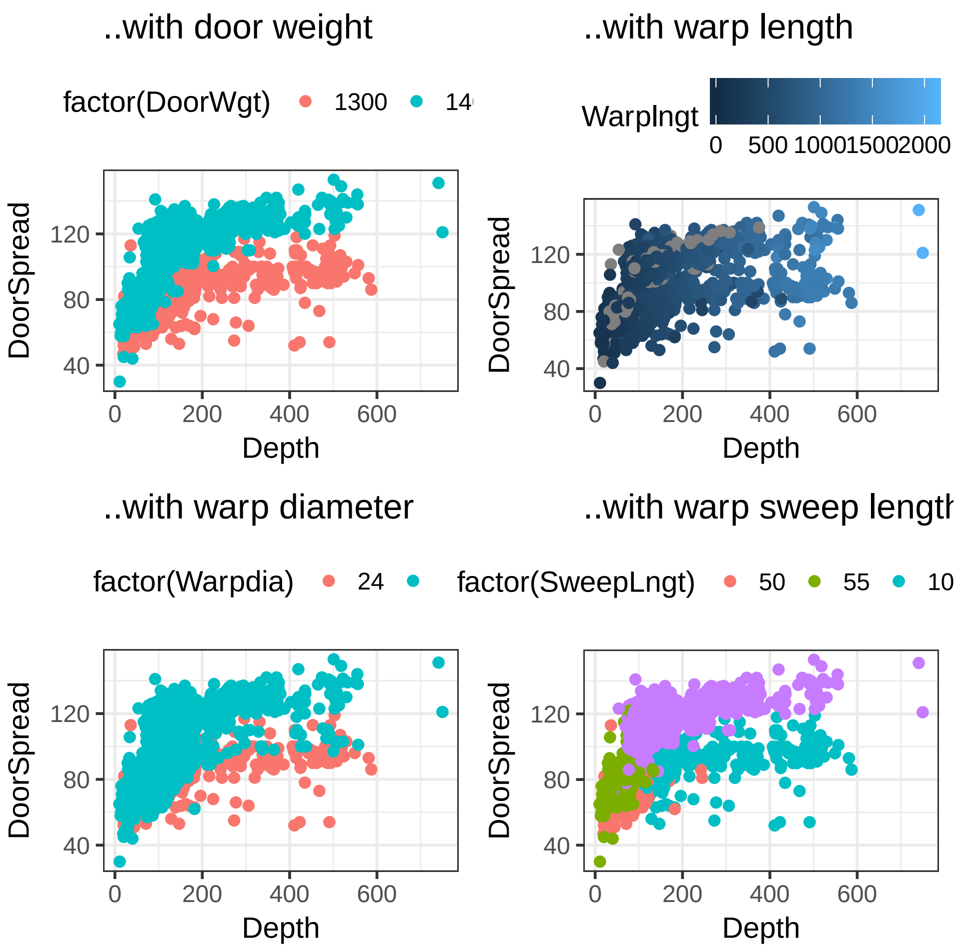

There looks to be a relationship between depth and doorspread where it increases to around 200 m and then flattens out, but with a covariate effect. We will model this relationship with a gam.

```
# Without covariate
m1 <- gam(DoorSpread ~ s(Depth), data = HH)
# summary(m1)

# With all covariate, no interactions
m2 <- gam(DoorSpread ~ s(Depth) + factor(DoorWgt) + Warplngt +
     factor(Warpdia) + factor(SweepLngt), data = HH)
# summary(m2)

### full interactions
m3 <- gam(DoorSpread ~ s(Depth) + factor(DoorWgt) * Warplngt *
     factor(Warpdia) * factor(SweepLngt), data = HH)
# summary(m3)
```

```r
kable(AIC(m1, m2, m3))
```

	df	AIC
m1	10.37315	31310.46
m2	14.90415	24524.39
m3	20.57980	24261.34

```r
# stargazer(m1,m2,m3, font.size = 'small', align = T, title =
# 'gam output from model with and without covariates',
# table.placement = 'H', single.row = T)
```

Full model looks best, but let's check the residuals against the covariates

```r
HHresid <- filter(HH, !is.na(DoorWgt), !is.na(Warplngt), !is.na(Warpdia),
    !is.na(SweepLngt), !is.na(Depth), !is.na(DoorSpread))

HHresid$residm3 <- resid(m3)
HHresid$predictm3 <- fitted(m3)

## Plot resids
ggplot(HHresid, aes(x = predictm3, y = residm3)) + geom_point() +
    geom_smooth(method = "loess", col = "red") + theme_bw() +
    ggtitle("fitted values against residuals") + geom_hline(yintercept = 0)
```

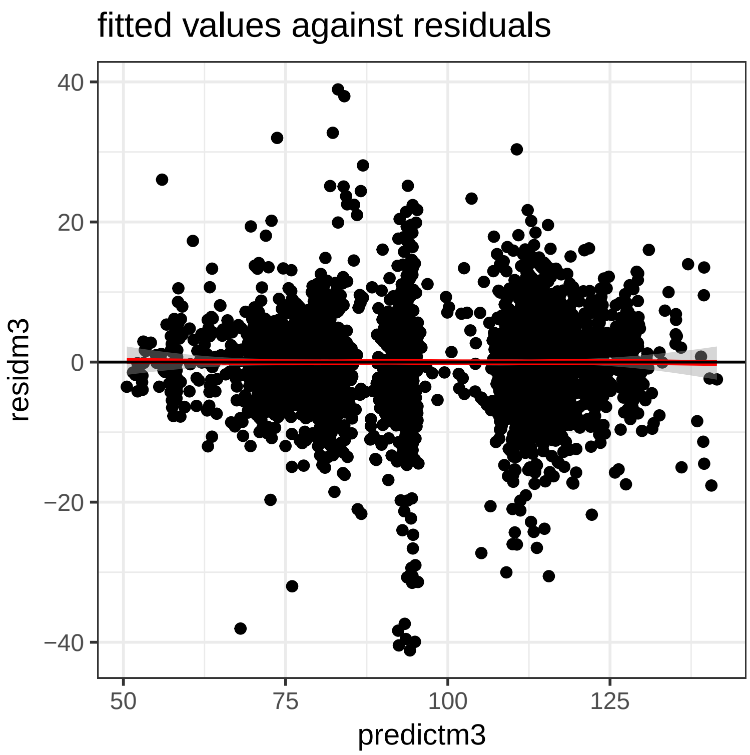

```r
p1 <- ggplot(HHresid, aes(x = factor(DoorWgt), y = residm3)) +
    geom_boxplot() + theme_bw()

p2 <- ggplot(HHresid, aes(x = Warplngt, y = residm3)) + geom_point() +
    geom_smooth(method = "loess", colour = "red") + theme_bw() +
    geom_hline(yintercept = 0)

p3 <- ggplot(HHresid, aes(x = factor(Warpdia), y = residm3)) +
    geom_boxplot() + theme_bw()

p4 <- ggplot(HHresid, aes(x = factor(SweepLngt), y = residm3)) +
    geom_boxplot() + theme_bw()

grid.arrange(p1, p2, p3, p4, ncol = 2)
```

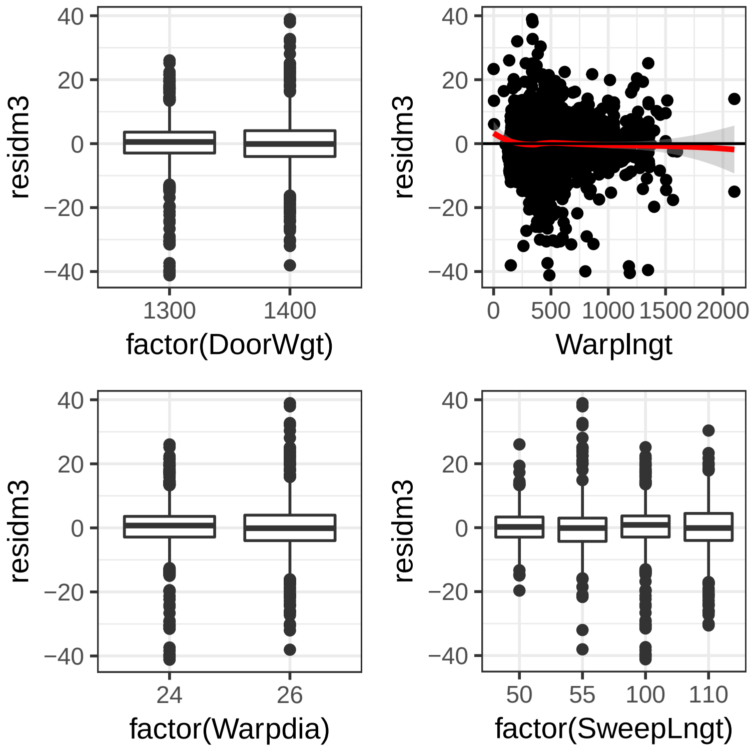

Residuals look OK, so let's look at a Q-Q plot, half-normal plot and check the predictions against the measurements...

```r
qq.gam(m3, main = "Q-Q plot")
```

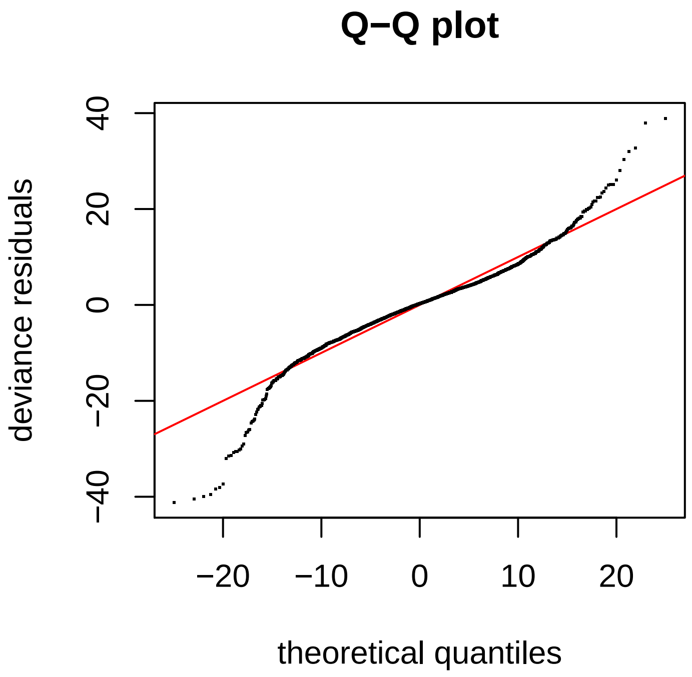


```r
faraway::halfnorm(resid(m3), main = "Half-normal plot")
```

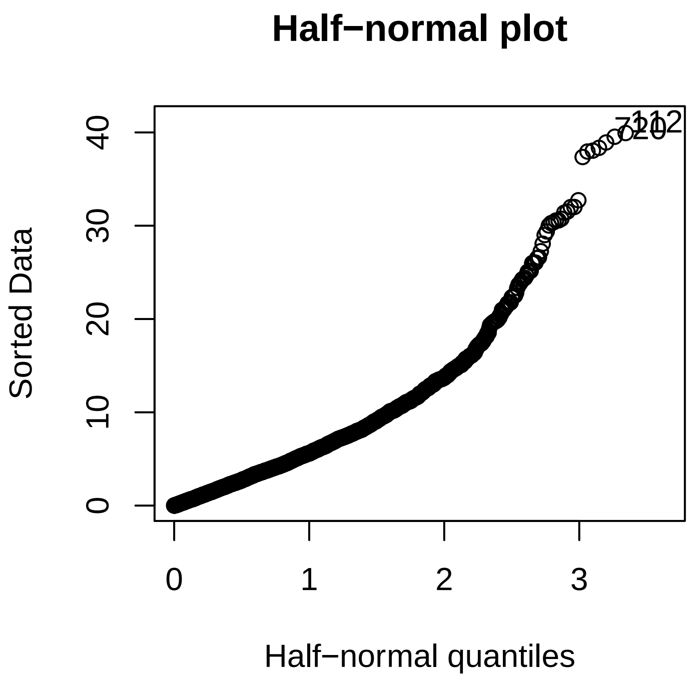

```r
HH$PredSpread <- predict(m3, newdata = HH)

ggplot(HH, aes(x = DoorSpread, y = PredSpread)) + geom_point(colour = "grey") +
    geom_abline(slope = 1, intercept = 0, col = "red") + theme_bw() +
    ylab("Predicted door spread") + xlab("Measured door spred") +
    ggtitle("Door spread predictions against measurements")
```

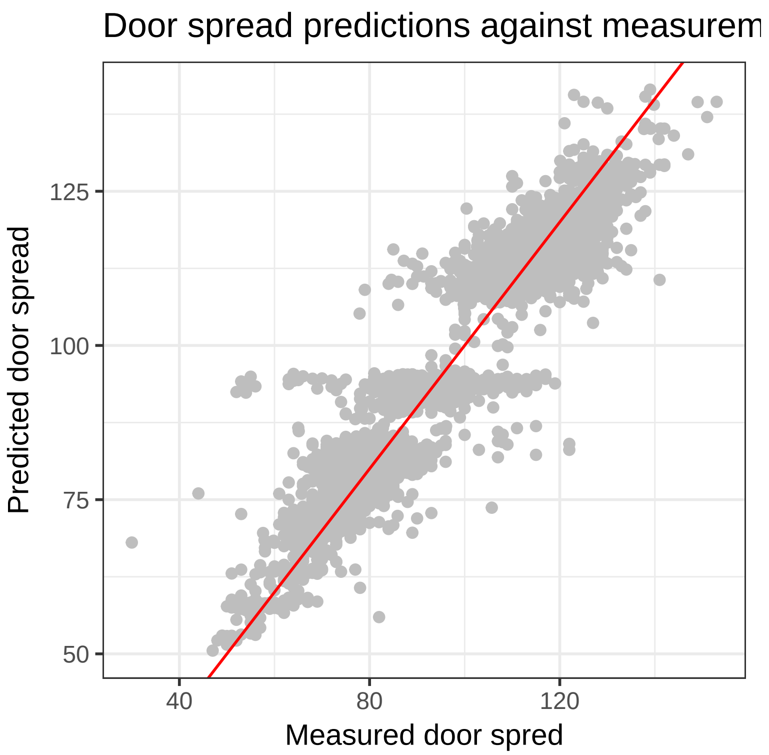


```r
nrow(HH[is.na(HH$PredSpread), ])
```

```
## [1] 209
```

```r
HH$PredSpread[is.na(HH$PredSpread) & !is.na(HH$DoorSpread)] <- HH$DoorSpread[is.na(HH$PredS
    !is.na(HH$DoorSpread)]

nrow(HH[is.na(HH$PredSpread), ])  # leaves 17 values
```

```
## [1] 17
```

```r
# Use simple depth relationship (m1) where possible
HH$PredSpread[is.na(HH$PredSpread) & !is.na(HH$Depth)] <- predict(m1,
    newdata = HH[is.na(HH$PredSpread) & !is.na(HH$Depth), ])

# For the remainder, use the standard 87 estimate
HH$PredSpread[is.na(HH$PredSpread)] <- 87
```

Looks OK. We use this to predict the door spread for the tows (filling some NAs without available covariates). Then, we calculate swept area based on:

$$SweptArea(km^2) = Distance(km) \cdot \frac{Doorspread(m)}{1000} \cdot CF \tag{5}$$

Where CF is a correction factor for the efficiency of the gear, taken from Piet et al as 0.38 for otter trawl gears. ADD REF

```
HH$SweptArea <- HH$Dist * HH$PredSpread/1000

HH$SweptAreaAdjFac <- 0.38
HH$SweptAreaAdj <- HH$SweptArea * HH$SweptAreaAdjFac

boxplot(HH$SweptAreaAdj ~ HH$Survey, ylab = "Area Swept (km2)",
    xlab = "Survey series")
```

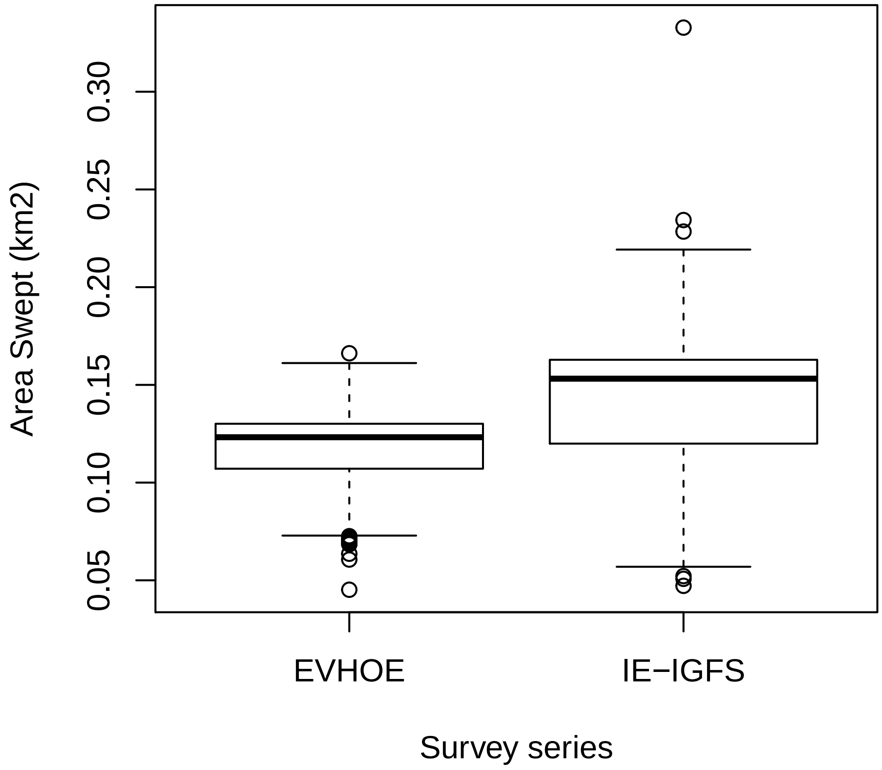

2.3 Converting to weight

The length data were converted to weight through the following process:

- Standardise unit of measurement to cm
- Add .5cm to each length group to reflect the fact that lengths are rounded down on measurement.
- Adjusting one outlier (a single whiting of 2.5 m, an order greater than actual length)
- Predict weights from the length weight relationships obtained above using equation 1.
- Multiply the number caught at length by the subfactor (fraction measured at length from the haul) and by the predicted weight at length, converting to KG.
- Relabel the species to reflect if they are juvenile or adult length. The lengths to define this split were based on the EU technical regulation defining the minimum conservation reference size (MCRS); for cod = 35 cm, haddock = 30 cm, whiting = 27 cm, hake = 27 cm, plaice = 27 cm, sole = 24 cm, megrim = 20 cm. For anglerfishes (piscatorius and budegassa) at value of 32 cm was used, equivalent to the 500 g minimum marketing weight.
- Aggregate across length classes by species.
- Merge the station information with the catch records, retaining zero entries for each species at each station, where appropriate.
- Retaining all stations within 12 W - 2 W & 48 N - 52 N (the Celtic Sea area).

The output from an estimation of the length at minimum marketing size for anglerfishes was as follows:

```
## Add species names
load(file.path("DATRAS", "DatrasSpeciesCodes.RData"))
HL$SpeciesName <- DatrasSpeciesCodes$scientific.name[match(HL$SpecCode,
    DatrasSpeciesCodes$code_number)]

# need as numeric
an <- as.numeric
HL$LngtClass <- an(HL$LngtClass)
HL$HLNoAtLngt <- an(HL$HLNoAtLngt)
HL$SubFactor <- an(HL$SubFactor)

# Deal with different length codes - standarise to cm
HL$LngtClass[(HL$LngtClass == 2460 & HL$SpeciesName == "Merlangius merlangus")] <- HL$Lngt
    2460 & HL$SpeciesName == "Merlangius merlangus")]/10  ## Dodgy datapoint!
HL$LngtClass[HL$LngtCode == ". "] <- HL$LngtClass[HL$LngtCode ==
    ". "]/10
```

```r
HL$LngtClass[HL$LngtCode == 0] <- HL$LngtClass[HL$LngtCode ==
    0]/10

# Round down length classes & add 0.5
HL$LngtClass[HL$LngtCode != "5"] <- round(HL$LngtClass[HL$LngtCode !=
    "5"])
HL$LngtClass[HL$LngtCode != "5"] <- HL$LngtClass[HL$LngtCode !=
    "5"] + 0.5

## Now raise with the Model predictions
load(file = file.path("DATRAS", "LengthWeightPredictCelticSea.RData"))

# Filter to the species of interest
HL$Species <- HL$SpeciesName
HL <- filter(HL, Species %in% spp)  ## spp from above

# Add log(length)
HL$lL <- log(HL$LngtClass * 10)

# Predict log weight gadoids
HL$LogWtLength[HL$Species %in% gads] <- predict(lm2[[1]], newdata = HL[HL$Species %in%
    gads, ])
# flats
HL$LogWtLength[HL$Species %in% flats] <- predict(lm2[[2]], newdata = HL[HL$Species %in%
    flats, ])
# anglerfishes
HL$WtLength[HL$Species %in% lops] <- (lop[["a"]] * HL$LngtClass[HL$Species %in%
    lops]^lop[["b"]])/1000

HL$WtLength[HL$Species %in% c(gads, flats)] <- exp(HL$LogWtLength[HL$Species %in%
    c(gads, flats)])  # convert back to weight in grams

HL$Wt <- HL$WtLength * HL$HLNoAtLngt * HL$SubFactor  # Total weight in g
HL$Wt <- HL$Wt/1000  # Weight in Kg
# bias correct
HL$Wt[HL$Species %in% gads] <- HL$Wt[HL$Species %in% gads] *
    corr.fact[[1]]
HL$Wt[HL$Species %in% flats] <- HL$Wt[HL$Species %in% flats] *
    corr.fact[[2]]

## And aggregate across lengths split into Ju and Ad

## For anglerfish, there is no minimum size but a minimum
## landing weight of 500g for marketing...let's find the
## equivalent length from the data

# Find the length equivalent of the 500 g
fn_opt <- function(a, b, L) {
    res <- (a * (L^b))/1000
    return(res - 0.5)
}
```

```r
# optimise
print(uniroot(f = fn_opt, a = lop[["a"]], b = lop[["b"]], interval = c(0,
    150)))
```

```
## $root
## [1] 32.35575
##
## $f.root
## [1] 1.712325e-07
##
## $iter
## [1] 10
##
## $init.it
## [1] NA
##
## $estim.prec
## [1] 6.103516e-05
```

```r
size <- uniroot(f = fn_opt, a = lop[["a"]], b = lop[["b"]], interval = c(0,
    150))$root
## Anglerfish minimum size is equivalent to 32.35 cm

lop[["a"]] * (size^lop[["b"]])/1000
```

```
## [1] 0.5000002
```

```r
lop.df <- data.frame(L = 1:150, Wt = (lop[["a"]] * (c(1:150)^lop[["b"]]))/1000)

ggplot(lop.df, aes(x = L, y = Wt)) + geom_line() + geom_segment(data = data.frame(x = size,
    x1 = size, y = 0, y1 = 0.5), aes(x = x, xend = x1, y = y,
    yend = y1), colour = "red") + geom_segment(data = data.frame(x = 0,
    x1 = size, y = 0.5, y1 = 0.5), aes(x = x, xend = x1, y = y,
    yend = y1), colour = "red") + theme_bw() + ggtitle("Lophius spp. size at minimum
\t\t\t\t\t\t  marketing weight")
```

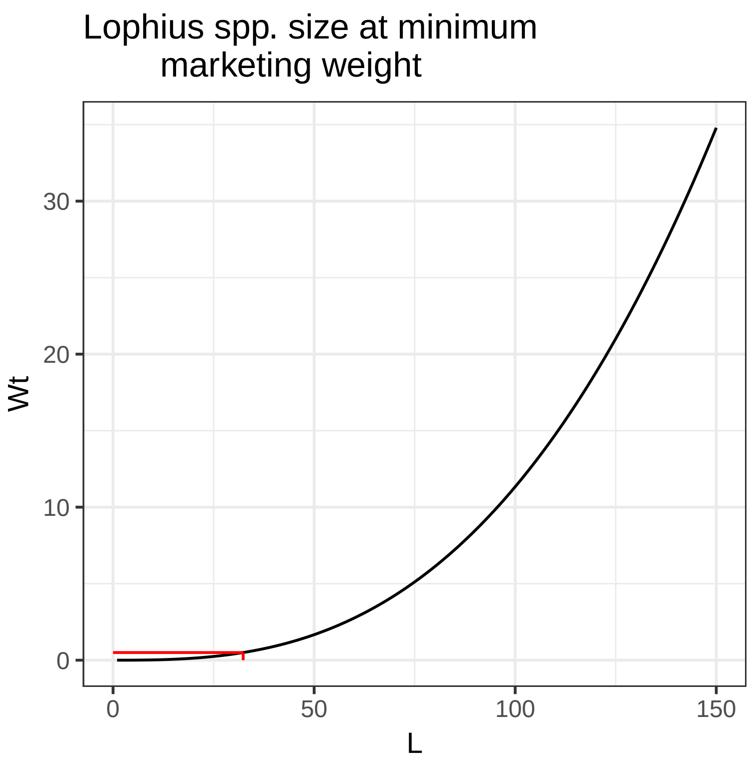


```
## Assign to length groups
HL$SpeciesName <- ifelse(HL$SpeciesName == "Gadus morhua" & HL$LngtClass <
    34.5, paste(HL$SpeciesName, "Juv", sep = "_"), ifelse(HL$SpeciesName ==
    "Gadus morhua" & HL$LngtClass >= 34.5, paste(HL$SpeciesName,
    "Adu", sep = "_"), ifelse(HL$SpeciesName == "Melanogrammus aeglefinus" &
    HL$LngtClass < 29.5, paste(HL$SpeciesName, "Juv", sep = "_"),
    ifelse(HL$SpeciesName == "Melanogrammus aeglefinus" & HL$LngtClass >=
        29.5, paste(HL$SpeciesName, "Adu", sep = "_"), ifelse(HL$SpeciesName ==
        "Merlangius merlangus" & HL$LngtClass < 26.5, paste(HL$SpeciesName,
        "Juv", sep = "_"), ifelse(HL$SpeciesName == "Merlangius merlangus" &
        HL$LngtClass >= 26.5, paste(HL$SpeciesName, "Adu", sep = "_"),
        ifelse(HL$SpeciesName == "Merluccius merluccius" & HL$LngtClass <
            26.5, paste(HL$SpeciesName, "Juv", sep = "_"), ifelse(HL$SpeciesName ==
            "Merluccius merluccius" & HL$LngtClass >= 26.5, paste(HL$SpeciesName,
            "Adu", sep = "_"), ifelse(HL$SpeciesName == "Pleuronectes platessa" &
            HL$LngtClass < 26.5, paste(HL$SpeciesName, "Juv",
            sep = "_"), ifelse(HL$SpeciesName == "Pleuronectes platessa" &
            HL$LngtClass >= 26.5, paste(HL$SpeciesName, "Adu",
            sep = "_"), ifelse(HL$SpeciesName == "Solea solea" &
            HL$LngtClass < 23.5, paste(HL$SpeciesName, "Juv",
            sep = "_"), ifelse(HL$SpeciesName == "Solea solea" &
            HL$LngtClass >= 23.5, paste(HL$SpeciesName, "Adu",
            sep = "_"), ifelse(HL$SpeciesName == "Lepidorhombus whiffiagonis" &
            HL$LngtClass >= 19.5, paste(HL$SpeciesName, "Adu",
            sep = "_"), ifelse(HL$SpeciesName == "Lepidorhombus whiffiagonis" &
            HL$LngtClass < 19.5, paste(HL$SpeciesName, "Juv",
            sep = "_"), ifelse(HL$SpeciesName == "Lophius piscatorius" &
```

```
            HL$LngtClass >= 32.5, paste(HL$SpeciesName, "Adu",
            sep = "_"), ifelse(HL$SpeciesName == "Lophius piscatorius" &
            HL$LngtClass < 32.5, paste(HL$SpeciesName, "Juv",
            sep = "_"), ifelse(HL$SpeciesName == "Lophius budegassa" &
            HL$LngtClass >= 32.5, paste(HL$SpeciesName, "Adu",
            sep = "_"), ifelse(HL$SpeciesName == "Lophius budegassa" &
            HL$LngtClass < 32.5, paste(HL$SpeciesName, "Juv",
            sep = "_"), paste(HL$SpeciesName, "All", sep = "_"))))))))))))))))))

DF <- HL[!is.na(HL$Wt), ]
DF <- DF %>% group_by(Survey, Quarter, Country, Ship, Gear, StNo,
    HaulNo, Year, SpeciesName) %>% summarise(Kg = sum(Wt)) %>%
    as.data.frame()

# Now merge in the station details: lat, lon etc..  midpoint
# of haul locations - small enough distances to not worry
# about spherical distances
HH$HaulLatMid <- (an(HH$ShootLat) + an(HH$HaulLat))/2
HH$HaulLonMid <- (an(HH$ShootLon) + an(HH$HaulLon))/2

# Fix blank spaces in variables...
DF$Survey <- gsub(" ", "", DF$Survey)
DF$Gear <- gsub(" ", "", DF$Gear)
DF$Ship <- gsub(" ", "", DF$Ship)
DF$StNo <- gsub(" ", "", DF$StNo)

## Create a haul record for each species
HH <- merge(x = HH, y = data.frame(SpeciesName = unique(DF$SpeciesName)))

# Join on the catch data
DF2 <- full_join(x = HH, y = DF)
DF2$Kg[is.na(DF2$Kg)] <- 0  #NAs are zero catches of the species

# Subset to variables of interest
DF <- DF2[c("Survey", "Ship", "StNo", "HaulNo", "Year", "Month",
    "SpeciesName", "HaulLatMid", "HaulLonMid", "HaulDur", "SweptArea",
    "SweptAreaAdj", "Kg")]

# Remove marginal areas
DF <- filter(DF, HaulLonMid < -2 & HaulLonMid > -12)
DF <- filter(DF, HaulLatMid > 48 & HaulLatMid < 52)

# Save
save(DF, file = file.path("Cleaned", "CelticSurveyFormattedSize.RData"))
```

3 Cefas survey data

The same process was undergone for the Cefas survey data. The only differences were:

- 12 040 tows were recorded as valid, with 677 either recorded invalid, abnormal or otherwise classified as irregular.
- Due to some abnormally large tow distances, a standardised tow distance (per 60 m) was calculated, and a Median Absolute Deviation (MAD) per survey series, with only standardised tow distances +- 5 times the value kept. This removed 578 outlier tows (keeping 9022).
- Swept Area sometimes reflected the use of a single or double beam trawl.
- The correction factor used was either an otter trawl value of 0.38 (as above) or a beam trawl value of 0.19, as appropriate.

```
FSS <- read.csv(file = file.path("CEFAS", "WesternSurveys_V20160905.dat"))

######################################## Process station data
Stations <- group_by(FSS, fldSeriesName, fldCruiseName, fldGearDescription,
    Year, Month, Day, Time, fldCruiseStationNumber, fldValidityCode,
    fldTowDuration) %>% summarise(ShootLat = mean(fldShotLatDecimalDegrees),
    ShootLon = mean(fldShotLonDecimalDegrees), HaulLat = mean(fldHaulLatDecimalDegrees),
    HaulLon = mean(fldHaulLonDecimalDegrees)) %>% as.data.frame()
########################################

# Keep only valid hauls
table(Stations$fldValidityCode)
Stations <- filter(Stations, fldValidityCode == "V")

###################################################################### There are some tows whic
###################################################################### distance...  so to clea
###################################################################### varies greatly over tim

############################## 3
Stations$Dist <- mapply(gcd.hf, long1 = deg2rad(Stations$ShootLon),
    lat1 = deg2rad(Stations$ShootLat), long2 = deg2rad(Stations$HaulLon),
    lat2 = deg2rad(Stations$HaulLat))

summary(Stations$Dist)

Stations$DistStand <- (Stations$Dist/Stations$fldTowDuration) *
    60

# Remove any over or under the SE of median distance for the
# survey (robust detection of outliers
# https://www.r-bloggers.com/absolute-deviation-around-the-median/)

StationsClean <- group_by(Stations, fldSeriesName) %>% summarise(median = median(DistStand)
    mean = mean(DistStand), MAD = mad(DistStand, center = median(DistStand))) %>%
    as.data.frame()

StationsClean$Up <- StationsClean$median + 5 * StationsClean$MAD
StationsClean$Lo <- StationsClean$median - 5 * StationsClean$MAD
```

```r
## Now add the upper and lower thresholds to the stations
Stations$LoThres <- StationsClean$Lo[match(Stations$fldSeriesName,
    StationsClean$fldSeriesName)]
Stations$UpThres <- StationsClean$Up[match(Stations$fldSeriesName,
    StationsClean$fldSeriesName)]

Stations$InTol <- ifelse(Stations$DistStand >= Stations$LoThres &
    Stations$DistStand <= Stations$UpThres, "KEEP", "LOSE")

table(Stations$InTol)

############################################################################ midpoint of haul [illegible]
############################################################################ worry about spher[illegible]
an <- as.numeric
Stations$HaulLatMid <- (an(Stations$ShootLat) + an(Stations$HaulLat))/2
Stations$HaulLonMid <- (an(Stations$ShootLon) + an(Stations$HaulLon))/2

## Calculate the swept area per gear for beam trawls its easy,
## for otter trawls need to include the doorspread for
## effective swept area

Surveys <- sort(unique(Stations$fldGearDescription))

Surveys <- Surveys[c(1:9, 23, 31:46, 48, 49)]
# Only keep the trawl fish surveys
print(Surveys)
Stations <- filter(Stations, fldGearDescription %in% Surveys)

## No details for otter trawl deployment, so use the standard
## 87m doorspread
Stations$GearWidth <- ifelse(Stations$fldGearDescription %in%
    Surveys[1], 2, ifelse(Stations$fldGearDescription %in% Surveys[2:5],
    3, ifelse(Stations$fldGearDescription %in% Surveys[6:9],
        4, ifelse(Stations$fldGearDescription %in% Surveys[10],
            87, ifelse(Stations$fldGearDescription %in% Surveys[11:12],
                4, ifelse(Stations$fldGearDescription %in% Surveys[13:25],
                  87, ifelse(Stations$fldGearDescription %in%
                    Surveys[26:27], 4, ifelse(Stations$fldGearDescription %in%
                    Surveys[28], 87, NA))))))))

Stations$SweptArea <- Stations$Dist * (Stations$GearWidth/1000)

## Adjust swept area for gear efficiencies, after Piet et al
## for roundfish:

# BT: 0.19 OT: 0.22 - 0.54 (Juv, ad). 0.38

Stations <- Stations[!is.na(Stations$GearWidth), ]

Stations$SweptAreaAdjFac <- sapply(Stations$GearWidth, function(x) {
    if (x %in% c(2.5, 3, 4))
```

```r
        return(0.19)
    if (x == 87)
        return(0.38) else return(NA)
})

Stations$SweptAreaAdj <- Stations$SweptArea * Stations$SweptAreaAdjFac

# Only keep stations in tolerance
Stations <- filter(Stations, InTol == "KEEP")

by(data = Stations$SweptAreaAdj, INDICES = Stations$fldSeriesName,
    FUN = mean, na.rm = T)

############################### Process the catches ####

# Convert all lengths to cm and round to 5cm size class
FSS$fldLengthGroup <- (FSS$fldLengthGroup/10) + 0.5

# load a/b parameters Add a and b parameters for
# length-weight Load the modelled length weight
# relationships....
load(file.path("DATRAS", "LengthWeightPredictCelticSea.RData"))

# Only species of interest
FSS <- filter(FSS, fldScientificName %in% toupper(spp))  # species list from above

# Add log length
FSS$lL <- log(FSS$fldLengthGroup * 10)

# Scientific names to small case except first letter
FSS$Species <- paste(toupper(substring(FSS$fldScientificName,
    1, 1)), tolower(substring(FSS$fldScientificName, 2, 1000)),
    sep = "")

# Predict log weight gads
FSS$LogWtLength[FSS$Species %in% gads] <- predict(lm2[[1]], newdata = FSS[FSS$Species %in%
    gads, ])
# flats
FSS$LogWtLength[FSS$Species %in% flats] <- predict(lm2[[2]],
    newdata = FSS[FSS$Species %in% flats, ])
# anglers
FSS$WtLength <- NA
FSS$WtLength[FSS$Species %in% lops] <- (lop[["a"]] * FSS$fldLengthGroup[FSS$Species %in%
    lops]^lop[["b"]])/1000

FSS$WtLength[FSS$Species %in% c(gads, flats)] <- exp(FSS$LogWtLength[FSS$Species %in%
    c(gads, flats)])  # convert back to weight in grams

FSS$Wt <- FSS$WtLength * FSS$Numbers  # Total weight in g
FSS$Wt <- FSS$Wt/1000  # Weight in Kg
```

```r
# bias correct
FSS$Wt[FSS$Species %in% gads] <- FSS$Wt[FSS$Species %in% gads] *
    corr.fact[[1]]
FSS$Wt[FSS$Species %in% flats] <- FSS$Wt[FSS$Species %in% flats] *
    corr.fact[[2]]

FSS <- FSS[!is.na(FSS$Wt), ]  ## Lack length measurements

# Aggregate split into Ju and Ad

FSS$Species <- ifelse(FSS$Species == "Gadus morhua" & FSS$fldLengthGroup <
    34.5, paste(FSS$Species, "Juv", sep = "_"), ifelse(FSS$Species ==
    "Gadus morhua" & FSS$fldLengthGroup >= 34.5, paste(FSS$Species,
    "Adu", sep = "_"), ifelse(FSS$Species == "Melanogrammus aeglefinus" &
    FSS$fldLengthGroup < 29.5, paste(FSS$Species, "Juv", sep = "_"),
    ifelse(FSS$Species == "Melanogrammus aeglefinus" & FSS$fldLengthGroup >=
        29.5, paste(FSS$Species, "Adu", sep = "_"), ifelse(FSS$Species ==
        "Merlangius merlangus" & FSS$fldLengthGroup < 26.5, paste(FSS$Species,
        "Juv", sep = "_"), ifelse(FSS$Species == "Merlangius merlangus" &
        FSS$fldLengthGroup >= 26.5, paste(FSS$Species, "Adu",
        sep = "_"), ifelse(FSS$Species == "Merluccius merluccius" &
        FSS$fldLengthGroup < 26.5, paste(FSS$Species, "Juv",
        sep = "_"), ifelse(FSS$Species == "Merluccius merluccius" &
        FSS$fldLengthGroup >= 26.5, paste(FSS$Species, "Adu",
        sep = "_"), ifelse(FSS$Species == "Pleuronectes platessa" &
        FSS$fldLengthGroup < 26.5, paste(FSS$Species, "Juv",
        sep = "_"), ifelse(FSS$Species == "Pleuronectes platessa" &
        FSS$fldLengthGroup >= 26.5, paste(FSS$Species, "Adu",
        sep = "_"), ifelse(FSS$Species == "Pollachius pollachius" &
        FSS$fldLengthGroup < 29.5, paste(FSS$Species, "Juv",
        sep = "_"), ifelse(FSS$Species == "Pollachius pollachius" &
        FSS$fldLengthGroup >= 29.5, paste(FSS$Species, "Adu",
        sep = "_"), ifelse(FSS$Species == "Solea solea" & FSS$fldLengthGroup <
        23.5, paste(FSS$Species, "Juv", sep = "_"), ifelse(FSS$Species ==
        "Solea solea" & FSS$fldLengthGroup >= 23.5, paste(FSS$Species,
        "Adu", sep = "_"), ifelse(FSS$Species == "Lepidorhombus whiffiagonis" &
        FSS$fldLengthGroup >= 19.5, paste(FSS$Species, "Adu",
        sep = "_"), ifelse(FSS$Species == "Lepidorhombus whiffiagonis" &
        FSS$fldLengthGroup < 19.5, paste(FSS$Species, "Juv",
        sep = "_"), ifelse(FSS$Species == "Lophius piscatorius" &
        FSS$fldLengthGroup >= 32.5, paste(FSS$Species, "Adu",
        sep = "_"), ifelse(FSS$Species == "Lophius piscatorius" &
        FSS$fldLengthGroup < 32.5, paste(FSS$Species, "Juv",
        sep = "_"), ifelse(FSS$Species == "Lophius budegassa" &
        FSS$fldLengthGroup >= 32.5, paste(FSS$Species, "Adu",
        sep = "_"), ifelse(FSS$Species == "Lophius budegassa" &
        FSS$fldLengthGroup < 32.5, paste(FSS$Species, "Juv",
        sep = "_"), paste(FSS$Species, "All", sep = "_"))))))))))))))))))))))

# Summarise as weight
FSS <- group_by(FSS, fldSeriesName, fldGearDescription, Year,
```

```
    Month, fldCruiseStationNumber, Species) %>% summarise(Kg = sum(Wt)) %>%
    as.data.frame()

## Some stations have multiple gear deployments, we want to
## have one location per station - to do so, sum the tow
## durations and swept area so we get an accurate swept area

Stations <- group_by(Stations, fldSeriesName, Year, Month, Day,
    Time, fldCruiseStationNumber, fldValidityCode, ShootLat,
    ShootLon, HaulLat, HaulLon, HaulLatMid, HaulLonMid) %>% summarise(fldTowDuration = mean
    Dist = sum(Dist), DistStand = sum(DistStand), SweptArea = sum(SweptArea),
    SweptAreaAdj = sum(SweptAreaAdj)) %>% as.data.frame()

## Also need to sum the biological data
FSS <- group_by(FSS, fldSeriesName, Year, Month, fldCruiseStationNumber,
    Species) %>% summarise(Kg = sum(Kg)) %>% as.data.frame()

## Now match the positional and catch data
Stations <- merge(x = Stations, y = data.frame(Species = unique(FSS$Species)))

FSS <- FSS[c("fldSeriesName", "Year", "Month", "fldCruiseStationNumber",
    "Species", "Kg")]

FSS <- full_join(x = Stations, y = FSS)
# Add zeros
FSS$Kg[is.na(FSS$Kg)] <- 0

by(FSS$Kg, INDICES = FSS$Species, summary)

FSS <- FSS[c("fldSeriesName", "Year", "Month", "fldCruiseStationNumber",
    "HaulLatMid", "HaulLonMid", "fldTowDuration", "SweptArea",
    "SweptAreaAdj", "Species", "Kg")]

## Trim to only keep data within core Celtic Sea
FSS <- filter(FSS, HaulLonMid > -12 & HaulLonMid < -2)  # remove extreme Lons
FSS <- filter(FSS, HaulLatMid > 48 & HaulLatMid < 52)  # remove extreme Lats

# plot(FSS$SweptAreaAdj ~ FSS$fldSeriesName)
# boxplot(FSS$SweptAreaAdj ~ FSS$Year)

table(FSS$Month, FSS$Year, FSS$fldSeriesName)

save(FSS, file = file.path(getwd(), "Cleaned", "CelticSurvey2FormattedSize.RData"))
```

4 Exploratory plots

The following section details some exploratory plots from the cleaned data. This guides the final dataset used to fit the VAST model.

```
##################################################

# Load in data
load(file.path(getwd(), "Cleaned", "CelticSurveyFormattedSize.RData"))  # Datras data by we
load(file.path(getwd(), "Cleaned", "CelticSurvey2FormattedSize.RData"))  # Cefas data by we

DWt <- DF
CWt <- FSS
rm(DF, FSS)  # Rename to avoid confusion

################################################## Combine the datasets
Wt <- data.frame(Survey = c(DWt$Survey, as.character(CWt$fldSeriesName)),
    Year = c(DWt$Year, CWt$Year), Month = c(DWt$Month, CWt$Month),
    HaulNo = c(DWt$HaulNo, CWt$fldCruiseStationNumber), Lon = c(DWt$HaulLonMid,
        CWt$HaulLonMid), Lat = c(DWt$HaulLatMid, CWt$HaulLatMid),
    HaulDur = c(DWt$HaulDur, CWt$fldTowDuration), SweptArea = c(DWt$SweptAreaAdj,
        CWt$SweptAreaAdj), Species = c(DWt$Species, CWt$Species),
    Kg = c(DWt$Kg, CWt$Kg))
rm(DWt, CWt)
```

4.1 Survey locations

The following figure shows the surveys locations each year, with each survey coloured differently.

As can be seen, initially (1982 - 1985) survey coverage was sparse and irregular, only covered by the Cefas WCGFS. From 1986 this survey becomes more regular and established, but is discontinued in 2003. The NWGFS beam trawl survey was added in 1988 and the CARLHELMAR beam trawl survey covered the western channel from 1989 until 2013.

The next significant change is the addition of the EVHOE survey in 1997, followed by the IE-IGFS survey and the Q1SWIBTS in 2003 (with the latter discontinuing in 2010). Finally, the Q1SWBEAM is added around 2006.

It is worth noting that the ICES cod and whiting assessments use a truncated survey series from the WCGFS, only using 1992 - 2004 due to changes in survey area and concerns about its impact on selectivity.

```r
Stations <- Wt[!duplicated(paste(Wt$Survey, Wt$Year, Wt$Lon,
    Wt$Lat)), ]

yrs <- sort(unique(Stations$Year))
n.yrs <- length(yrs)

map <- map_data("world", region = c("UK", "Ireland", "France"))

print(ggplot() + geom_polygon(data = map, aes(x = long, y = lat,
    group = group), colour = "black", fill = "grey") + coord_fixed(xlim = c(-12,
    2), ylim = c(48, 52), ratio = 1.3) + geom_point(data = Stations,
    aes(x = Lon, y = Lat, colour = Survey), shape = "+") + facet_wrap(~Year,
    ncol = 5) + theme_classic() + ggtitle("Survey locations by year and survey"))
```

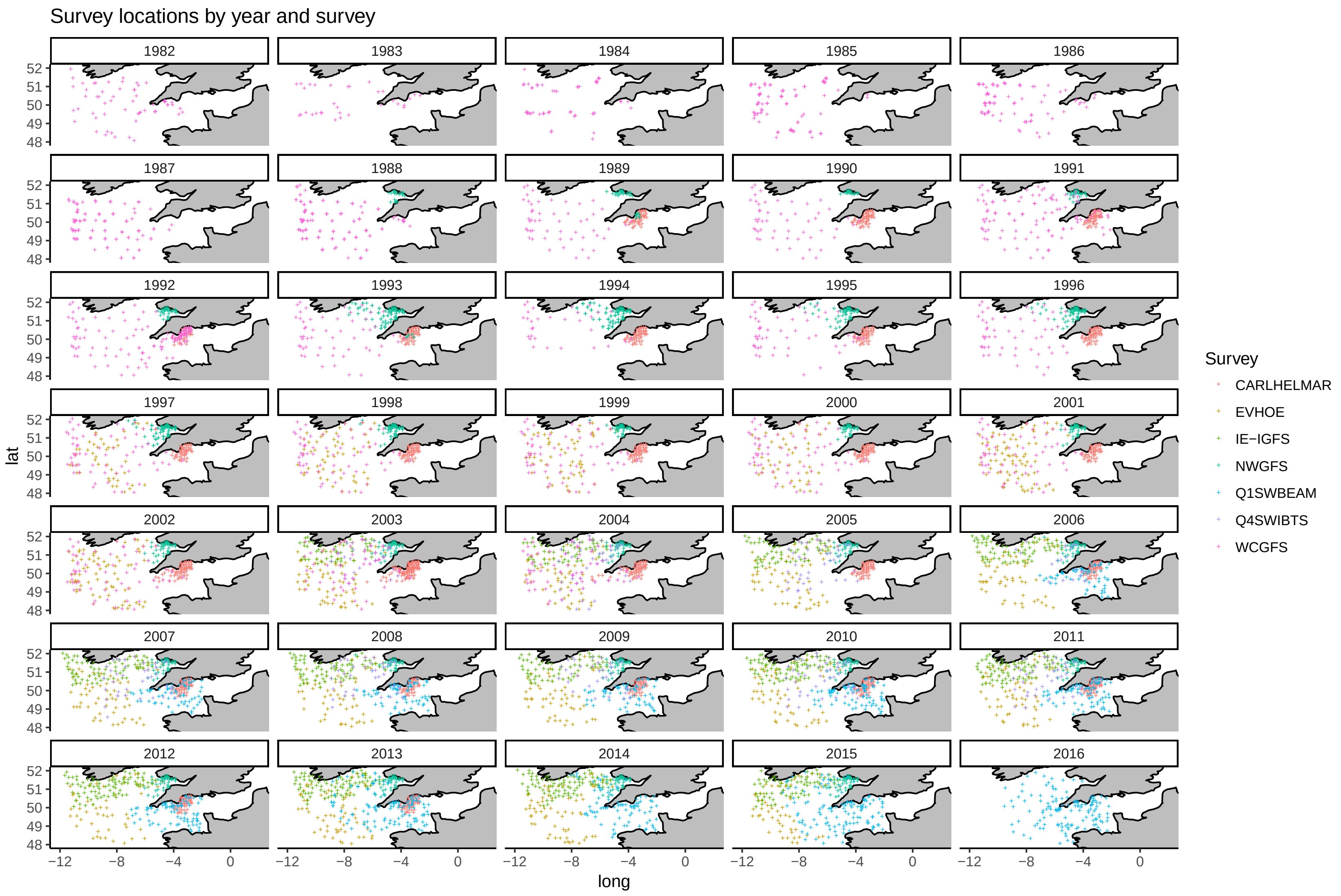
Survey locations by year and survey
1982
1983
1984
1985
1986
1987
1988
1989
1990
1991
1992
1993
1994
1995
1996
1997
1998
1999
2000
2001
2002
2003
2004
2005
2006
2007
2008
2009
2010
2011
2012
2013
2014
2015
2016
lat
long
52
51
50
49
48
−12
−8
−4
0
Survey
CARLHELMAR
EVHOE
IE−IGFS
NWGFS
Q1SWBEAM
Q4SWIBTS
WCGFS

4.2 Survey temporal coverage

The following table and plots detail the temporal coverage of the surveys. As can be seen, the number of stations was initially low (¡ 100) but increased to ¿ 200 by 1997.

The majority of survey effort is in the fourth quarter, though some survey effort is also undertaken in the first quarter

The majority of survey effort is in the fourth quarter, though some survey effort is also undertaken in the first quarter.

```
kable(table(Stations$Year, Stations$Survey))
```

	CARLHELMAR	EVHOE	IE-IGFS	NWGFS	Q1SWBEAM	Q4SWIBTS	WCGFS
1982	0	0	0	0	0	0	59
1983	0	0	0	0	0	0	32
1984	0	0	0	0	0	0	52
1985	0	0	0	0	0	0	84
1986	0	0	0	0	0	0	77
1987	0	0	0	0	0	0	88
1988	0	0	0	21	0	0	105
1989	52	0	0	51	0	0	52
1990	54	0	0	20	0	0	52
1991	50	0	0	32	0	0	100
1992	54	0	0	34	0	0	111
1993	55	0	0	105	0	0	55
1994	57	0	0	95	0	0	31
1995	53	0	0	57	0	0	54
1996	57	0	0	81	0	0	53
1997	52	46	0	86	0	0	64
1998	58	55	0	69	0	0	63
1999	56	56	0	40	0	0	64
2000	56	47	0	33	0	0	64
2001	51	76	0	37	0	0	59
2002	70	73	0	43	0	0	62
2003	128	72	49	43	0	35	48
2004	72	62	54	41	0	52	57
2005	59	67	60	41	0	40	0
2006	58	59	71	43	61	18	0
2007	58	70	77	41	64	38	0
2008	53	65	74	36	68	37	0
2009	55	59	64	43	63	32	0
2010	57	59	81	41	81	42	0
2011	57	71	86	40	80	39	0
2012	54	56	85	42	80	0	0
2013	58	63	83	42	127	0	0
2014	0	69	84	43	86	0	0
2015	0	62	68	43	126	0	0
2016	0	0	0	0	132	0	0

```r
surveyyrs <- reshape2::melt(table(Stations$Survey, Stations$Year))

print(ggplot(surveyyrs[surveyyrs$value != 0, ], aes(x = Var2,
    y = Var1)) + geom_point(aes(size = value)) + xlab("") + ylab("") +
    theme(legend.title = element_blank()) + geom_vline(xintercept = 1997) +
    ggtitle("Number of Stations Per Survey Per Year"))
```

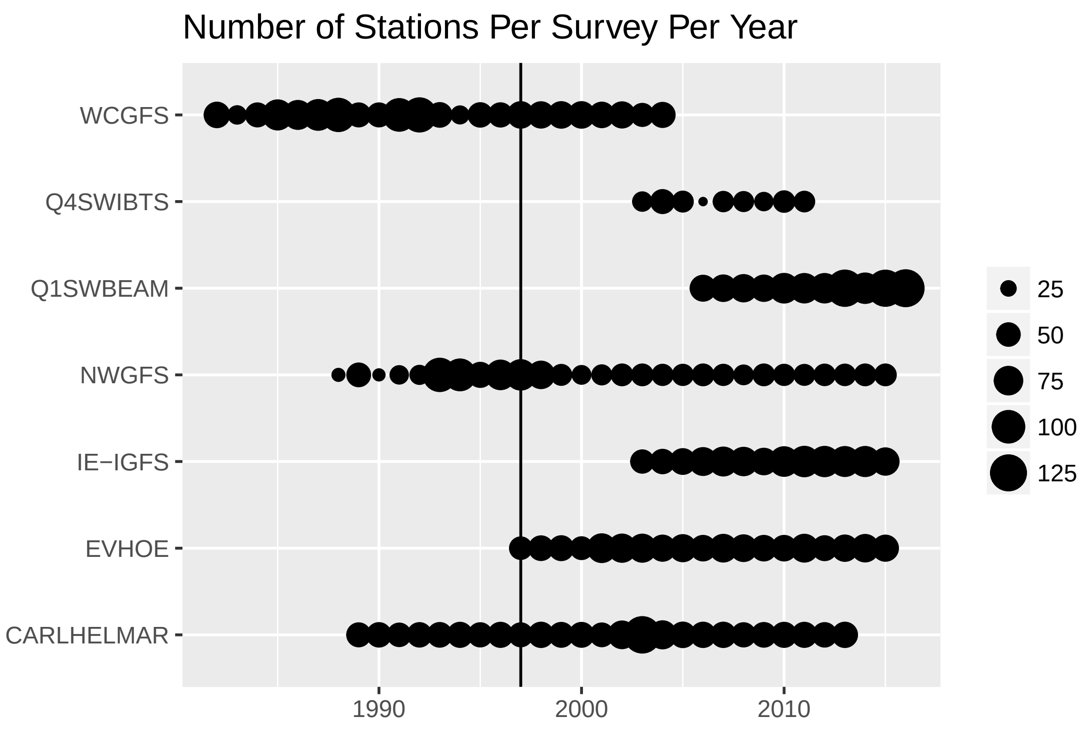


```
surveymo <- reshape2::melt(table(Stations$Month, Stations$Year))

print(ggplot(surveymo[surveymo$value != 0, ], aes(x = Var2, y = Var1)) +
    geom_point(aes(size = value)) + xlab("") + ylab("") + theme(legend.title = element_blan
    geom_vline(xintercept = 1997) + ggtitle("Number of Stations Per Survey Per Month"))
```

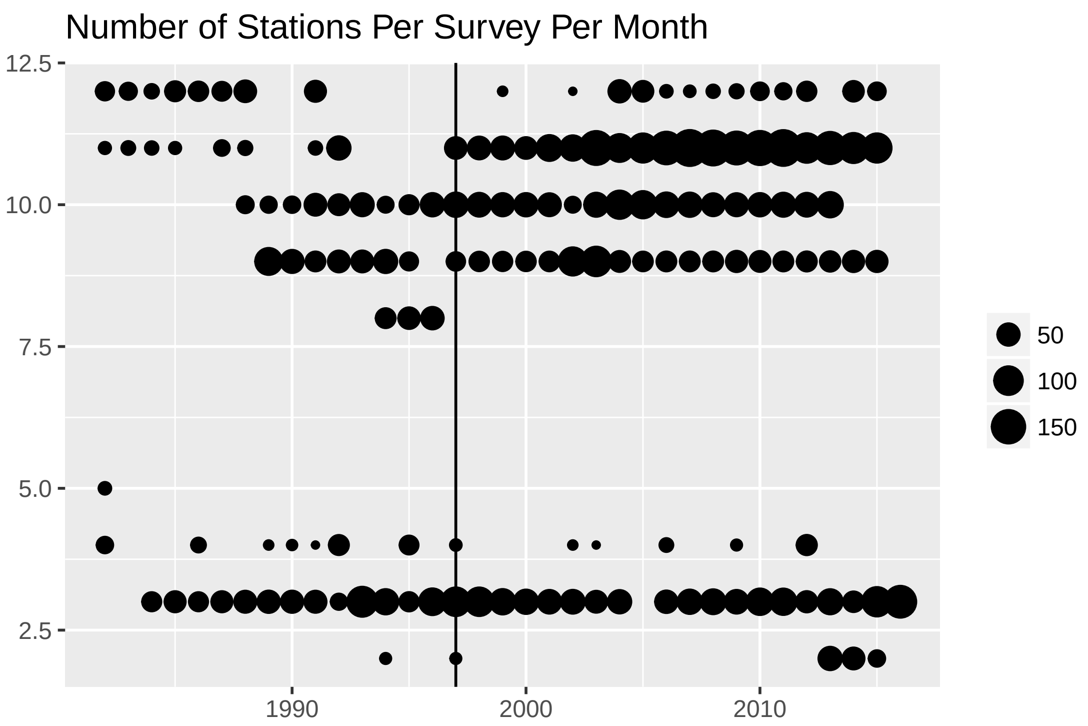


```
surveyno <- group_by(Stations, Survey, Year) %>% summarise(n = n())

print(ggplot(surveyno, aes(x = Year, y = n)) + geom_bar(stat = "identity",
    aes(fill = Survey), colour = "black") + theme_bw() + theme(axis.text.x = element_text(a
    ggtitle("No stations per year, per survey"))
```

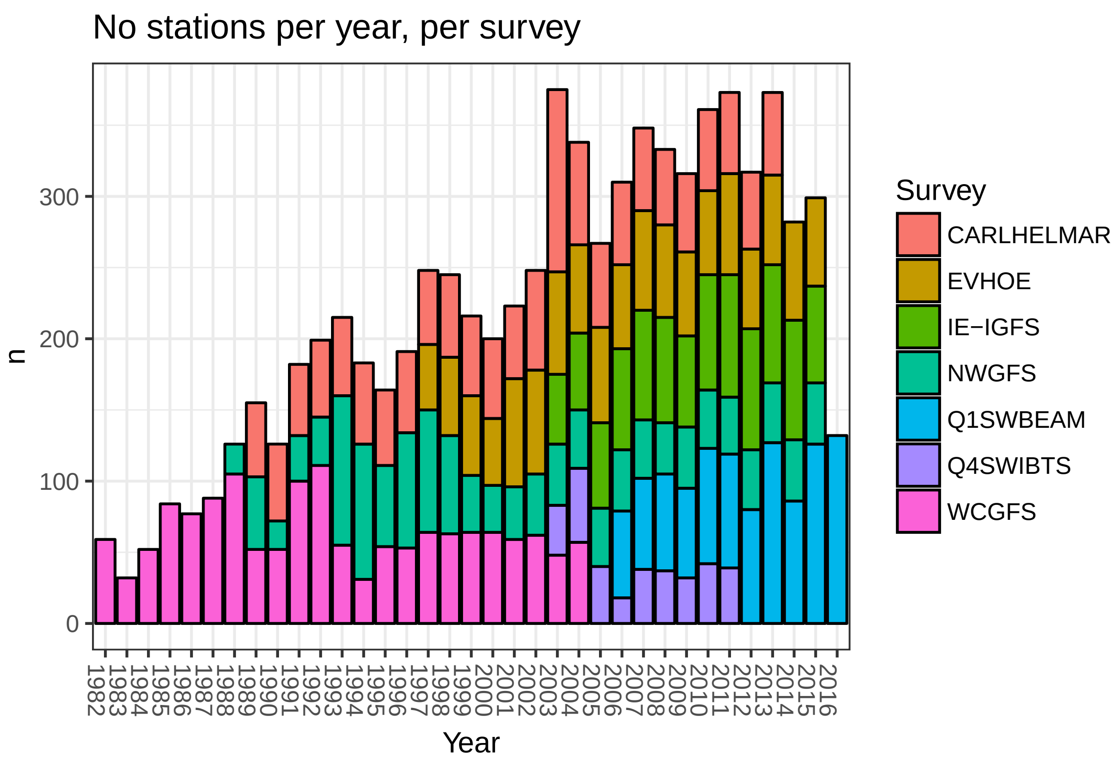

The surveys are using different gears. The main difference being that the WCGFS, IE-IGFS, EVHOE and WCGFS use otter trawl gears, while the CARLHELMAR, NWGFS, Q1SWBEAM use beam trawl gears. The WCGFS initially used hour long tows, but changes to 30 min tows consistent with other surveys later in the series.

```
boxplot(Stations$SweptArea ~ Stations$Survey, xlab = "Survey Series",
    ylab = "Swept Area (km2)", main = "Swept Area by Survey",
    cex.axis = 0.5)
axis(2, las = 1)
```

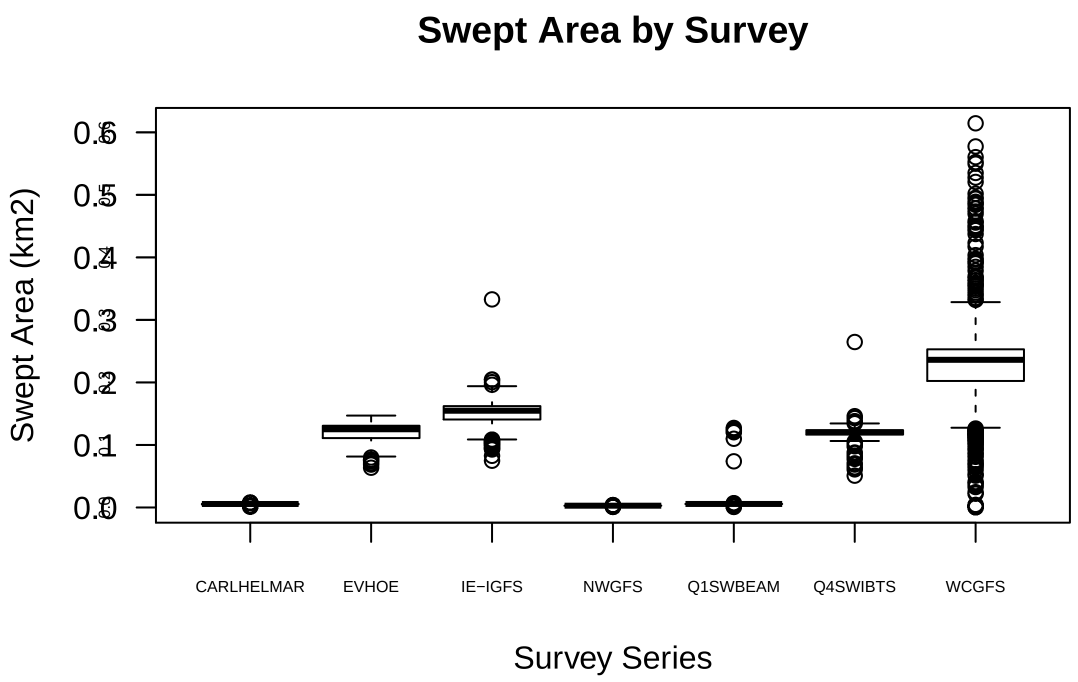


```
boxplot(as.numeric(as.character(Stations$HaulDur)) ~ Stations$Survey,
    xlab = "Survey Series", ylab = "Haul Duration (m)", main = "Haul Duration by Survey",
    cex.axis = 0.5)
```

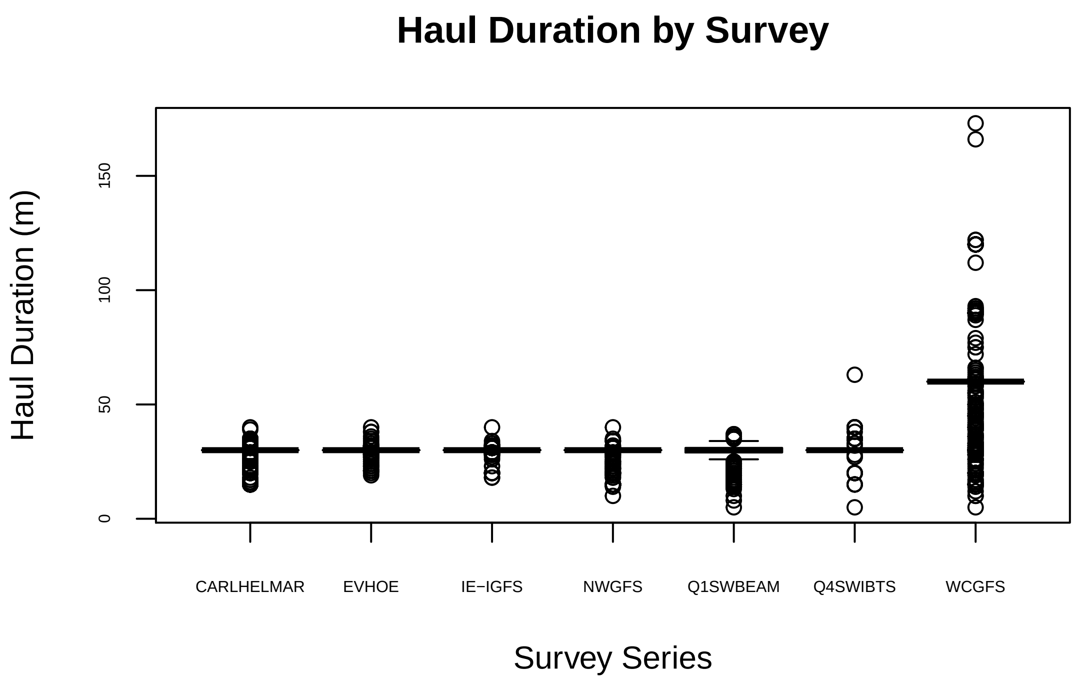

The following plots show the minimum, maximum and mean (red points) survey latitude and longitude per year, to explore changes in survey coverage.

The longitude max and min has broadly been at -2.5 to -12 for the time series, though has been more consistent since 1990. The addition of the CARLHELMAR survey in 1988 shifted the mean survey location eastwards, from around -8 to -5 degrees.

The latitudinal max and min has also generally been from 48 to 52 degrees over the time series, though this has been more consistent since 1996. The mean has generally been around 51 degrees.

```
Lats_Lons <- group_by(Stations, Year) %>% summarise(minLon = min(Lon),
    maxLon = max(Lon), meanLon = mean(Lon), minLat = min(Lat),
    maxLat = max(Lat), meanLat = mean(Lat))
print(ggplot(Lats_Lons, aes(x = Year, y = minLon)) + geom_segment(aes(xend = Year,
    yend = maxLon), lwd = 2) + geom_point(aes(y = meanLon), colour = "red") +
    theme(axis.text.x = element_text(angle = -90)) + ylim(0,
    -14) + ylab("") + xlab("") + ggtitle("Longitudinal survey coverage: min, max and mean"
```

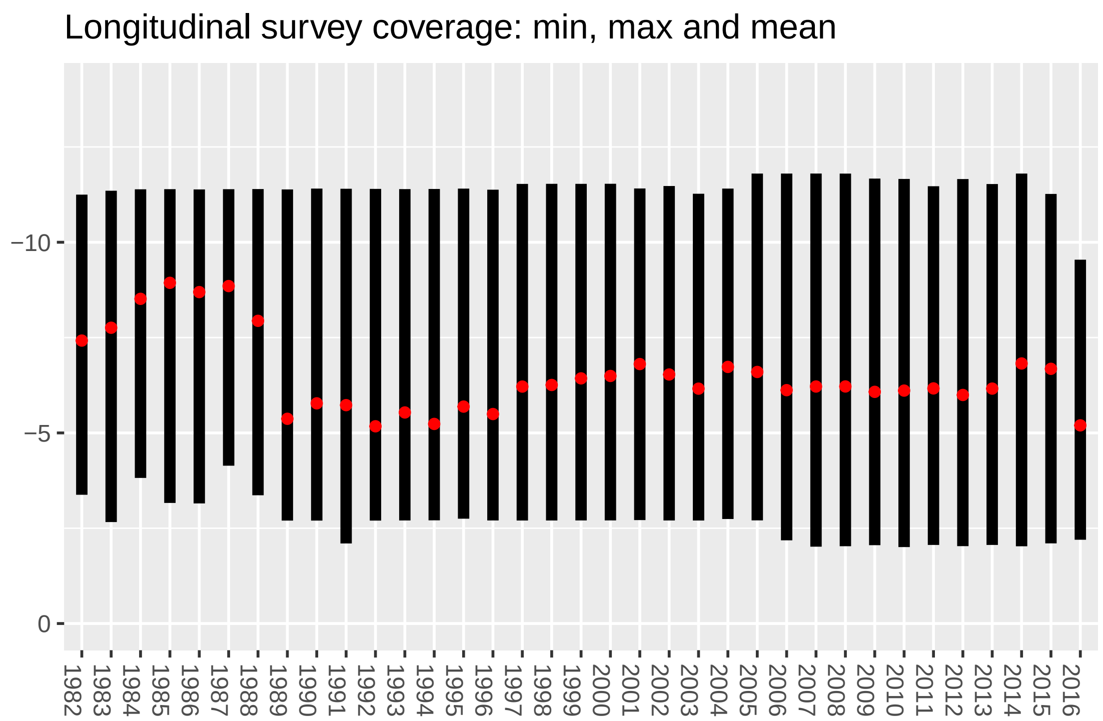


```r
print(ggplot(Lats_Lons, aes(x = Year, y = minLat)) + geom_segment(aes(xend = Year,
    yend = maxLat), lwd = 2) + geom_point(aes(y = meanLat), colour = "red") +
    theme(axis.text.x = element_text(angle = -90)) + ylim(47,
    53) + ylab("") + xlab("") + ggtitle("Latitudinal survey coverage: min, max and mean"))
```

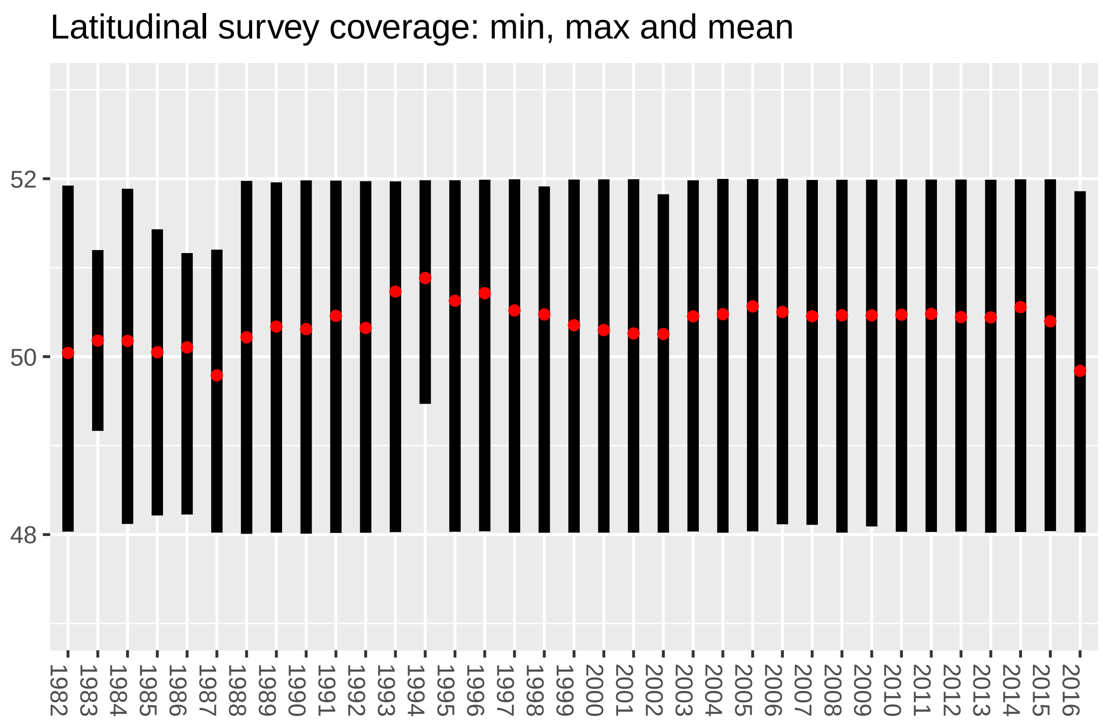

The following details the total catch by year, by survey. As can be seen, the IE-IGFS, EVHOE, WCGFS, Q4SWIBTS and Q1SWBEAM catch reasonable quantities of gadoids, while the CARLHELMAR and NWGFS catch very little.

```
tot <- group_by(Wt, Survey, Species, Year) %>% summarise(Kg = sum(Kg))

print(ggplot(tot, aes(x = Year, y = Kg)) + geom_bar(stat = "identity",
    aes(fill = Species)) + facet_wrap(~Survey, ncol = 2) + theme(legend.position = "bottom'
    axis.text.x = element_text(angle = -90)))
```

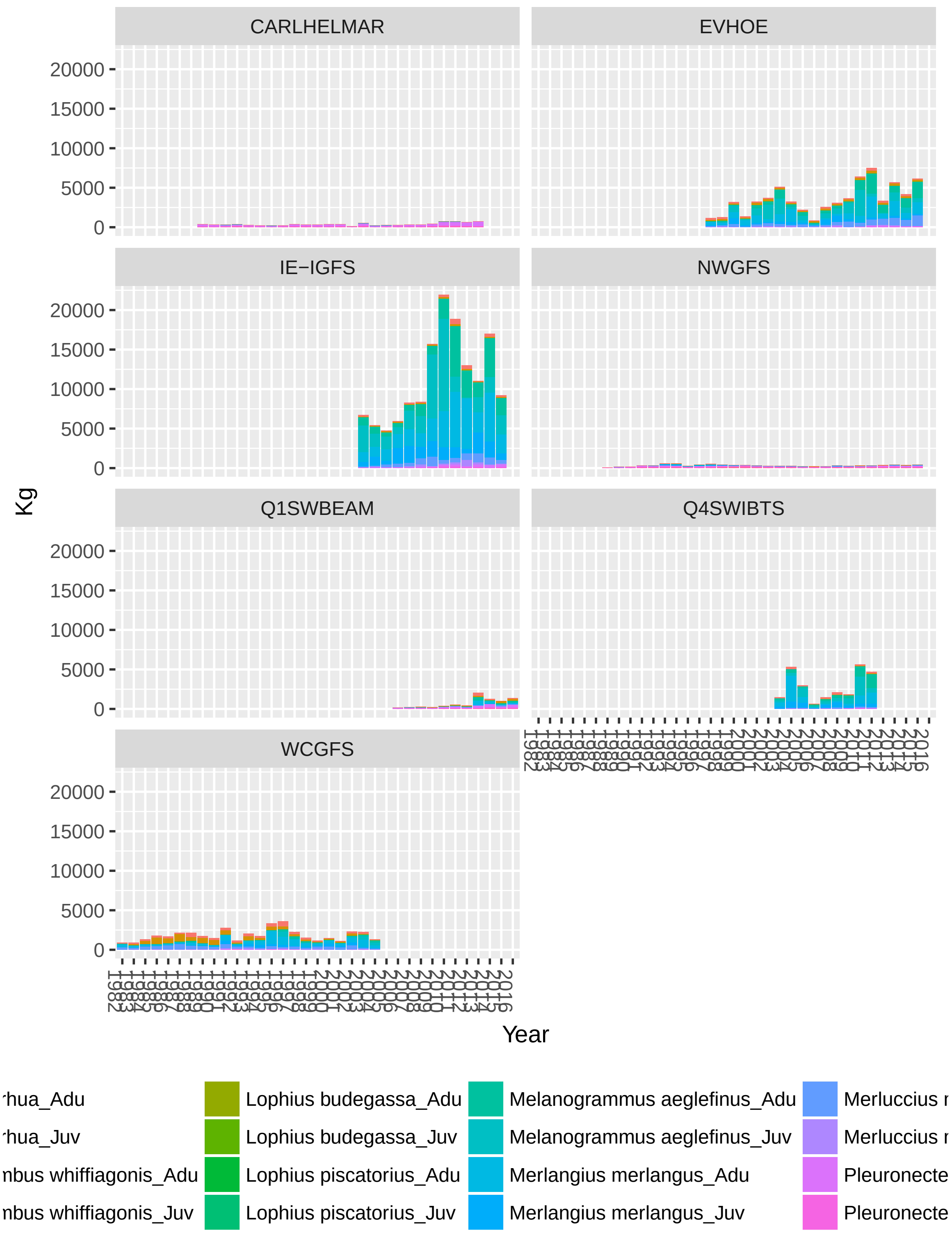

We need to check on the proportion of zeros in the data (for the delta model)...

```
## Proportion of zeros for each species/year
yrs <- sort(unique(Wt$Year))
spp <- sort(unique(Wt$Species))

PropZeros <- matrix(NA, nrow = length(yrs), ncol = length(spp))

for (y in 1:length(yrs)) {
    for (s in 1:length(spp)) {
        tmp <- filter(Wt, Year == yrs[y], Species == spp[s])
        PropZeros[y, s] <- nrow(tmp[tmp$Kg == 0, ])/length(tmp$Kg)
    }
}

PropZeros <- as.data.frame(PropZeros)
colnames(PropZeros) <- spp
PropZeros$Year <- yrs

x <- reshape2::melt(PropZeros, id = "Year")
x$col <- ifelse(x$value == 0 | x$value == 1, "all zeros or none",
    "OK")

ggplot(x, aes(x = Year, y = variable)) + geom_point(aes(size = value,
    col = factor(col))) + theme_bw() + theme(axis.text.x = element_text(angle = -90))
```

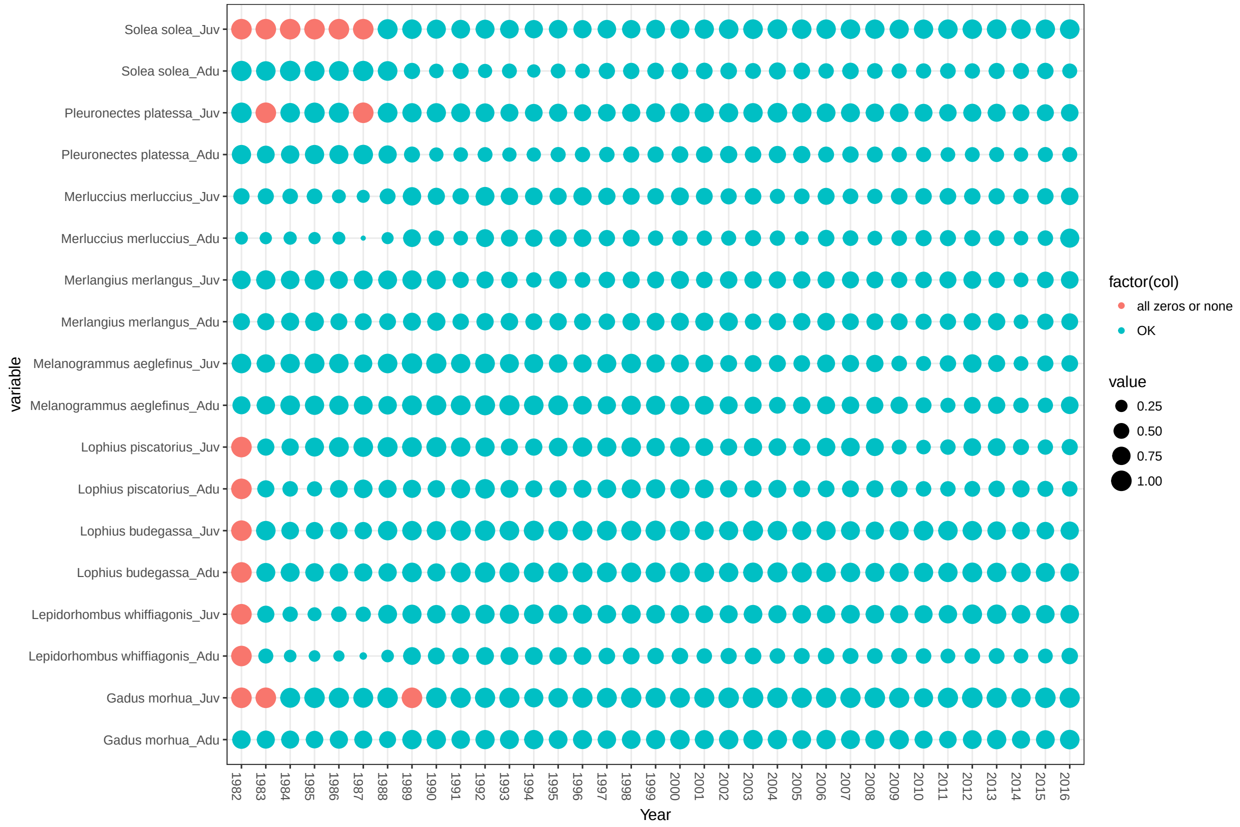

The next pages detail the spatial catch distribution of the different species, followed by the catch per unit effort for the different survey series for each species.

```r
spp <- sort(unique(Wt$Species))

for (s in 1:length(spp)) {

    plotDF <- Wt[Wt$Species == spp[s], ]

    print(ggplot() + geom_polygon(data = map, aes(x = long, y = lat,
        group = group), colour = "black", fill = "grey") + coord_fixed(xlim = c(-12,
        2), ylim = c(48, 52), ratio = 1.3) + geom_point(data = plotDF[plotDF$Kg !=
        0, ], aes(x = Lon, y = Lat, size = sqrt(Kg)), colour = "blue",
        alpha = 0.5) + scale_size_continuous(limits = range(sqrt(Wt$Kg))) +
        geom_point(data = plotDF[plotDF$Kg == 0, ], aes(x = Lon,
            y = Lat), colour = "red", shape = "+") + facet_wrap(~Year,
        ncol = 5) + theme_classic() + ggtitle(paste("Spatial catches of",
        spp[s], "in Kg", sep = " ")))
}
```

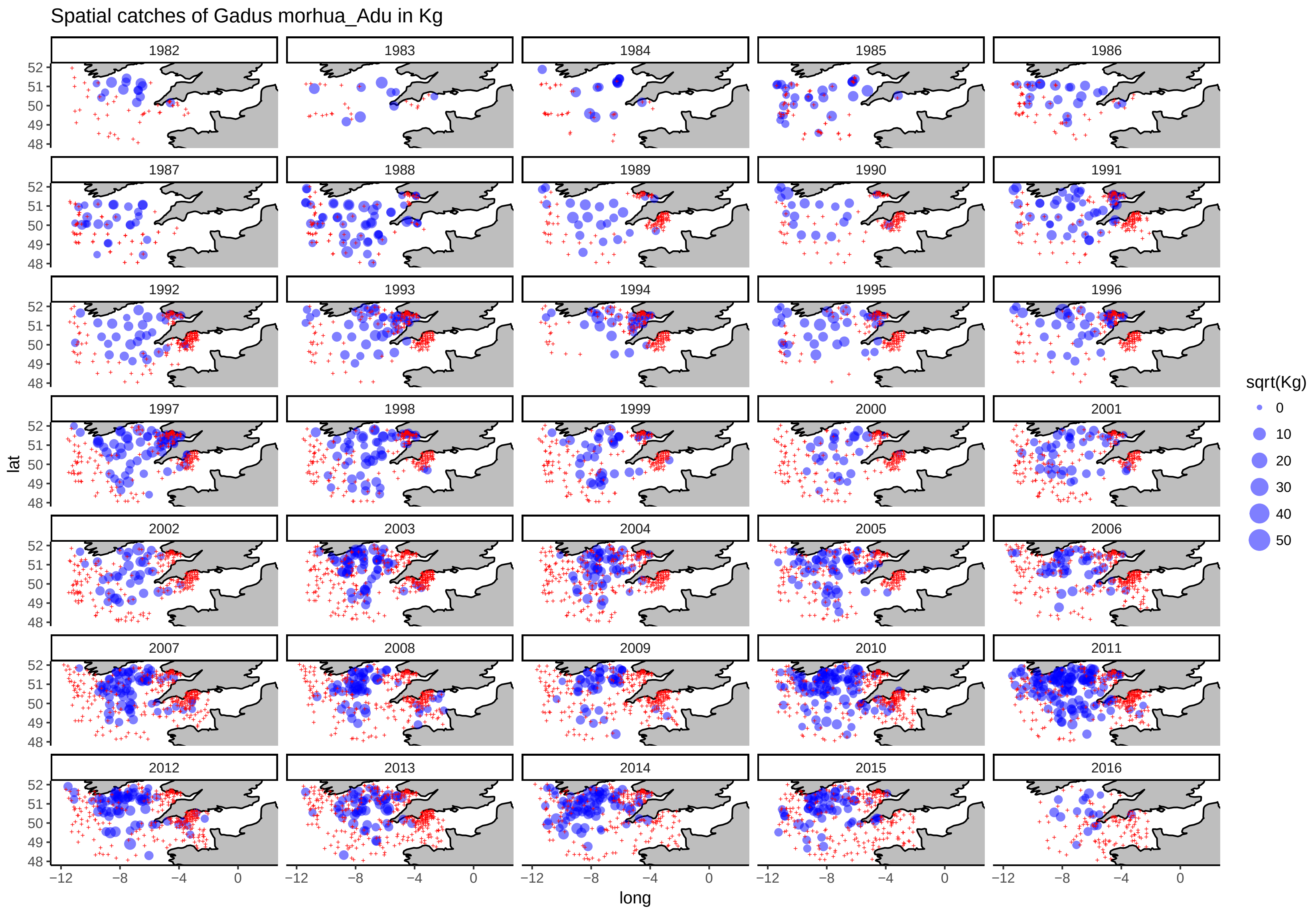
Spatial catches of Gadus morhua_Adu in Kg
1982
1983
1984
1985
1986
1987
1988
1989
1990
1991
1992
1993
1994
1995
1996
1997
1998
1999
2000
2001
2002
2003
2004
2005
2006
2007
2008
2009
2010
2011
2012
2013
2014
2015
2016
lat
52
51
50
49
48
long
−12
−8
−4
0
sqrt(Kg)
0
10
20
30
40
50

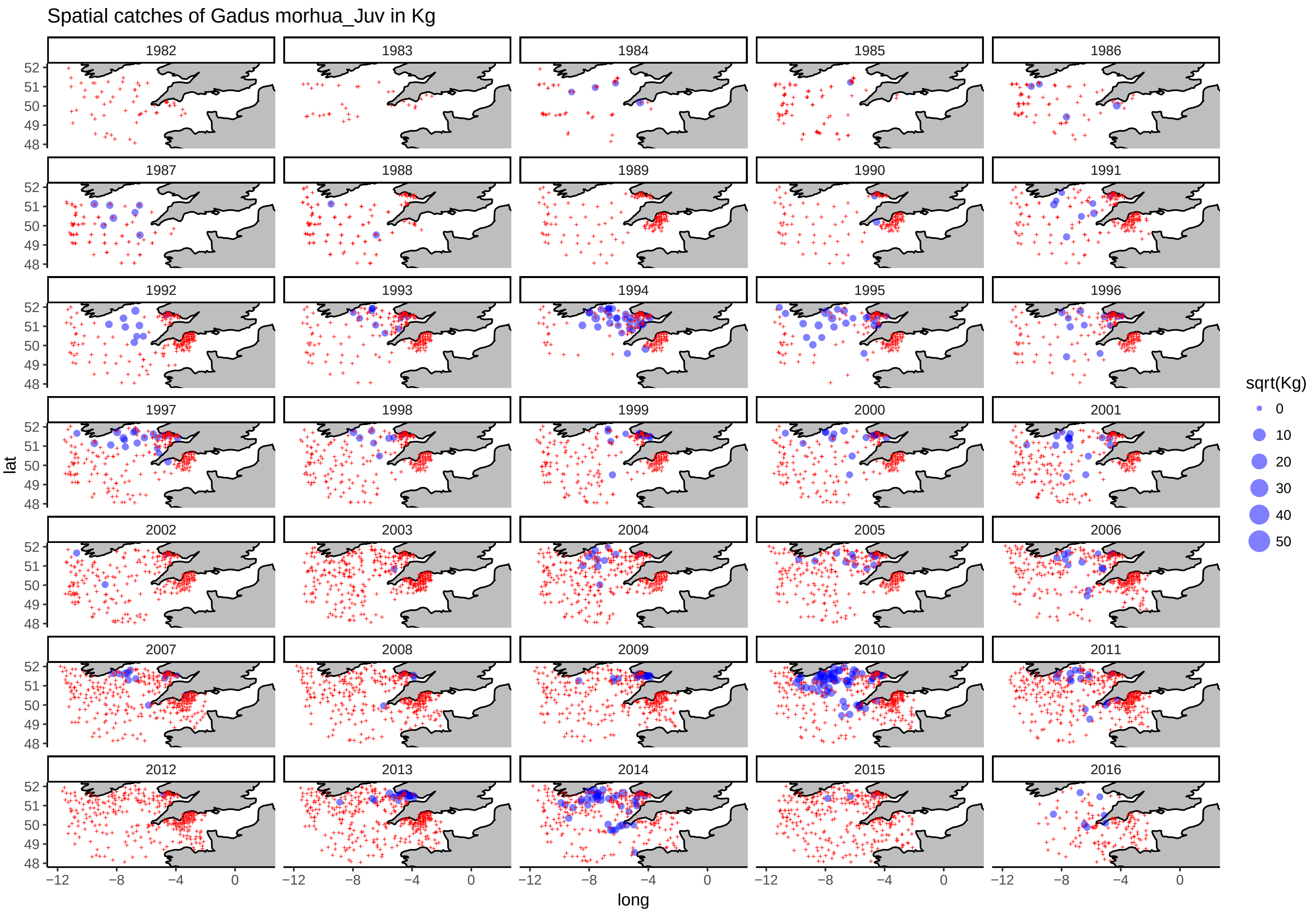
Spatial catches of Gadus morhua_Juv in Kg
1982
1983
1984
1985
1986
1987
1988
1989
1990
1991
1992
1993
1994
1995
1996
1997
1998
1999
2000
2001
2002
2003
2004
2005
2006
2007
2008
2009
2010
2011
2012
2013
2014
2015
2016
lat
52
51
50
49
48
−12
−8
−4
0
long
sqrt(Kg)
0
10
20
30
40
50

Spatial catches of Lepidorhombus whiffiagonis_Adu in Kg

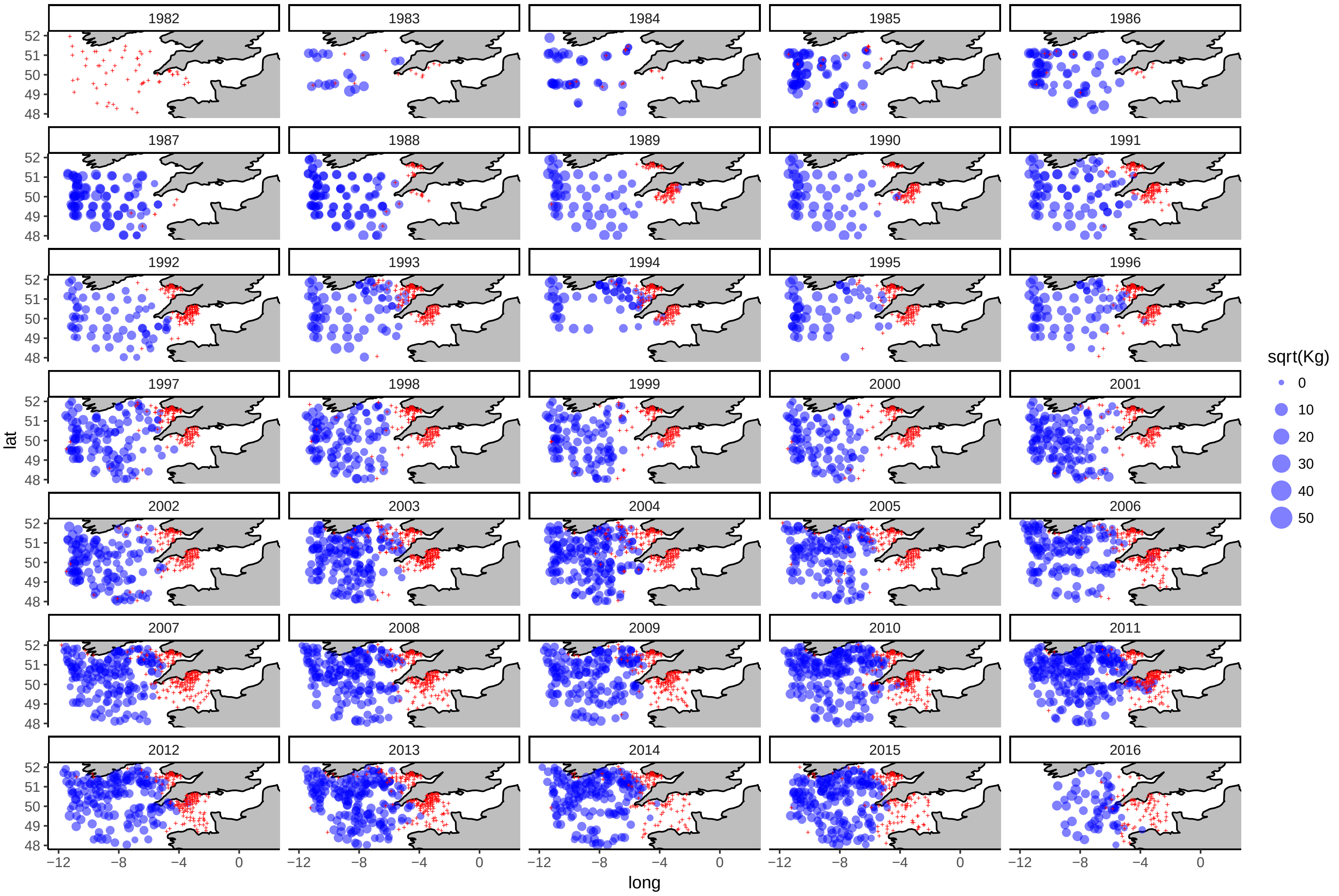

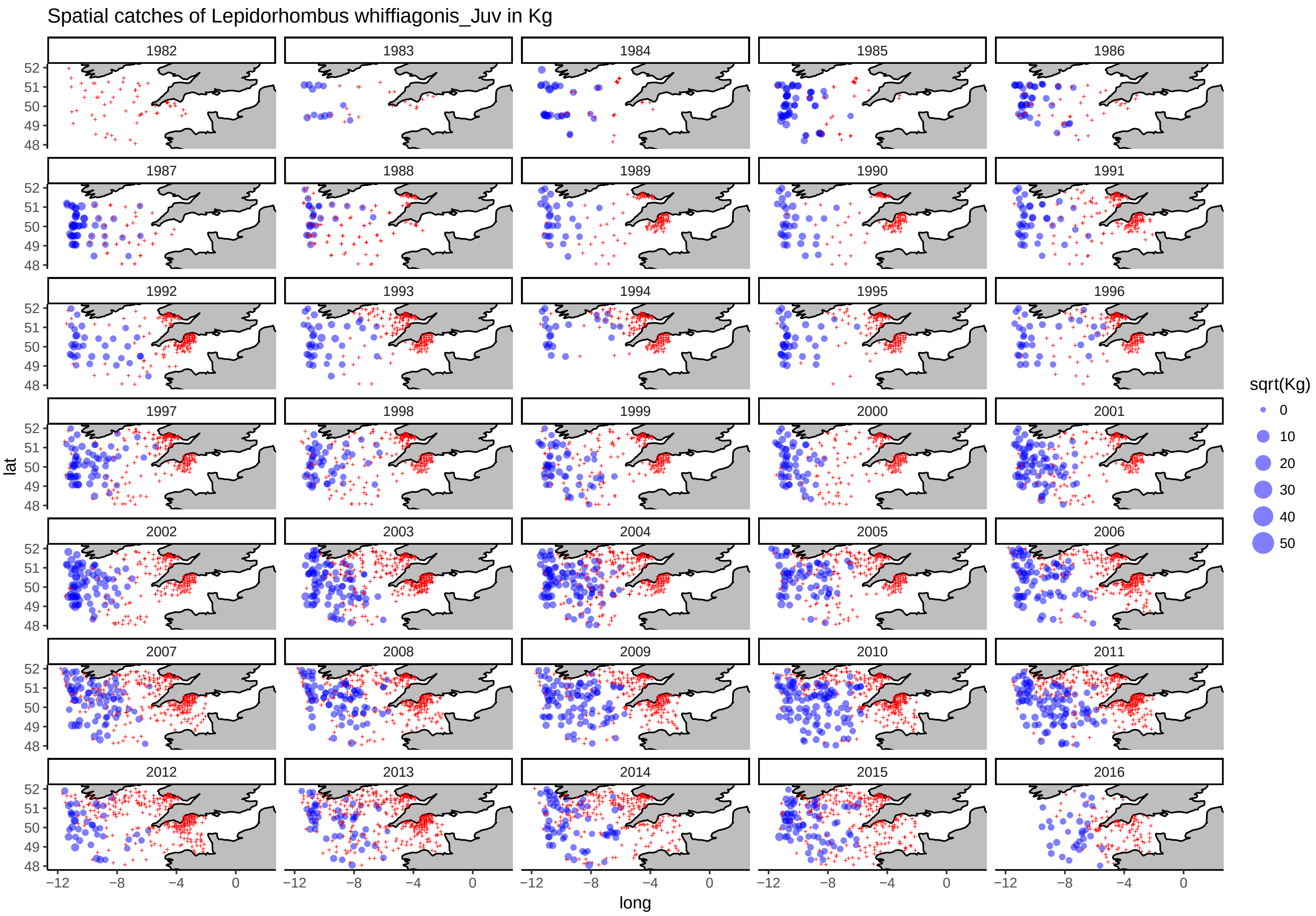

Spatial catches of Lepidorhombus whiffiagonis_Juv in Kg
1982
1983
1984
1985
1986
1987
1988
1989
1990
1991
1992
1993
1994
1995
1996
1997
1998
1999
2000
2001
2002
2003
2004
2005
2006
2007
2008
2009
2010
2011
2012
2013
2014
2015
2016
lat
52
51
50
49
48
long
−12
−8
−4
0
sqrt(Kg)
0
10
20
30
40
50

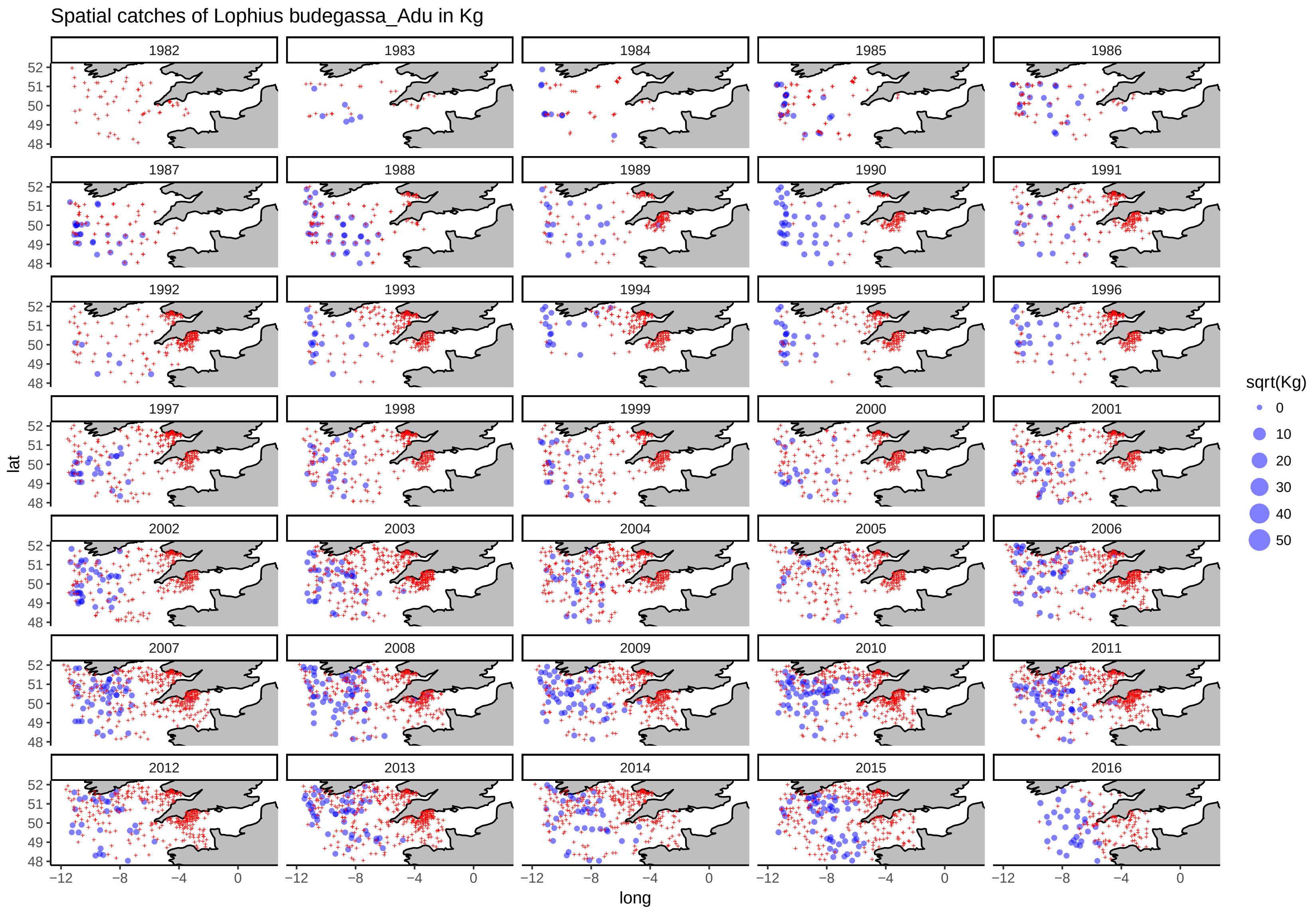
Spatial catches of Lophius budegassa_Adu in Kg
1982
1983
1984
1985
1986
1987
1988
1989
1990
1991
1992
1993
1994
1995
1996
1997
1998
1999
2000
2001
2002
2003
2004
2005
2006
2007
2008
2009
2010
2011
2012
2013
2014
2015
2016
lat
long
sqrt(Kg)
0
10
20
30
40
50

Spatial catches of Lophius budegassa_Juv in Kg

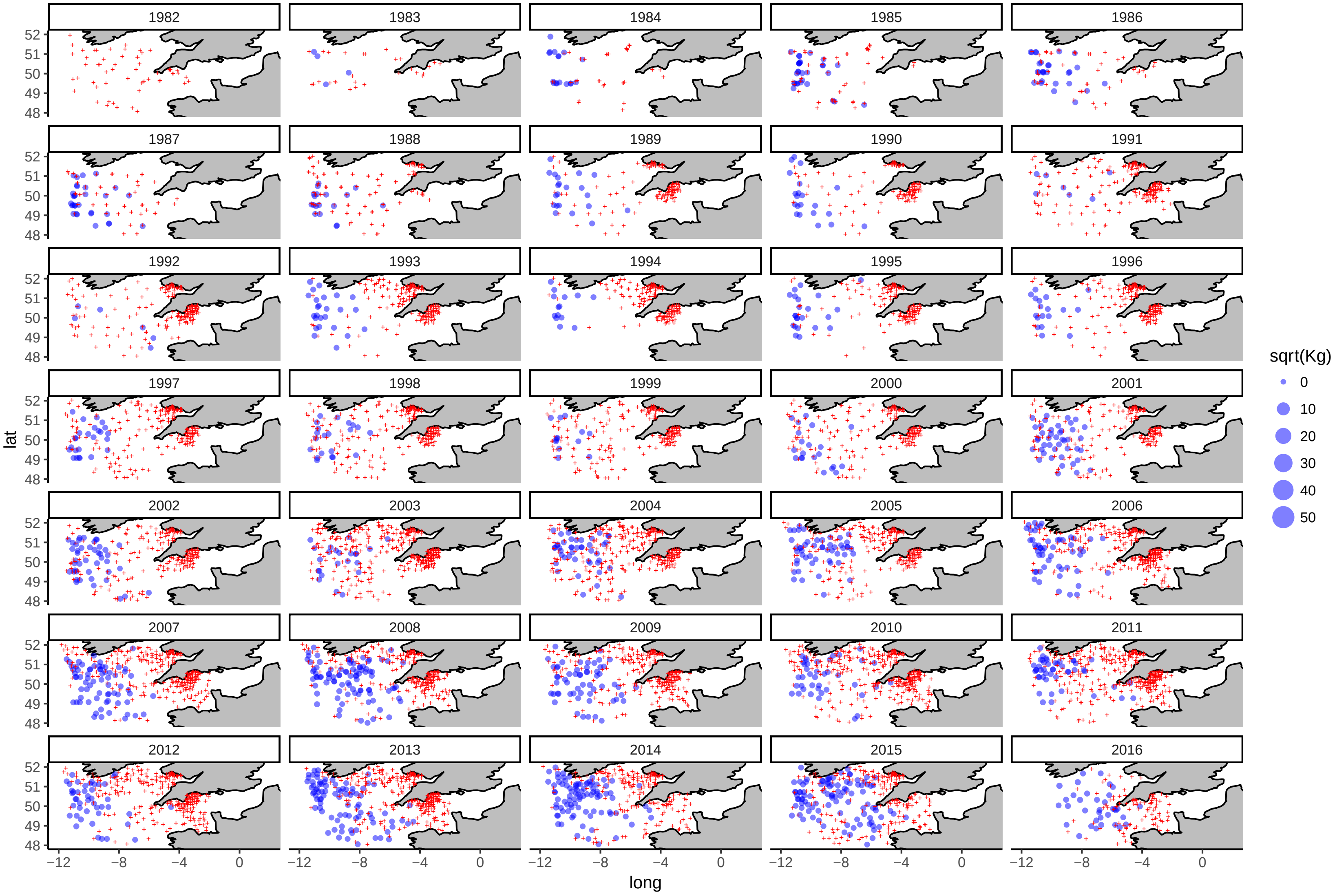

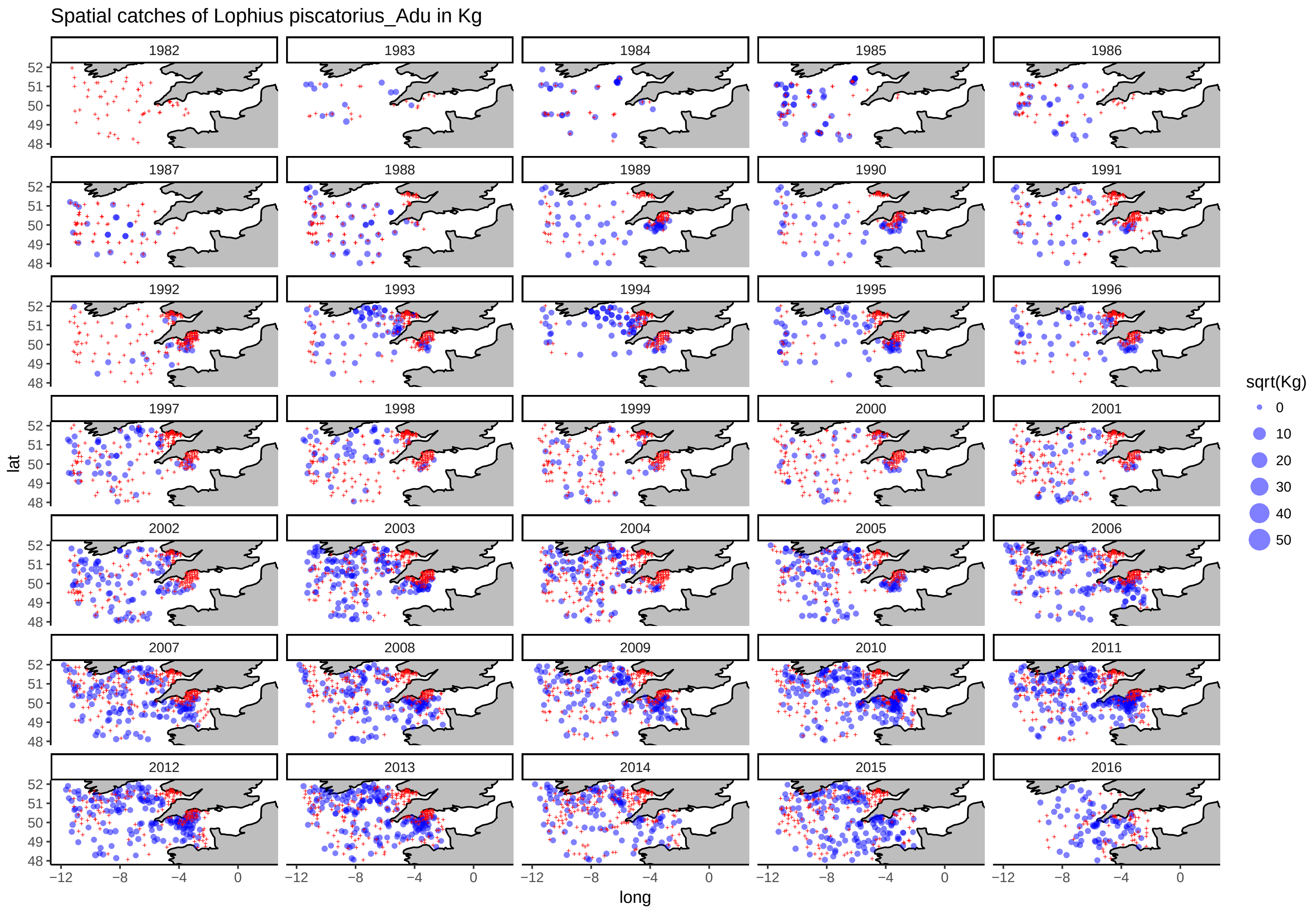
Spatial catches of Lophius piscatorius_Adu in Kg
1982
1983
1984
1985
1986
1987
1988
1989
1990
1991
1992
1993
1994
1995
1996
1997
1998
1999
2000
2001
2002
2003
2004
2005
2006
2007
2008
2009
2010
2011
2012
2013
2014
2015
2016
lat
52
51
50
49
48
long
−12
−8
−4
0
sqrt(Kg)
0
10
20
30
40
50

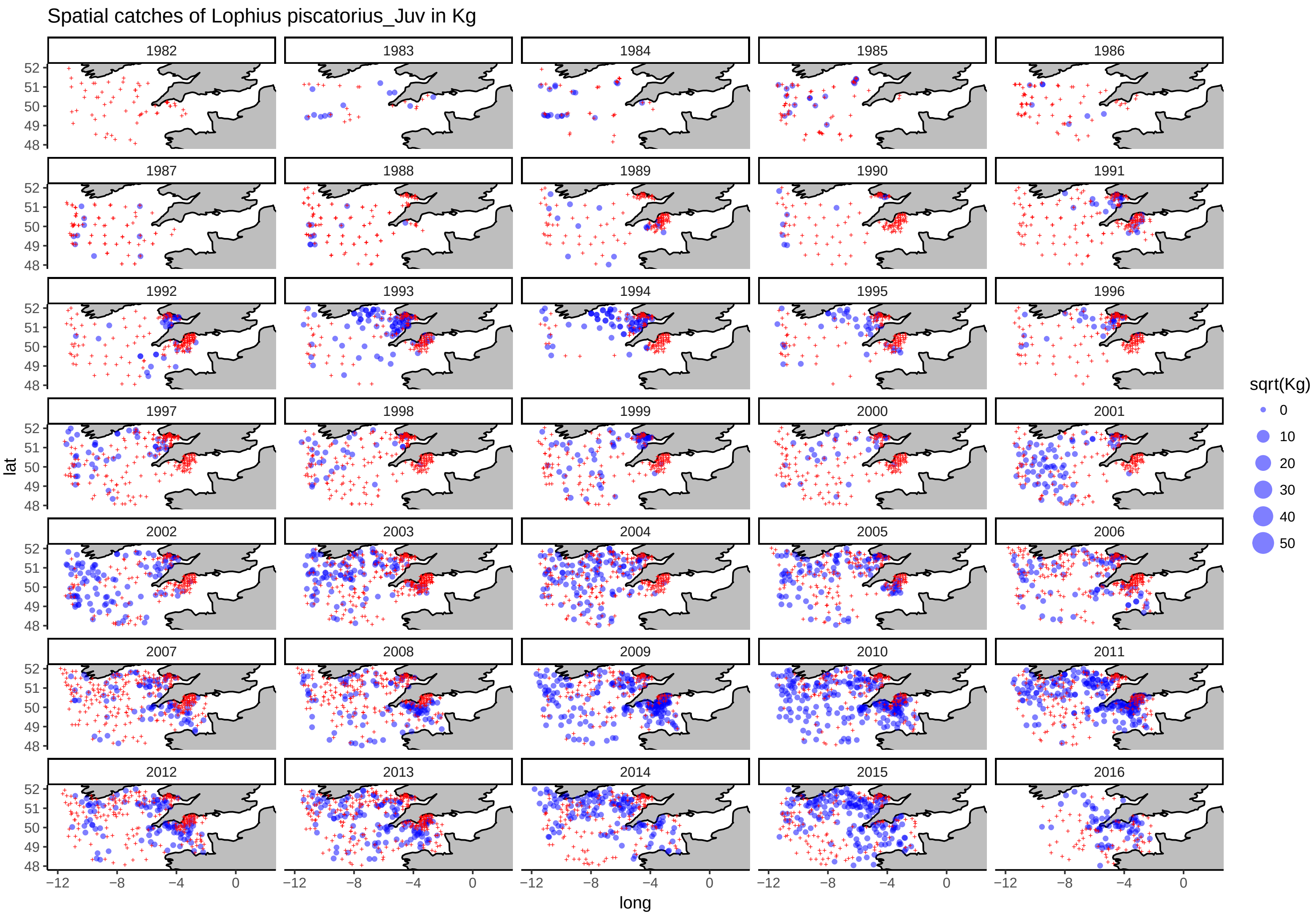

Spatial catches of Lophius piscatorius_Juv in Kg
1982
1983
1984
1985
1986
1987
1988
1989
1990
1991
1992
1993
1994
1995
1996
1997
1998
1999
2000
2001
2002
2003
2004
2005
2006
2007
2008
2009
2010
2011
2012
2013
2014
2015
2016
lat
52
51
50
49
48
long
−12
−8
−4
0
sqrt(Kg)
0
10
20
30
40
50

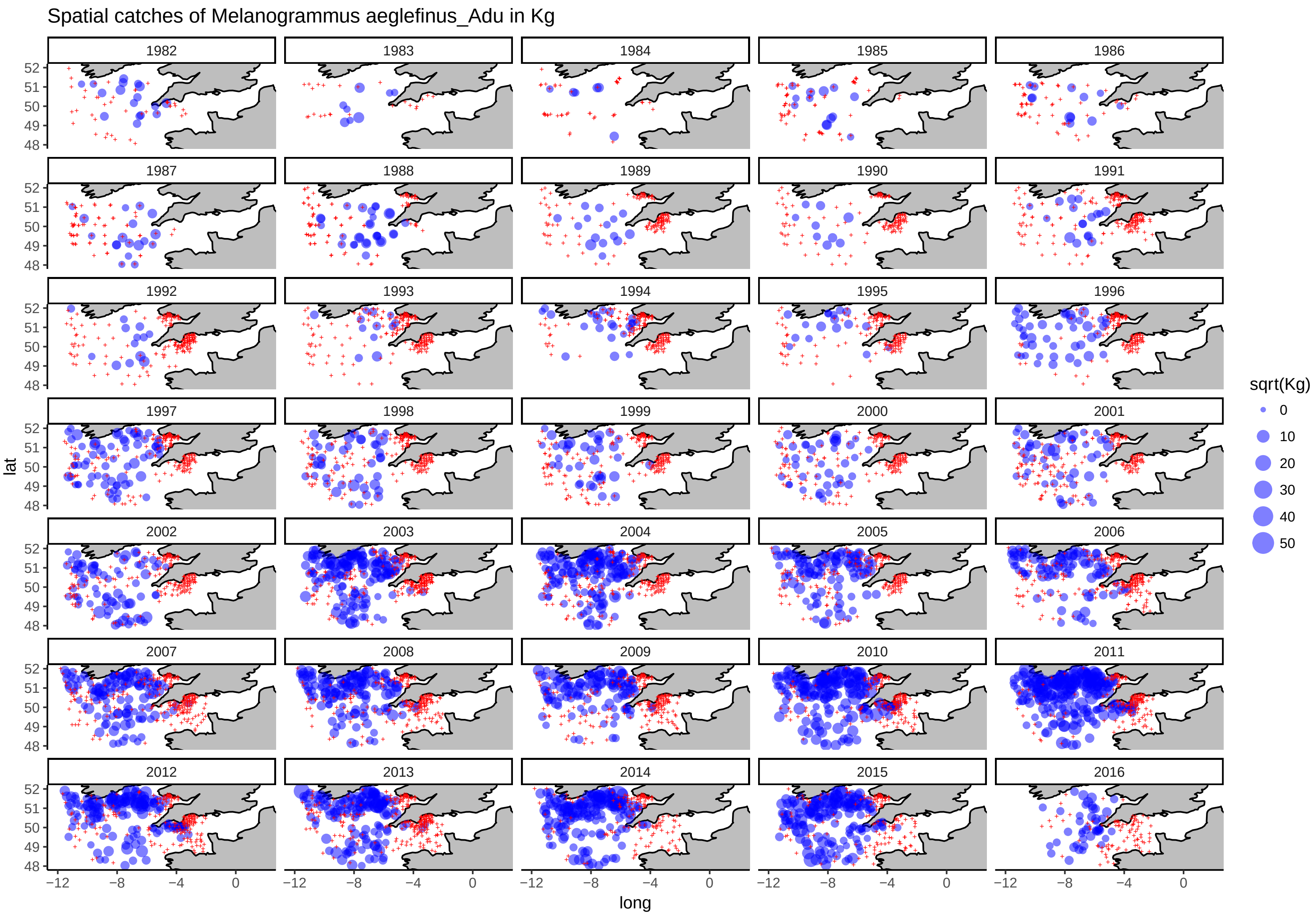
Spatial catches of Melanogrammus aeglefinus_Adu in Kg
1982
1983
1984
1985
1986
1987
1988
1989
1990
1991
1992
1993
1994
1995
1996
1997
1998
1999
2000
2001
2002
2003
2004
2005
2006
2007
2008
2009
2010
2011
2012
2013
2014
2015
2016
lat
52
51
50
49
48
long
−12
−8
−4
0
sqrt(Kg)
0
10
20
30
40
50

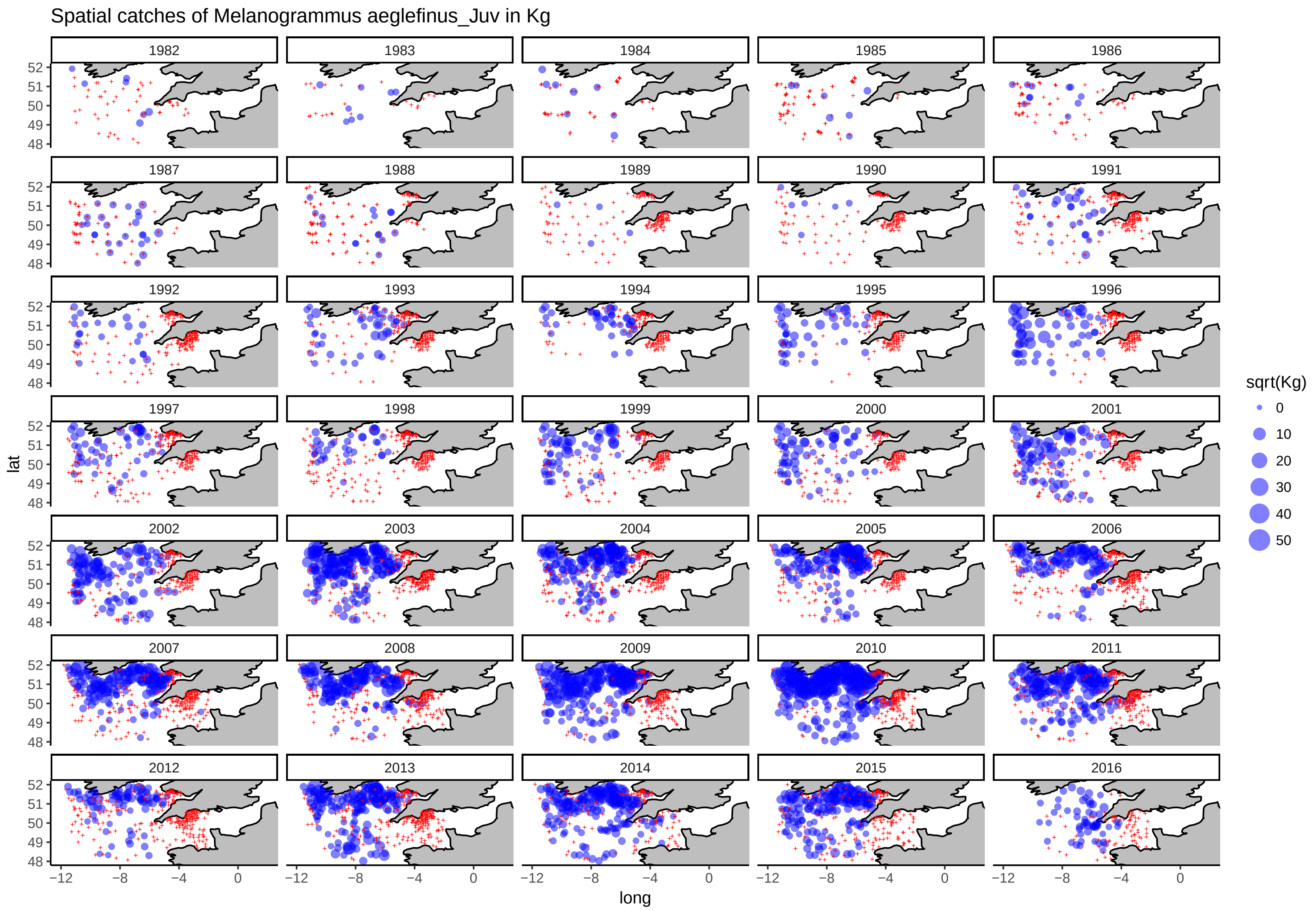
Spatial catches of Melanogrammus aeglefinus_Juv in Kg
1982
1983
1984
1985
1986
1987
1988
1989
1990
1991
1992
1993
1994
1995
1996
1997
1998
1999
2000
2001
2002
2003
2004
2005
2006
2007
2008
2009
2010
2011
2012
2013
2014
2015
2016
lat
52
51
50
49
48
long
−12
−8
−4
0
sqrt(Kg)
0
10
20
30
40
50

Spatial catches of Merlangius merlangus_Adu in Kg

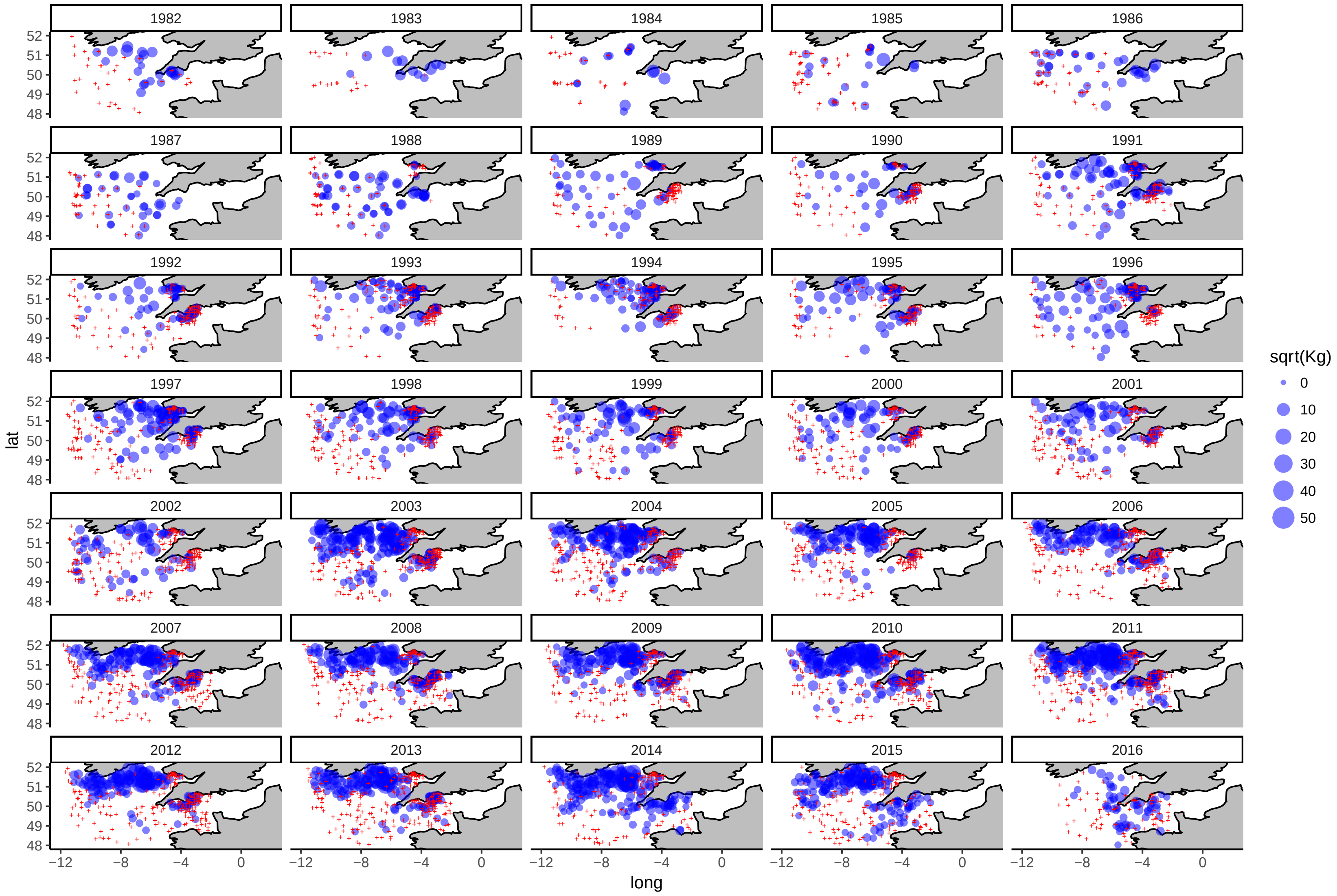

Spatial catches of Merlangius merlangus_Juv in Kg

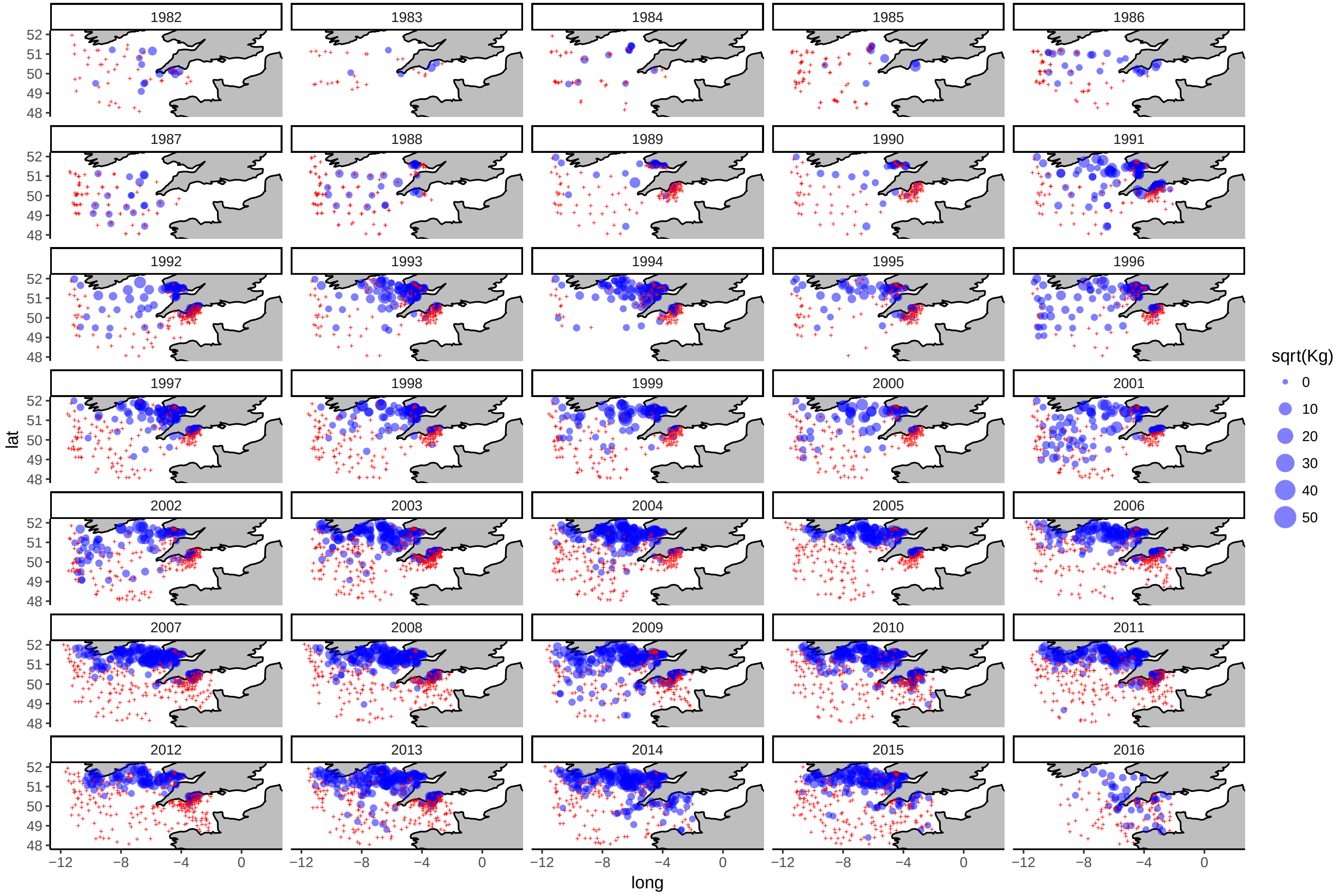

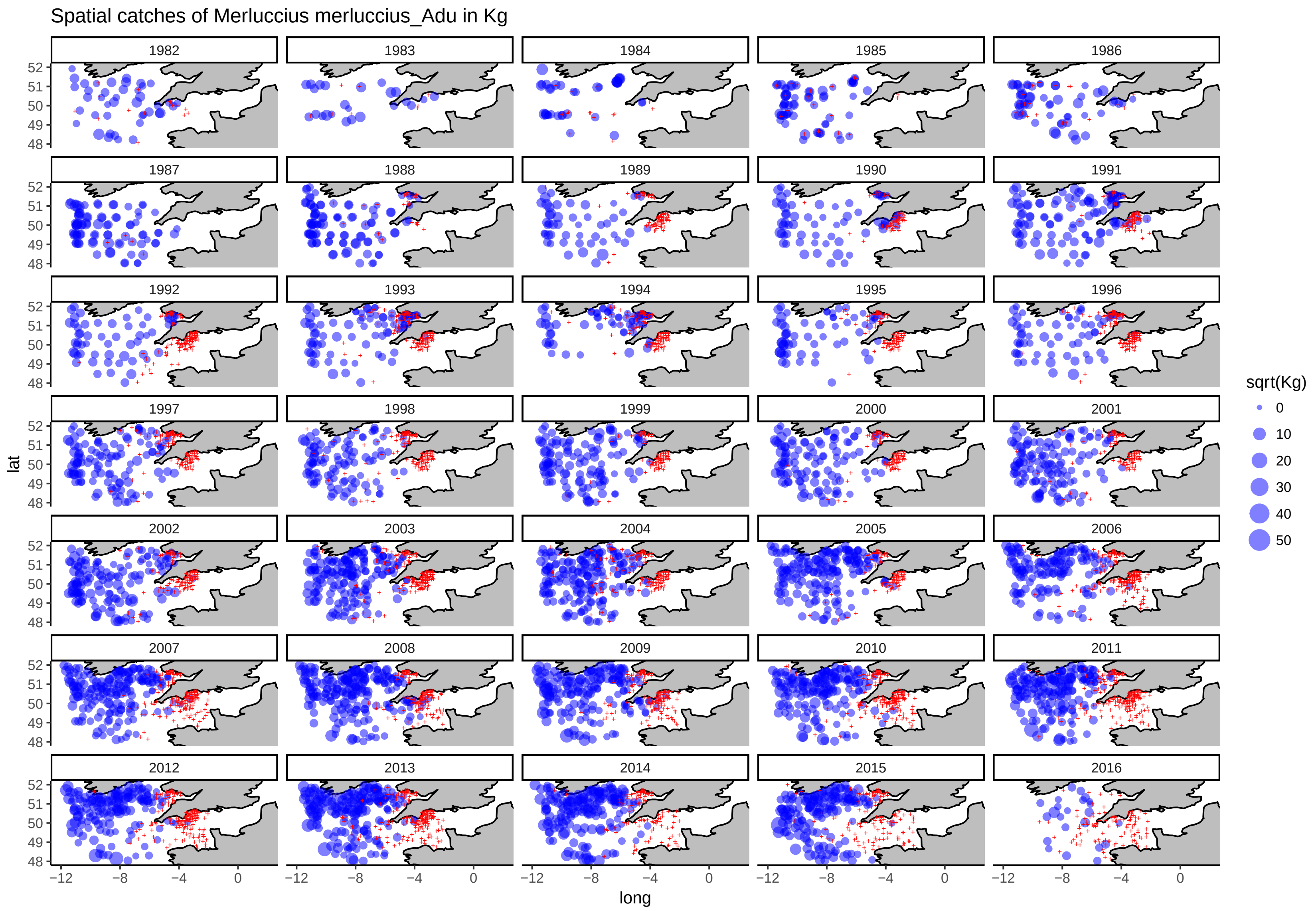

Spatial catches of Merluccius merluccius_Adu in Kg
1982
1983
1984
1985
1986
1987
1988
1989
1990
1991
1992
1993
1994
1995
1996
1997
1998
1999
2000
2001
2002
2003
2004
2005
2006
2007
2008
2009
2010
2011
2012
2013
2014
2015
2016
lat
52
51
50
49
48
long
−12
−8
−4
0
sqrt(Kg)
0
10
20
30
40
50

Spatial catches of Merluccius merluccius_Juv in Kg

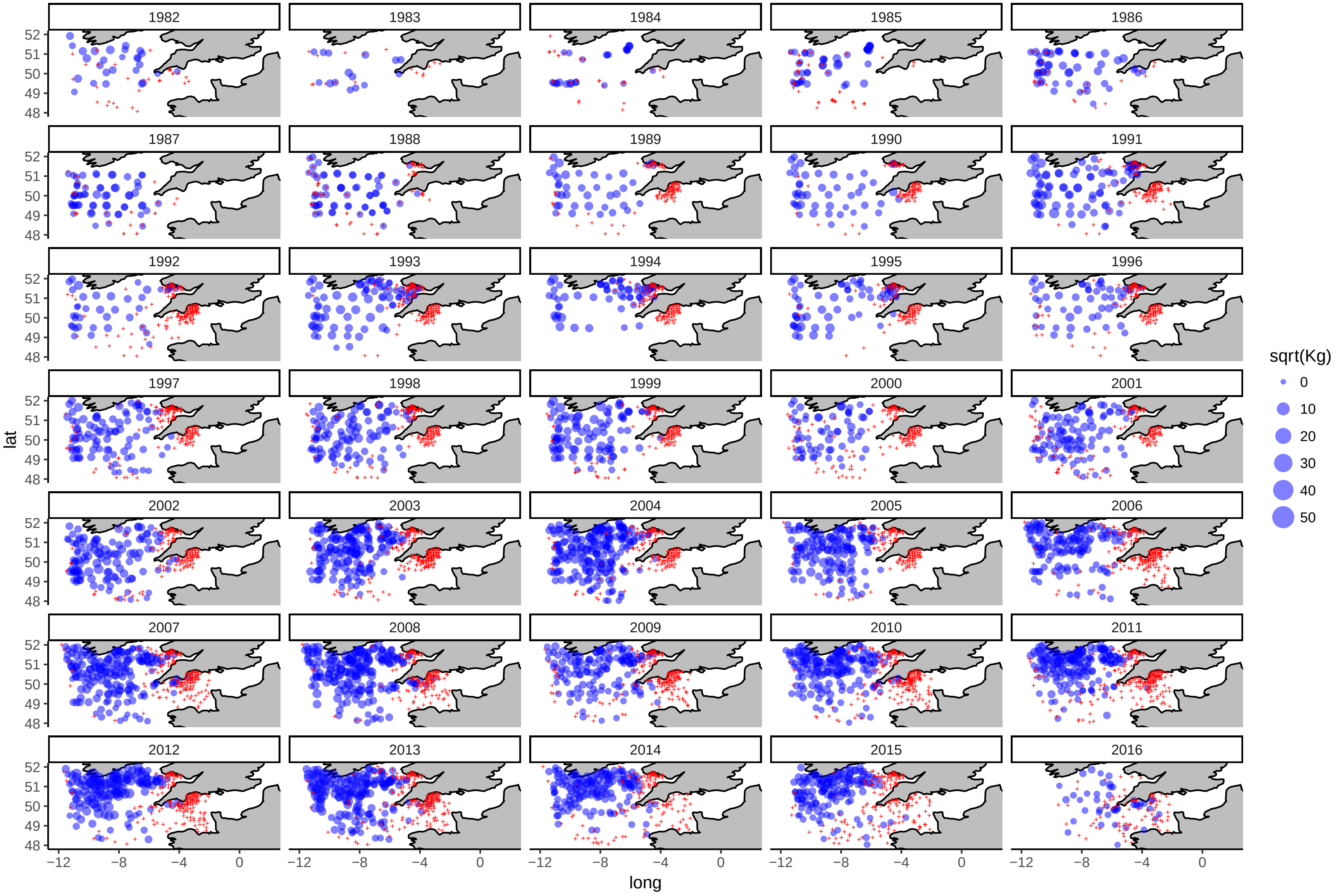

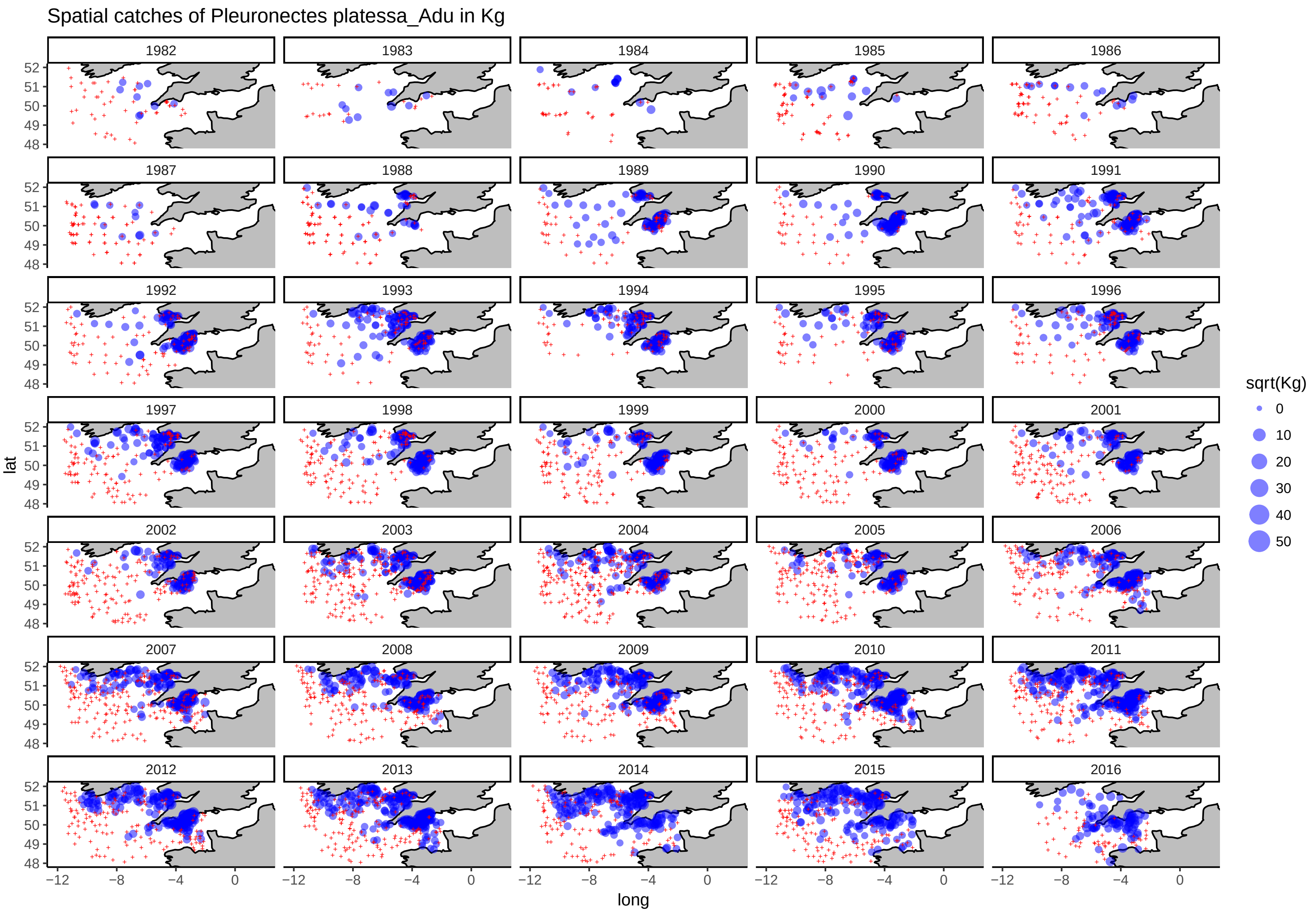

Spatial catches of Pleuronectes platessa_Adu in Kg
1982
1983
1984
1985
1986
1987
1988
1989
1990
1991
1992
1993
1994
1995
1996
1997
1998
1999
2000
2001
2002
2003
2004
2005
2006
2007
2008
2009
2010
2011
2012
2013
2014
2015
2016
lat
52
51
50
49
48
long
−12
−8
−4
0
sqrt(Kg)
0
10
20
30
40
50

Spatial catches of Pleuronectes platessa_Juv in Kg

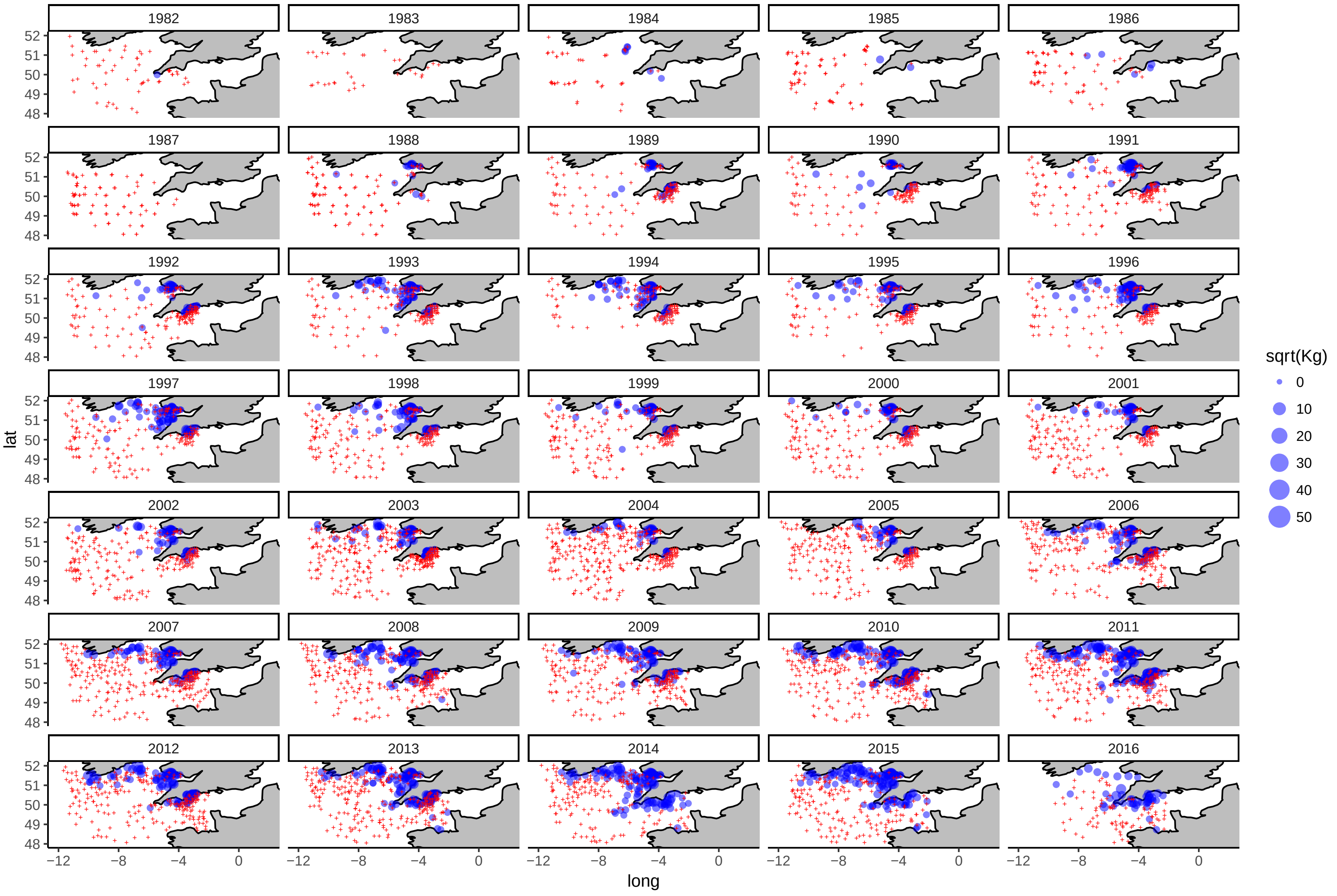

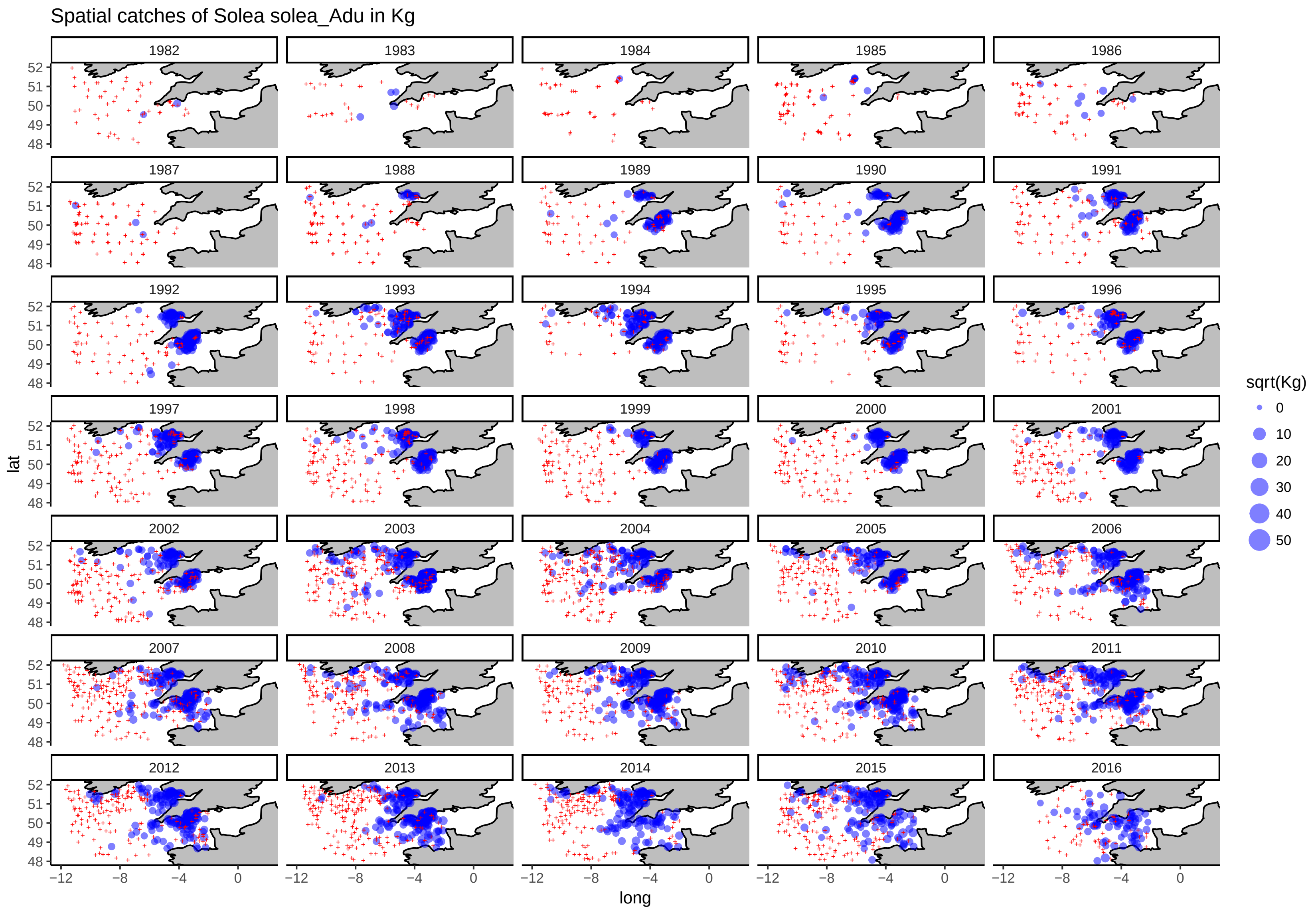

Spatial catches of Solea solea_Adu in Kg
1982
1983
1984
1985
1986
1987
1988
1989
1990
1991
1992
1993
1994
1995
1996
1997
1998
1999
2000
2001
2002
2003
2004
2005
2006
2007
2008
2009
2010
2011
2012
2013
2014
2015
2016
lat
52
51
50
49
48
long
−12
−8
−4
0
sqrt(Kg)
0
10
20
30
40
50

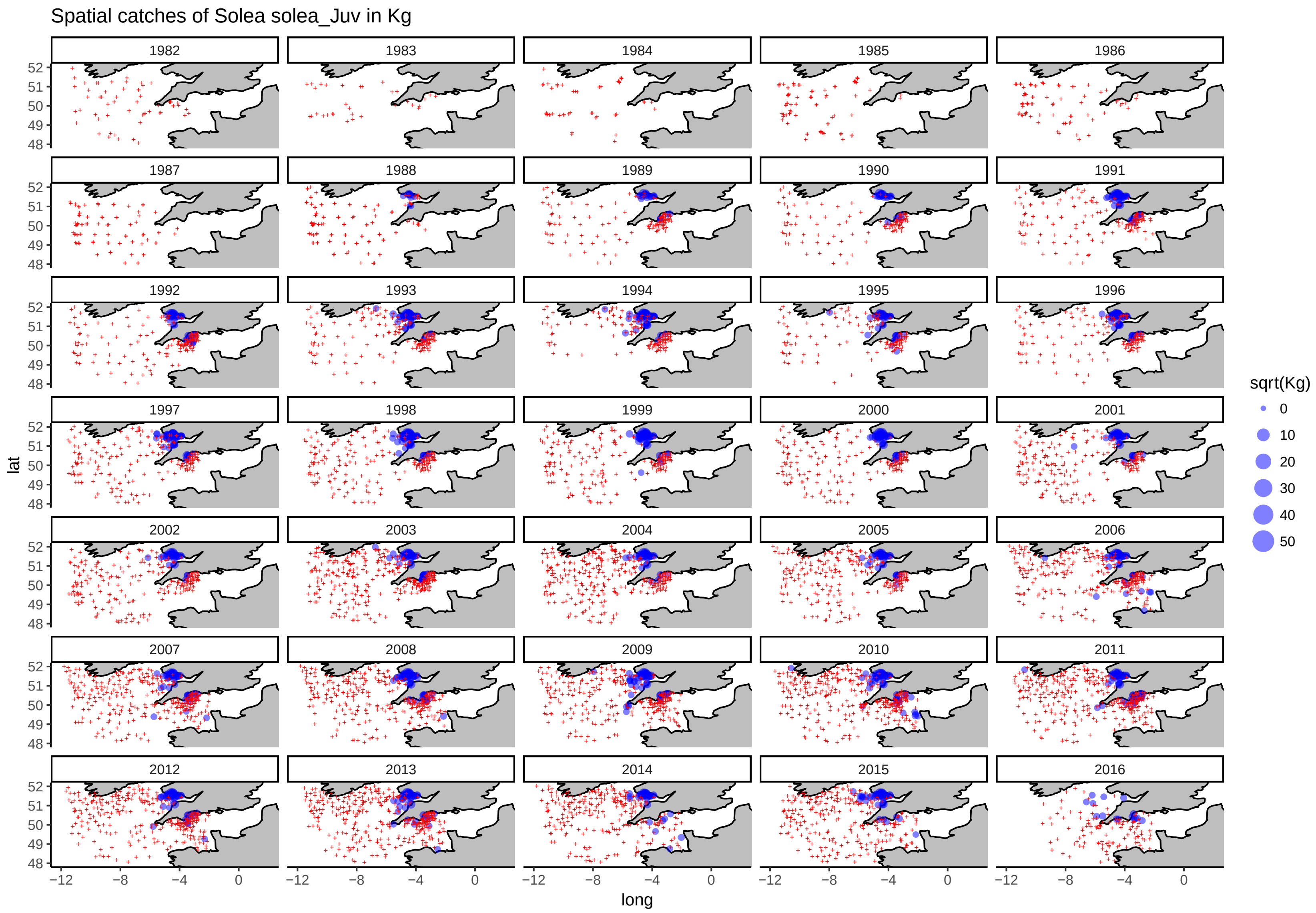
Spatial catches of Solea solea_Juv in Kg
1982
1983
1984
1985
1986
1987
1988
1989
1990
1991
1992
1993
1994
1995
1996
1997
1998
1999
2000
2001
2002
2003
2004
2005
2006
2007
2008
2009
2010
2011
2012
2013
2014
2015
2016
lat
52
51
50
49
48
long
−12
−8
−4
0
sqrt(Kg)
0
10
20
30
40
50

```r
Wt$HaulDur <- as.numeric(as.character(Wt$HaulDur))

cpue <- group_by(Wt, Survey, Year, Species) %>% summarise(q05 = quantile(Kg/HaulDur *
    60, prob = 0.05, na.rm = T), q50 = quantile(Kg/HaulDur *
    60, prob = 0.5, na.rm = T), mean = mean(Kg/HaulDur * 60,
    na.rm = T), q95 = quantile(Kg/HaulDur * 60, prob = 0.95,
    na.rm = T))

print(ggplot(cpue, aes(x = Year, y = mean)) + geom_line(aes(group = Survey,
    colour = Survey)) + facet_wrap(~Species, ncol = 2, scale = "free_y") +
    theme(axis.text.x = element_text(angle = -90)) + ylab("Kg per hour tow") +
    xlab("") + ggtitle("CPUE (Kg per hour tow)"))
```

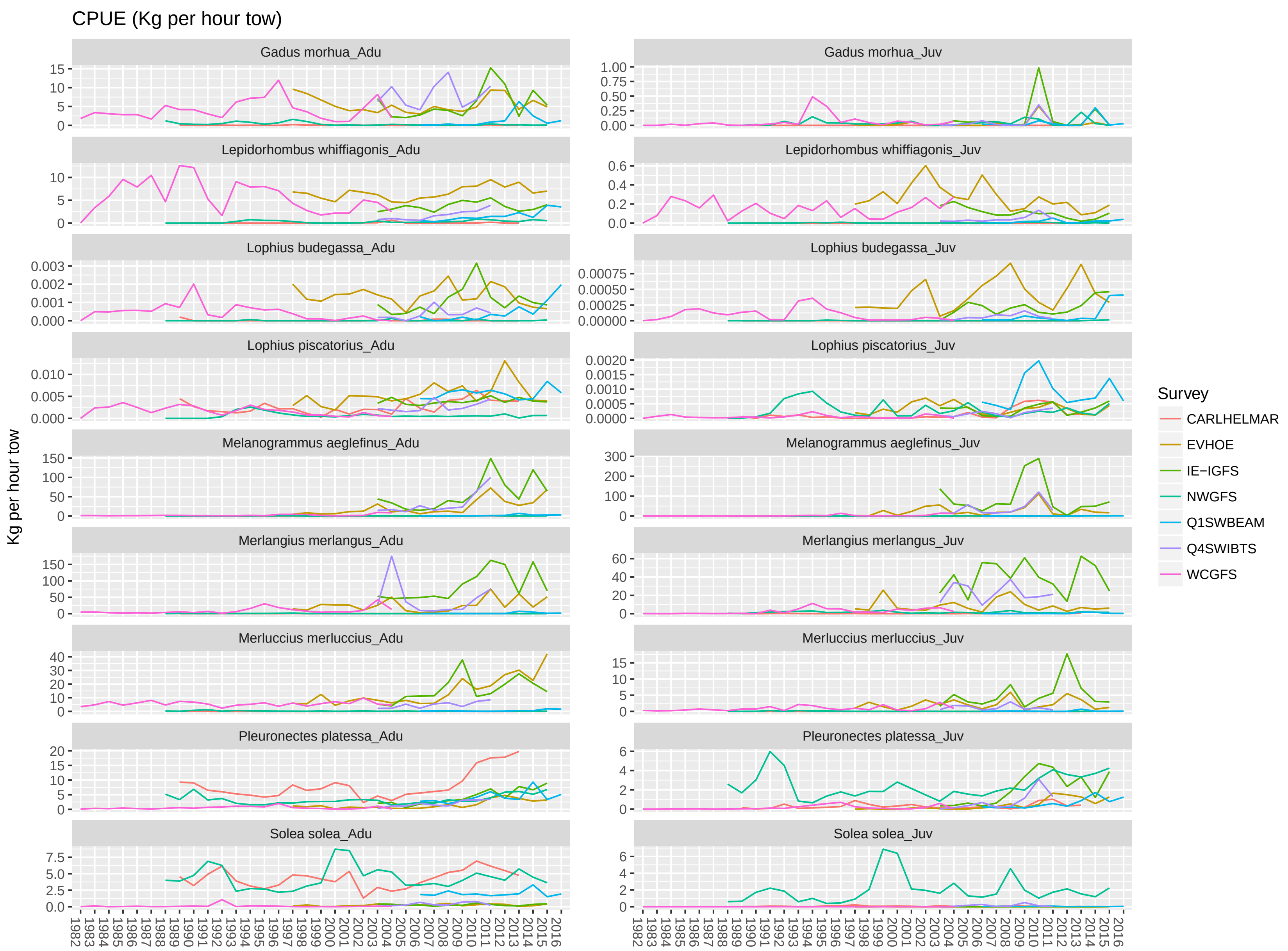
CPUE (Kg per hour tow)
Kg per hour tow
Gadus morhua_Adu
Gadus morhua_Juv
Lepidorhombus whiffiagonis_Adu
Lepidorhombus whiffiagonis_Juv
Lophius budegassa_Adu
Lophius budegassa_Juv
Lophius piscatorius_Adu
Lophius piscatorius_Juv
Melanogrammus aeglefinus_Adu
Melanogrammus aeglefinus_Juv
Merlangius merlangus_Adu
Merlangius merlangus_Juv
Merluccius merluccius_Adu
Merluccius merluccius_Juv
Pleuronectes platessa_Adu
Pleuronectes platessa_Juv
Solea solea_Adu
Solea solea_Juv
Survey
CARLHELMAR
EVHOE
IE−IGFS
NWGFS
Q1SWBEAM
Q4SWIBTS
WCGFS

```r
cpsa <- group_by(Wt, Survey, Year, Species) %>% summarise(q05 = quantile(Kg/SweptArea,
    prob = 0.05, na.rm = T), q50 = quantile(Kg/SweptArea, prob = 0.5,
    na.rm = T), mean = mean(Kg/SweptArea, na.rm = T), q95 = quantile(Kg/SweptArea,
    prob = 0.95, na.rm = T))

print(ggplot(cpsa, aes(x = Year, y = mean)) + geom_line(aes(group = Survey,
    colour = Survey)) + facet_wrap(~Species, ncol = 2, scale = "free_y") +
    theme(axis.text.x = element_text(angle = -90)) + ylab("Density (catch per km2 swept)") +
    xlab("") + ggtitle("CPUE (Catch per km2 swept area)"))
```

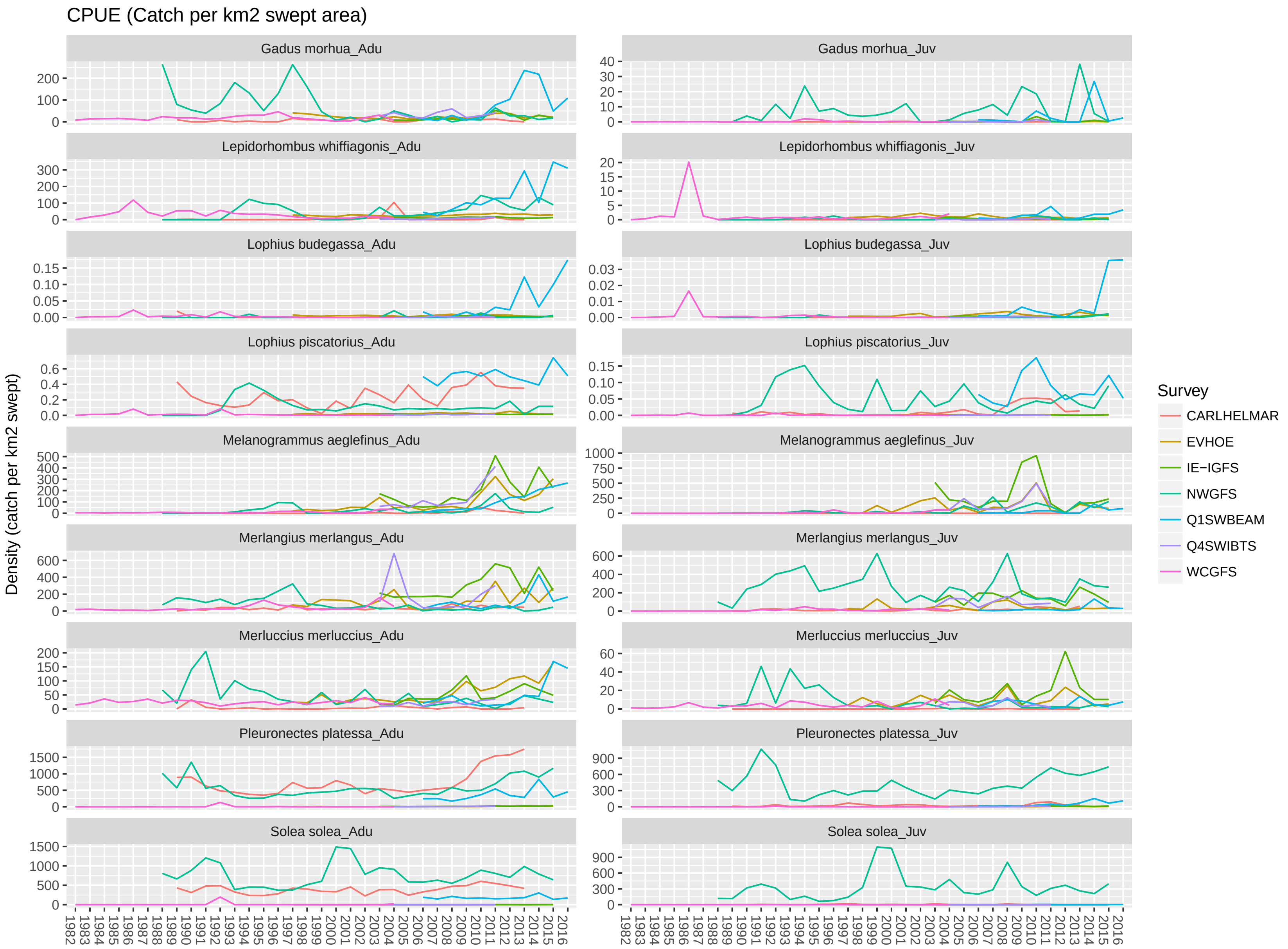
CPUE (Catch per km2 swept area)
Density (catch per km2 swept)
Gadus morhua_Adu
Gadus morhua_Juv
Lepidorhombus whiffiagonis_Adu
Lepidorhombus whiffiagonis_Juv
Lophius budegassa_Adu
Lophius budegassa_Juv
Lophius piscatorius_Adu
Lophius piscatorius_Juv
Melanogrammus aeglefinus_Adu
Melanogrammus aeglefinus_Juv
Merlangius merlangus_Adu
Merlangius merlangus_Juv
Merluccius merluccius_Adu
Merluccius merluccius_Juv
Pleuronectes platessa_Adu
Pleuronectes platessa_Juv
Solea solea_Adu
Solea solea_Juv
Survey
CARLHELMAR
EVHOE
IE−IGFS
NWGFS
Q1SWBEAM
Q4SWIBTS
WCGFS

```r
## Catchs per survey, per year

Wt_Sur <- group_by(Wt, Survey, Species) %>% summarise(wt = sum(Kg))

Wt_Sur[Wt_Sur$wt == 0, ]


## Source: local data frame [2 x 3]
## Groups: Survey [1]
##
##       Survey                Species    wt
##       <fctr>                 <fctr> <dbl>
## 1 CARLHELMAR      Gadus morhua_Juv     0
## 2 CARLHELMAR Lophius budegassa_Juv     0


Wt_Sur$wt[Wt_Sur$wt == 0] <- NA

print(ggplot(Wt_Sur, aes(x = Survey, y = Species)) + geom_point(aes(size = sqrt(wt))) +
    theme_classic() + ggtitle("Catches of each species per survey"))
```

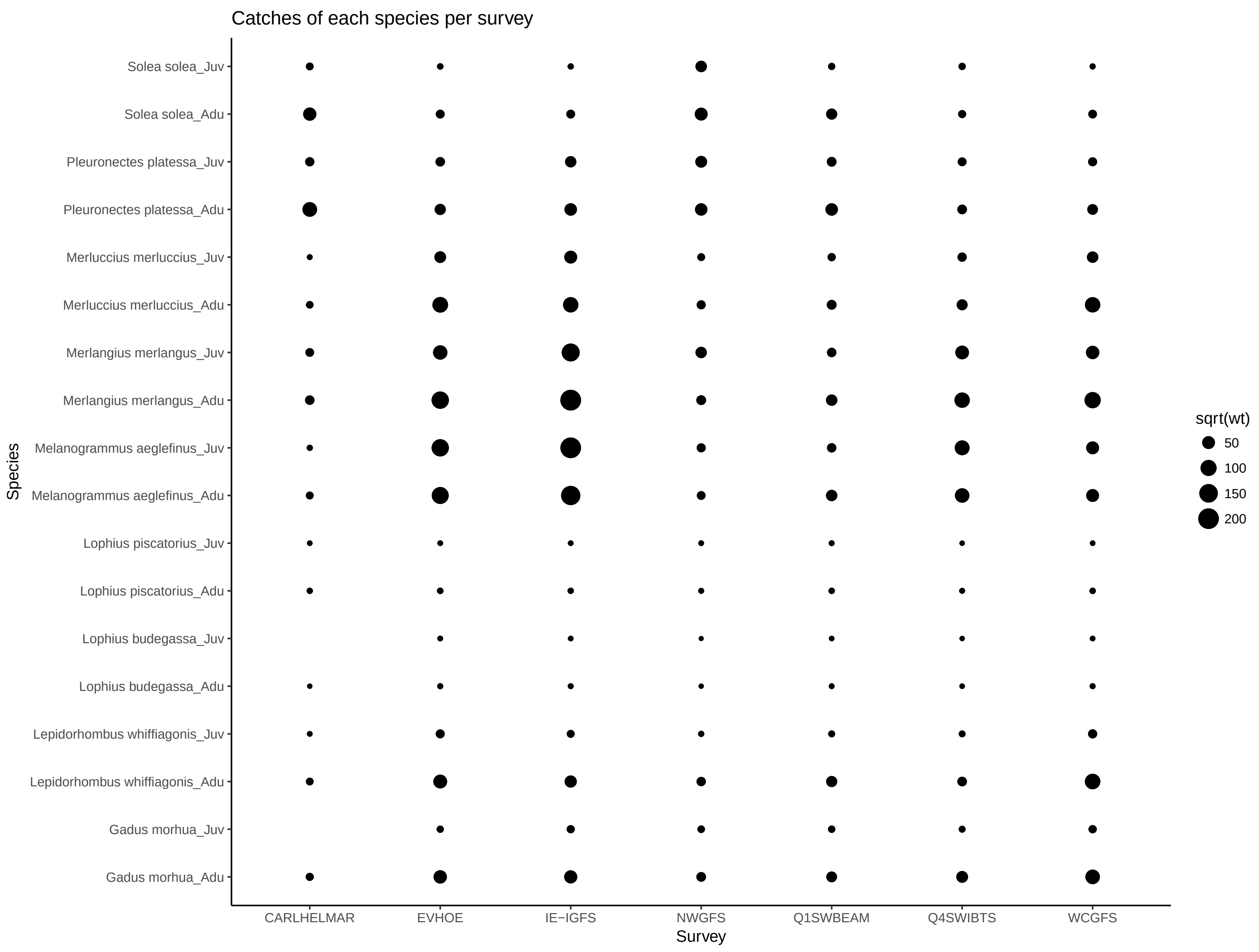

Catches of each species per survey
Species
Solea solea_Juv
Solea solea_Adu
Pleuronectes platessa_Juv
Pleuronectes platessa_Adu
Merluccius merluccius_Juv
Merluccius merluccius_Adu
Merlangius merlangus_Juv
Merlangius merlangus_Adu
Melanogrammus aeglefinus_Juv
Melanogrammus aeglefinus_Adu
Lophius piscatorius_Juv
Lophius piscatorius_Adu
Lophius budegassa_Juv
Lophius budegassa_Adu
Lepidorhombus whiffiagonis_Juv
Lepidorhombus whiffiagonis_Adu
Gadus morhua_Juv
Gadus morhua_Adu
CARLHELMAR
EVHOE
IE−IGFS
NWGFS
Q1SWBEAM
Q4SWIBTS
WCGFS
Survey
sqrt(wt)
50
100
150
200

It's apparent from the information that the CARLHELMAR survey area in the Western Channel sees little catch of the gadoid species. This is perhaps unsurprising given its designed as a flatfish survey.

The WCGFS, EVHOE, IE-IGFS and Q4SWIBTS show reasonable consistency with each other in terms of CPUE trends for cod, though the WCGFS caught less haddock and whiting.

4.3 Conclusion on survey availability

Having reviewed the available survey data, coverage prior to 1992 was patchy and incomplete and the CARLHELMAR and NWGFS surveys are focused on flatfish catches, with little information on gadoid species. Therefore it will be important to check model diagnostics to entire the characteristics are being treated appropriately. However, all the data will be kept for the first runs.

5 Habitat covariates

There is also the possibility to include habitat covariates in the model. In order to explore this, two datasets were downloaded:

- EU Sea Map Atlantic Habitat Classifications (from http://www.emodnet-seabedhabitats.eu/) which provides a substrate classification (e.g. rocky, sandy etc..) for the Celtic Sea area.
- Bathymetry data (from http://www.emodnet-hydrography.eu/ which provides water depth.

The following function is used to assign the correct habitat location to the knot locations generated by the VAST model.

```
HabAssignFunc <- function(Kmeans = NULL, zone = 29, locationHabMap = NULL,
    nameHabMap = NULL) {
    library(rgdal)
    library(VAST)
    # Create a dataframe of the knots
    DF <- data.frame(X = Kmeans$centers[, "E_km"], Y = Kmeans$centers[,
        "N_km"])
    attr(DF, "projection") = "UTM"
    attr(DF, "zone") <- zone
```

```r
    LLs <- PBSmapping::convUL(DF)

    HabMap <- readOGR(dsn = file.path(locationHabMap), layer = nameHabMap)

    # joint the spatial points..
    LLs <- SpatialPoints(LLs)
    proj4string(LLs) <- CRS("+proj=longlat +datum=WGS84 +no_defs +ellps=WGS84 +towgs84=0,0,

    join <- over(x = LLs, y = HabMap)

    LLs <- SpatialPointsDataFrame(LLs, join)
    KmeanHab <- data.frame(Habitat = LLs$substrate)

    return(KmeanHab)

}
```
