# Supplementary material for: Spatial separation of catches in highly mixed fisheries
Source: Sci Rep. 2018 Sep 17;8:13886. doi: 10.1038/s41598-018-31881-w (PMC6141535; doi:10.1038/s41598-018-31881-w)
Supplement: Supplementary file 2 — Supplementary Tables and Figures [file 41598_2018_31881_MOESM2_ESM.pdf]

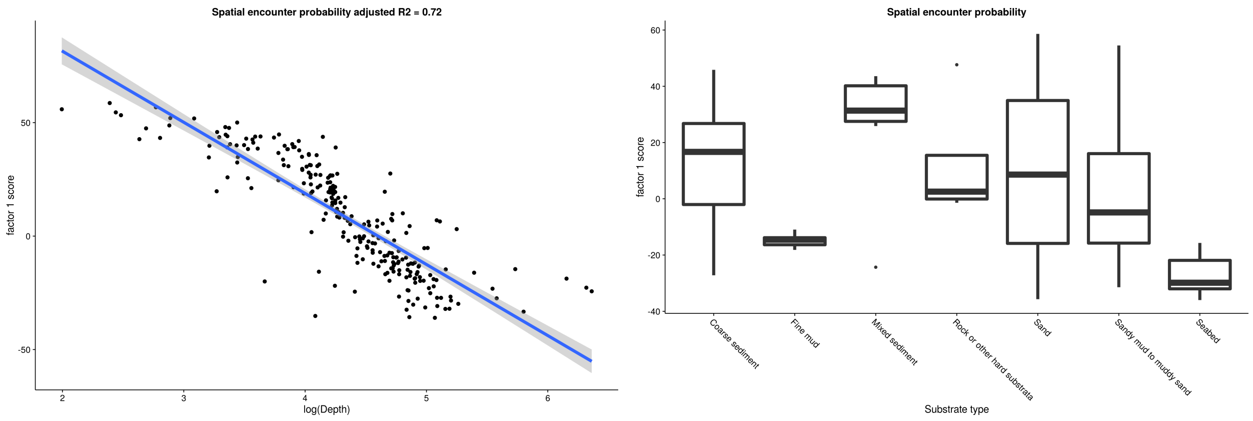

Figure S1: Left: Average spatial encounter probability factor 1 values correlated against; Left - Depth, Right - substrate type.

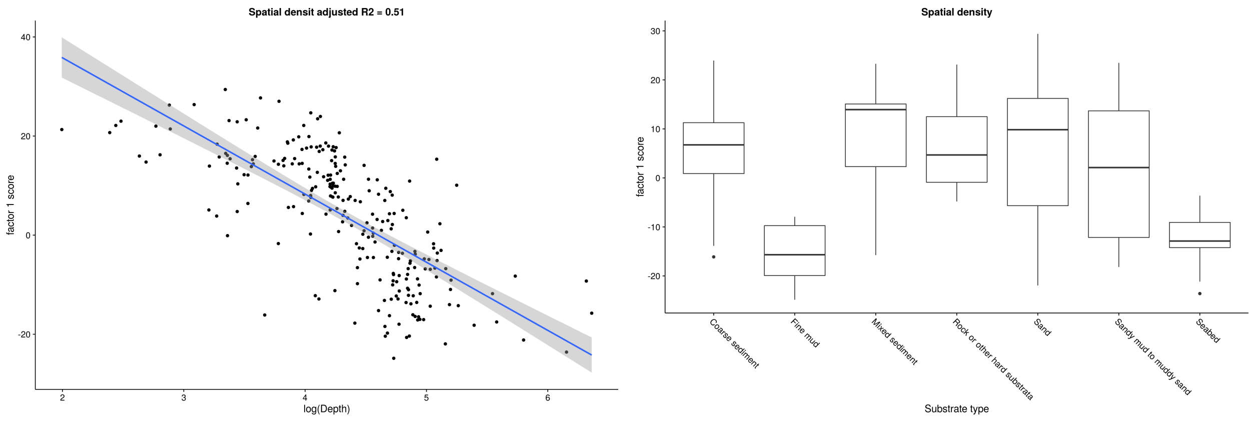

Figure S2: Left: Average spatial positive density factor 1 values correlated against; Left- Depth, Right- substrate type.

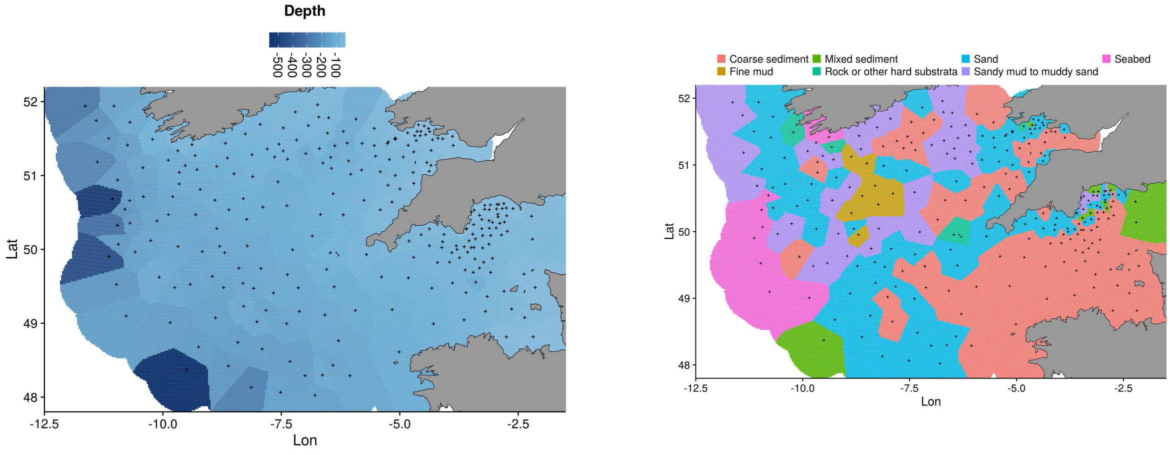

Figure S3: Left: Depth, Right: Substrate assigned to each spatial knot.

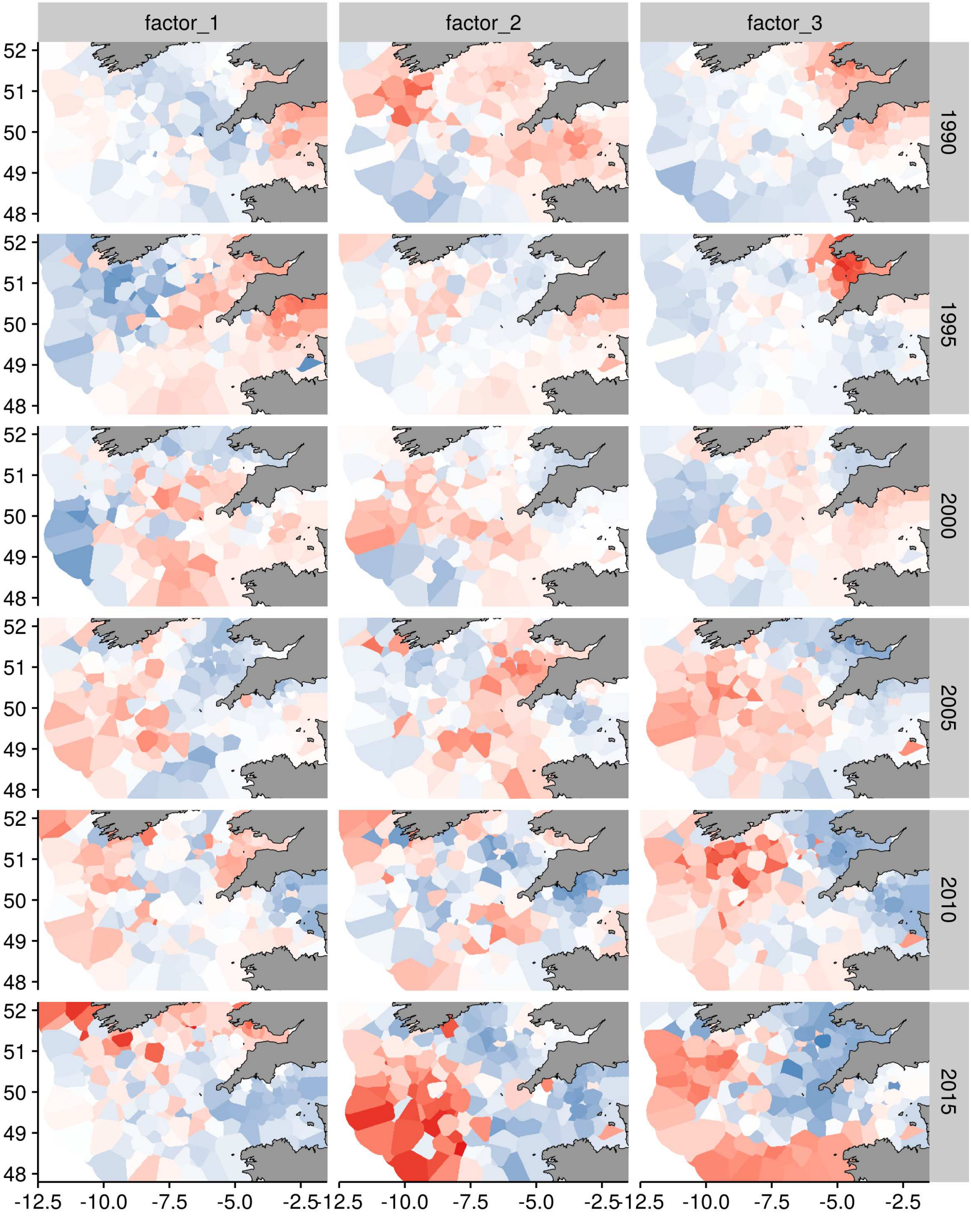

Figure S4: Spatial Loadings for first three factors every five years for spatio-temporal encounter probability.

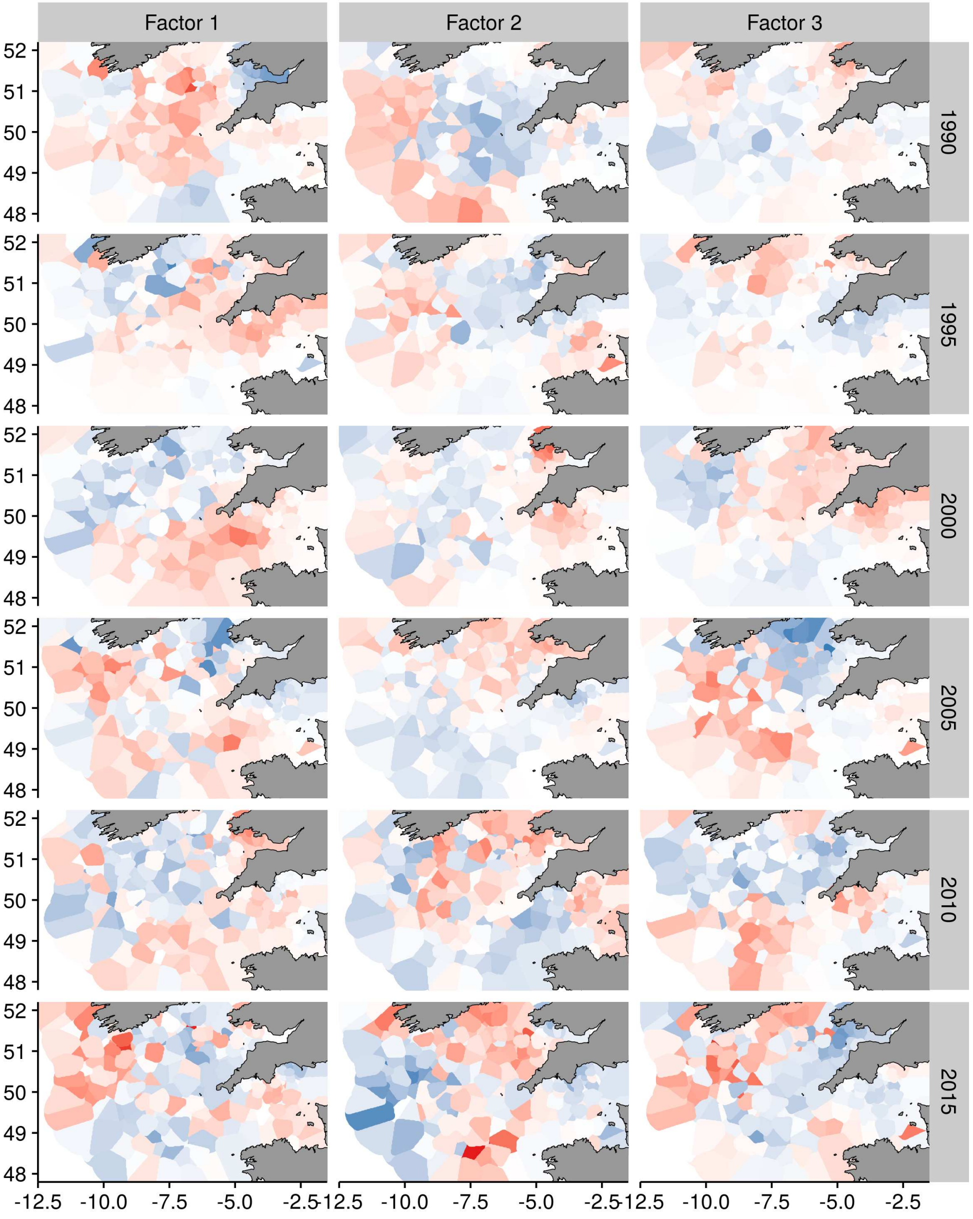

Figure S5: Spatial Loadings for first three factors every five years for spatio-temporal density.

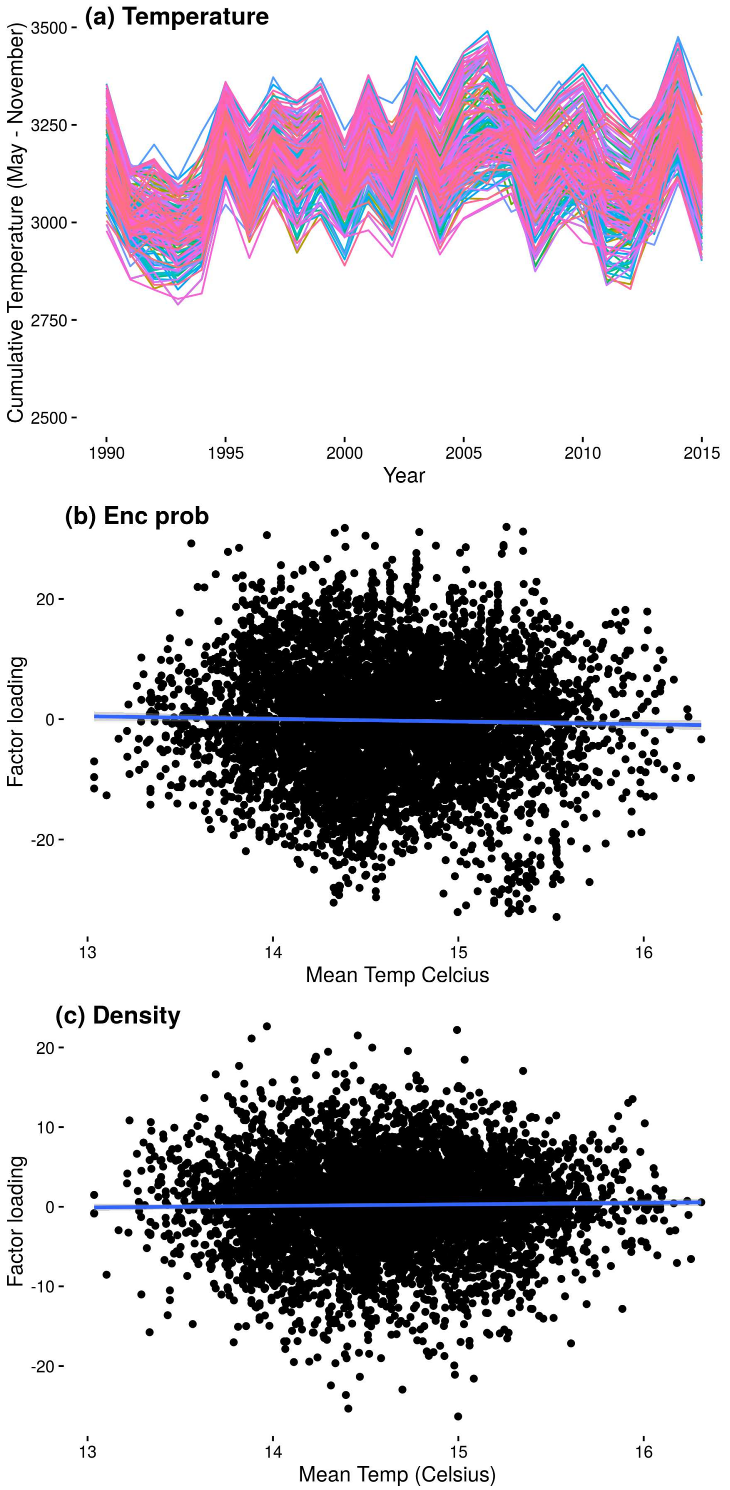

Figure S6: Association of temperature and knots (individual lines; top) with Spatio-temporal factor loadings for encounter probability (middle) and density (bottom).

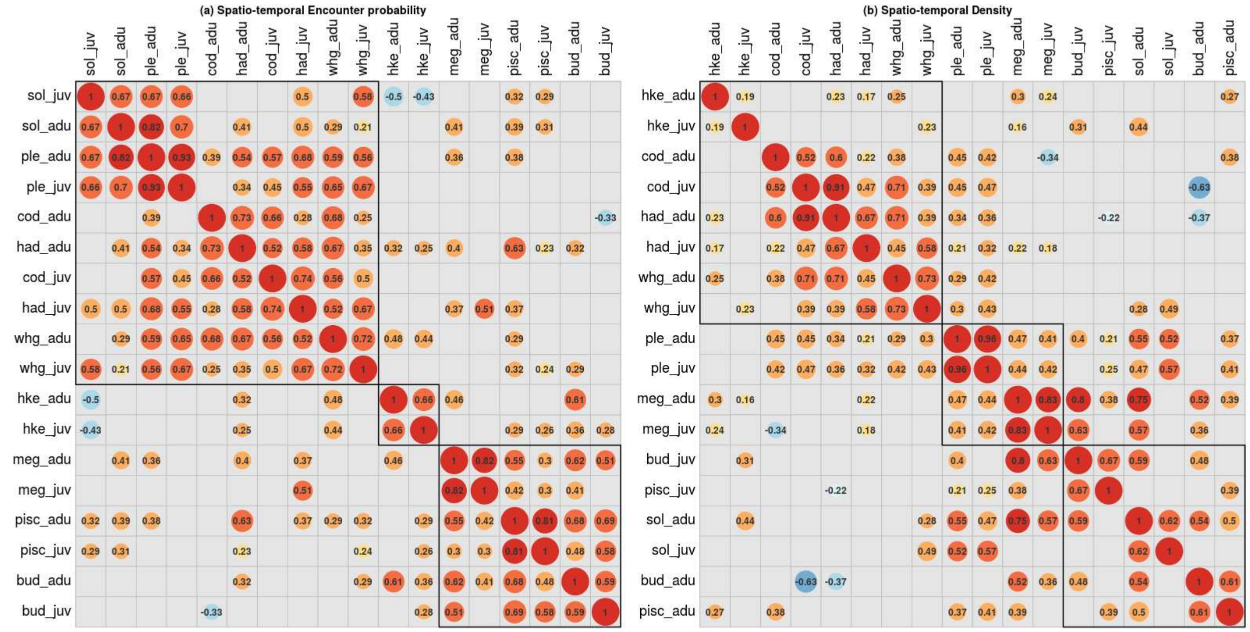

Figure S7: Inter-species correlations for (a) spatio-temporal encounter probability and b) spatio-temporal density. Species are clustered into three groups based on a hierarchical clustering method with non-significant correlations (the Confidence Interval [± 1.96 * SEs] spanned zero) left blank.

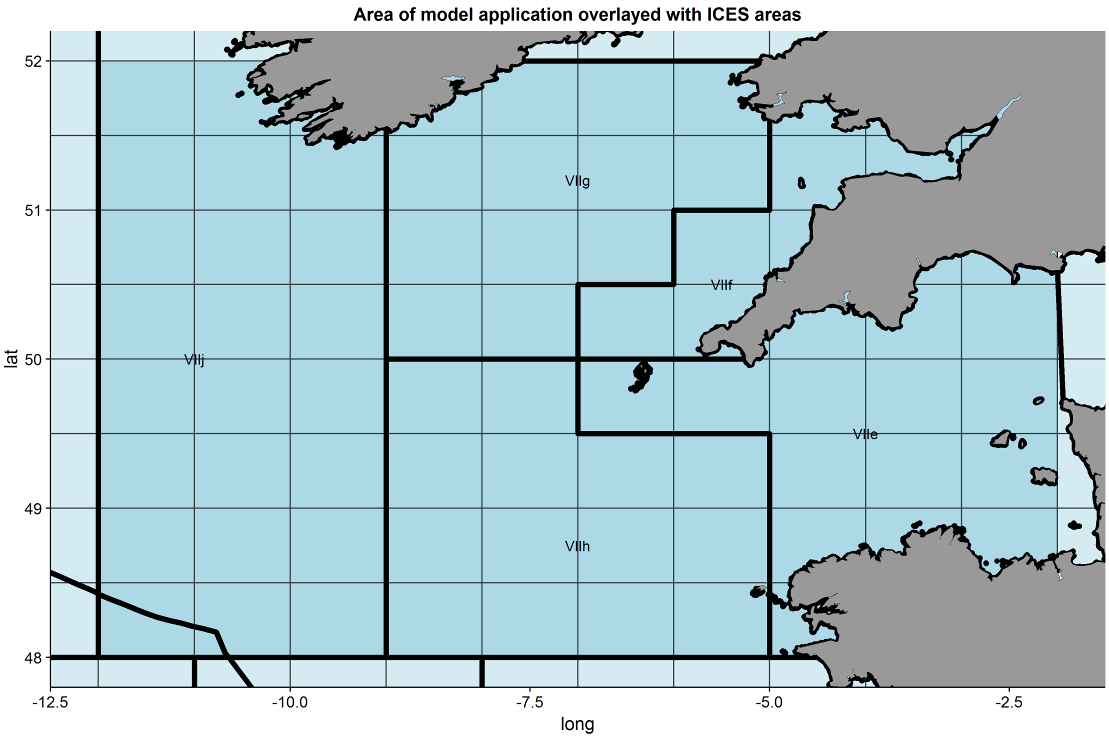

Figure S8: Spatial bounds of case study area.

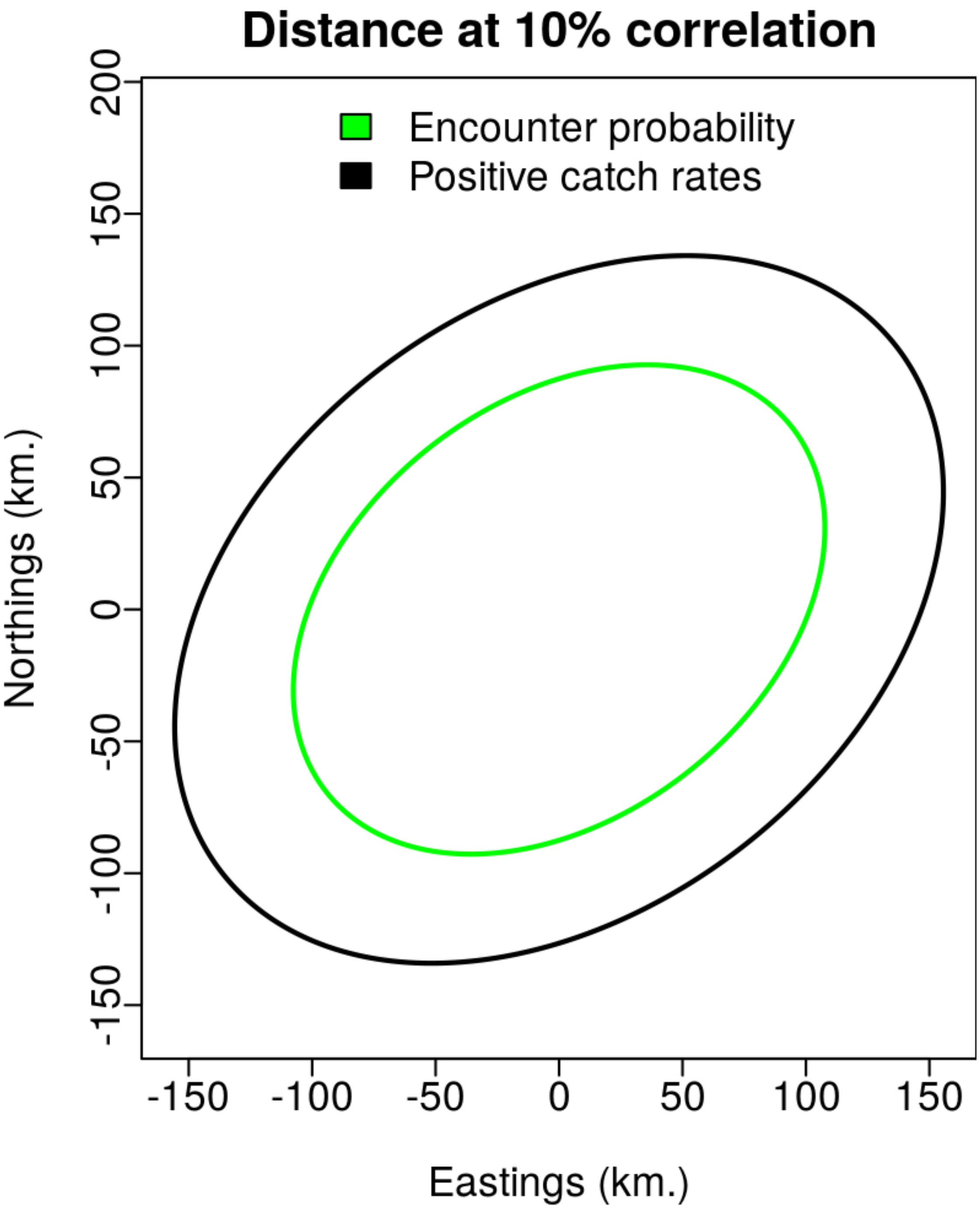

Figure S9: Estimates of distances at 10 % correlation from the Matérn covariance function for encounter probability and positive catch rates.

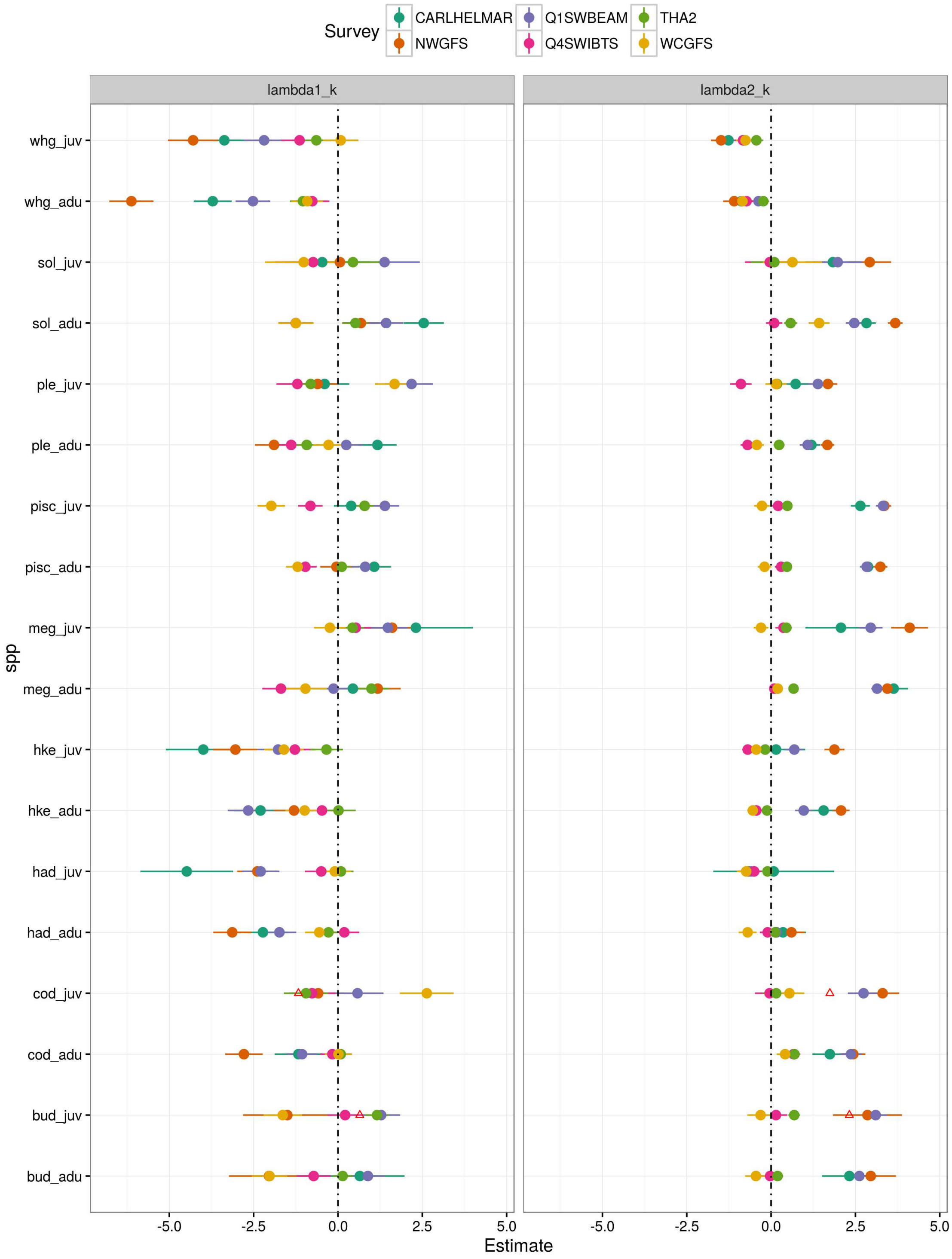

Figure S10: Fixed effect estimates for surveys for each species-group. Point estimate as a circle with ±1.96 x SE shown as a line. Note all values within a species-group are relative to the CEXP survey.

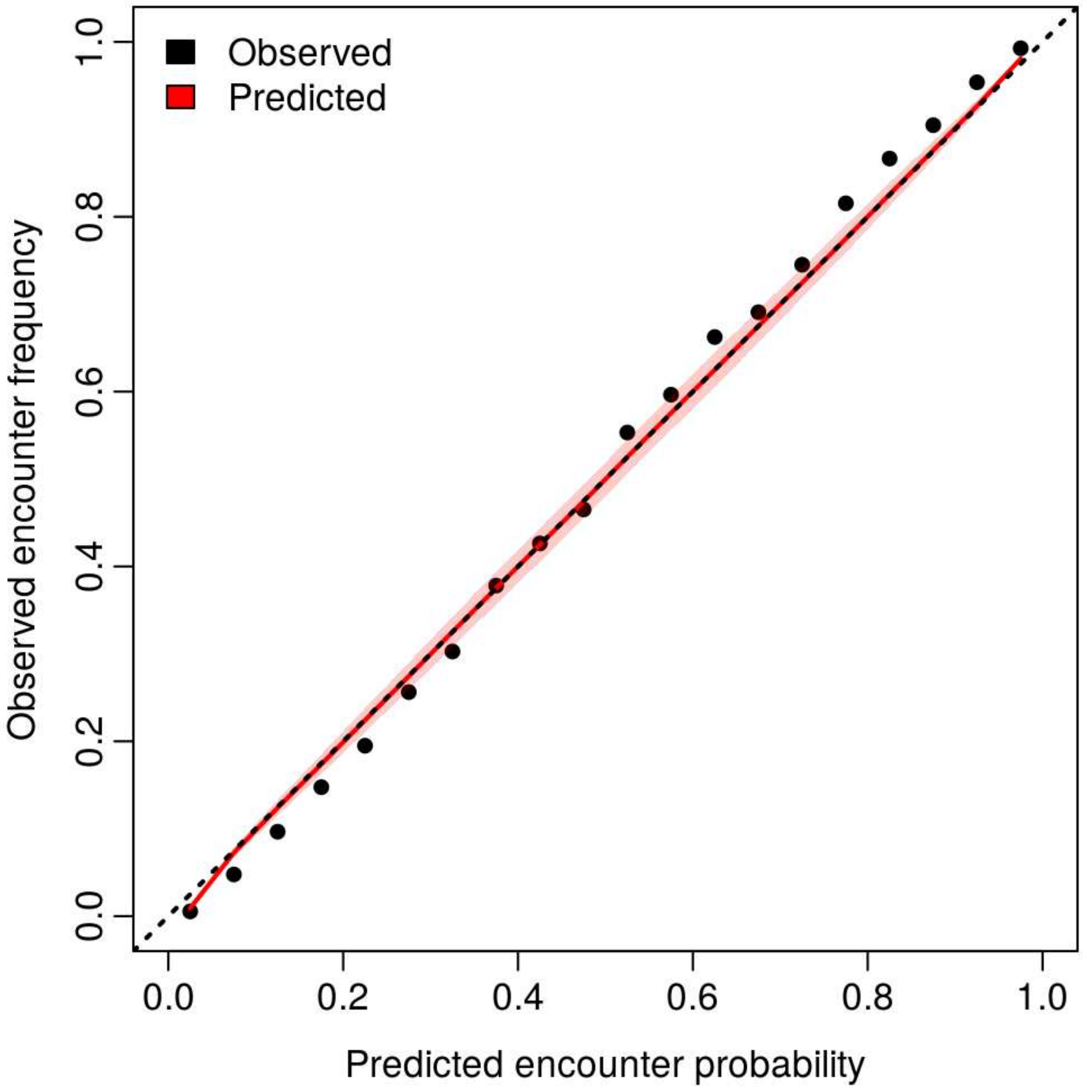

Figure S11: Model diagnostics output showing correlation between the predicted encounter probability and the data.

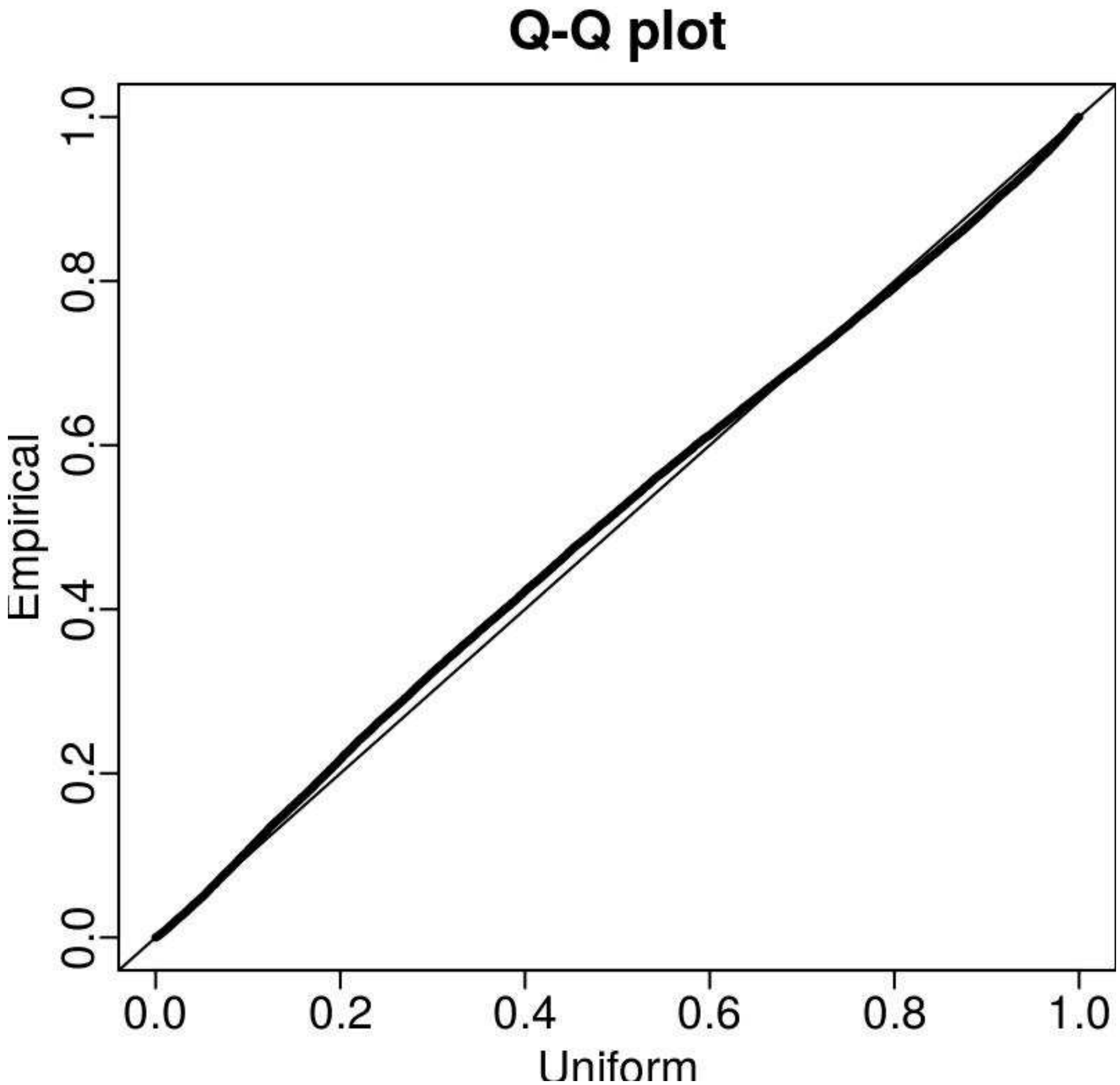

Figure S12: Model diagnostics output showing the Q-Q plot for the positive catch rates.

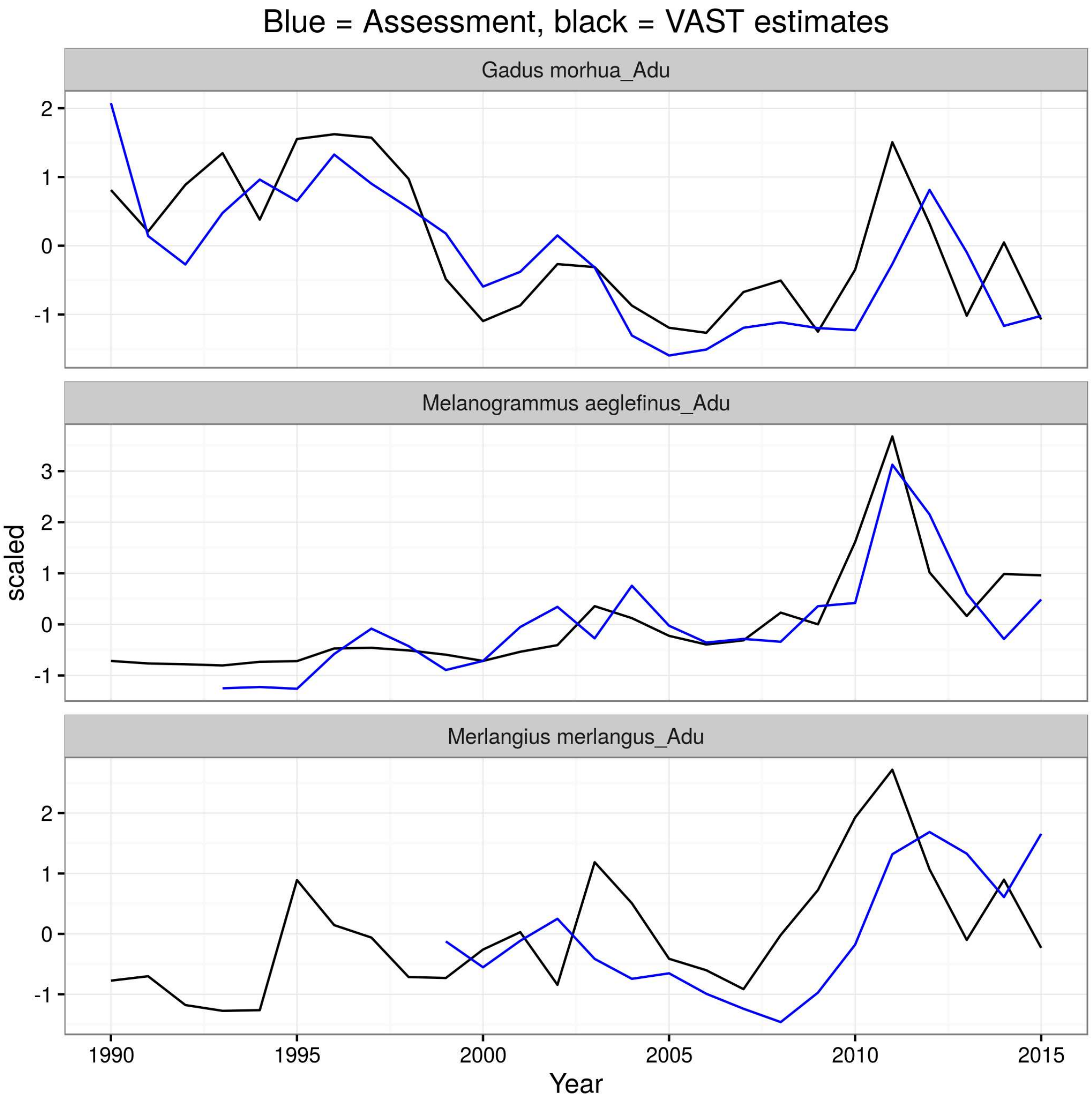

Figure S13: Comparison between the standardised index from the VAST output and the standardised spawning stock biomass (SSB) from the assessments for cod, haddock and whiting.

Table S1: List of survey codes, names and brief description.

Survey code	Name	Gear	Temporal extent
CEXP	Celtic Explorer (IE)	Otter trawl	2003 - 2015
CARLHELMAR	Carlhelmar (UK)	Commercial beam trawl	1989 - 2013
NWGFS	North West groundfish survey (UK)	Beam trawl	1988 - 2015
Q1SWBEAM	Quarter 1 south-west beam trawl survey (UK)	beam trawl	2006 - 2015
Q4SWIBTS	Quarter 4 south-west international bottom trawl survey (UK)	Otter trawl	2003 - 2010
THA2	EVHOE survey on Thalasa (FR)	Otter trawl	1997 - 2015
WCGFS	Western channel groundfish survey (UK)	Otter trawl (Portugese high headline)	1982 - 2004

Table S2: List of species codes, names and minimum conservation reference size used to separate juvenile and adult fish.

Species code	Common name	Species	MCRS (cm)
juv	Juvenile		
adu	Adult		
bud	Black bellied anglerfish	*Lophius budgessa*	32*
cod	Atlantic cod	*Gadus morhua*	35
had	Atlatic haddock	*Melanogrammus aeglefinus*	30
hke	Atlantic hake	*Merluccius merluccius*	27
meg	Megrim	*Lepidorhombus whiffiagonis*	20
pisc	White bellied anglerfish	*Lophius piscatorius*	32*
ple	European Plaice	*Pleuronectes platessa*	27
sol	Common sole	*Solea solea*	24
whg	Atlantic whiting	*Merlangius merlangus*	27

*Anglerfish species estimated based on a 500g minimum marketing weight

Table S3: Description of model variants and AIC / BIC.

Model	Description	No fixed parameters	No random parameters	AIC	BIC
H0	Vessel random effects, no covariates	1462	129276	125954	140187
H1	With fixed gear effect, no density covariates	1674	129276	116012	132309
H2	With fixed gear effect, substrate and depth density covariates	1688	129276	116013	132446
